# Essential role of *Salmonella* Enteritidis DNA adenine methylase in modulating inflammasome activation

**DOI:** 10.1186/s12866-020-01919-z

**Published:** 2020-07-28

**Authors:** Yaxin Guo, Dan Gu, Tingting Huang, Liyan Cao, Xinyu Zhu, Yi Zhou, Kangru Wang, Xilong Kang, Chuang Meng, Xinan Jiao, Zhiming Pan

**Affiliations:** 1 Jiangsu Key Laboratory of Zoonosis, Yangzhou University, Yangzhou, 225009 Jiangsu China; 2 Jiangsu Co-innovation Center for Prevention and Control of Important Animal Infectious Diseases and Zoonoses, Yangzhou University, Yangzhou, 225009 Jiangsu China; 3 Key Laboratory of Prevention and Control of Biological Hazard Factors (Animal Origin) for Agrifood Safety and Quality, Ministry of A griculture of China, Yangzhou University, Yangzhou, Jiangsu China; 4 Joint International Research Laboratory of Agriculture and Agri-product Safety of the Ministry of Education, Yangzhou University, Yangzhou, Jiangsu China

**Keywords:** *Salmonella* Enteritidis, DNA adenine methylase, Inflammasome, Cytotoxicity, Caspase-1, Interleukin-1β

## Abstract

**Background:**

*Salmonella* Enteritidis (SE) is one of the major foodborne zoonotic pathogens of worldwide importance which can induce activation of NLRC4 and NLRP3 inflammasomes during infection. Given that the inflammasomes play an essential role in resisting bacterial infection, *Salmonella* has evolved various strategies to regulate activation of the inflammasome, most of which largely remain unclear.

**Results:**

A transposon mutant library in SE strain C50336 was screened for the identification of the potential factors that regulate inflammasome activation. We found that T3SS-associated genes *invC*, *prgH*, and *spaN* were required for inflammasome activation in vitro. Interestingly, C50336 strains with deletion or overexpression of Dam were both defective in activation of caspase-1, secretion of IL-1β and phosphorylation of c-Jun N-terminal kinase (Jnk). Transcriptome sequencing (RNA-seq) results showed that most of the differentially expressed genes and enriched KEGG pathways between the C50336-VS-C50336Δ*dam* and C50336-VS-C50336::*dam* groups overlapped, which includes multiple signaling pathways related to the inflammasome. C50336Δ*dam* and C50336::*dam* were both found to be defective in suppressing the expression of several anti-inflammasome factors. Moreover, overexpression of Dam in macrophages by lentiviral infection could specifically enhance the activation of NLRP3 inflammasome independently via promoting the Jnk pathway.

**Conclusions:**

These data indicated that Dam was essential for modulating inflammasome activation during SE infection, there were complex and dynamic interplays between Dam and the inflammasome under different conditions. New insights were provided about the battle between SE and host innate immunological mechanisms.

## Background

*Salmonella enterica subsp. enterica* is a Gram-negative facultative intracellular bacterial pathogen that causes over 1.3 billion illnesses and 200,000 deaths in humans annually worldwide [1]. Salmonellosis ranges from self-limiting gastroenteritis to lethal bacteremia in immunocompromised individuals [2]. Although more than 2500 different serotypes have been discovered, *Salmonella enterica subsp. enterica* serotype Enteritidis (SE) is one of the most important serovars transmitted from animals to humans and has replaced *Salmonella enterica subsp. enterica* serotype Typhimurium (ST) as the primary cause of salmonellosis globally since 1980 [3]. SE has been one of the major causes of outbreaks of food poisoning, poultry meat, eggs, and products are considered to be the major source of infection [4]. SE infection is a major public health problem, which could also cause severe economic losses in the poultry industry worldwide [5]. The elucidation of the host anti-infection immunity mechanism and development of new therapeutic strategies against SE infection would be of great importance.

After invading the host through the gastrointestinal tract, *Salmonella* traverses the epithelial barrier either through invasion of intestinal epithelial and microfold cells overlying Peyer’s Patches or capture by CD18^+^ immune cells directly from the intestinal lumen [6]. *Salmonella* cells that survive and replicate within macrophages are critical for the infection to extend beyond the intestinal mucosa [7]. The ability of *Salmonella* to invade host cells is dependent upon the *Salmonella* pathogenicity island-1 (SPI-1)-encoded type III secretion system (T3SS) which injects effector proteins into the host cell cytosol to modulate cellular responses, while the SPI-2 T3SS subsequently translocates virulence proteins to subvert the bactericidal properties of macrophages and create a specialized *Salmonella*-containing vacuole (SCV) for replication [8]. This special intracellular lifestyle exposes *Salmonella* to detection by inflammasomes, which have been identified as playing an essential role in the innate immune response against *Salmonella* infection [9].

Inflammasomes are cytoplasmic multi-protein sensors consisting of varying protein components, generally including pattern recognition receptors (PRRs, such as Nod-like receptors (NLRs) and melanoma 2 (AIM2)-like receptor), an adaptor protein ASC (apoptosis-associated speck-like proteins containing caspase recruitment domains), and an effector subunit caspase (primarily caspase-1) [10]. It is widely reported that NLRP3 and NLRC4 inflammasomes are activated during *Salmonella* infection [11]. After *Salmonella* invades host cells, the T3SS apparatus and pore-forming toxins are detected by NLRP3, while NLRC4 recognizes bacterial flagellin, T3SS needle, and basal rod proteins [12]. Inflammasome assembly is triggered to activate pro-caspase-1 cleavage, leading to cleavage and secretion of caspase-1-dependent pro-inflammatory cytokines interleukin-1β (IL-1β) and IL-18 and rapid lytic cell death known as pyroptosis [13]. *Salmonella* cells are subsequently released and exposed to neutrophil-mediated killing, which are recruited by IL-1β [14].

A previous study demonstrated that mice deficient in caspase-1, IL-1β, or IL-18 succumbed to infection earlier than wild type (WT) mice and had significantly higher *Salmonella* burdens in the Peyer’s patches, mesenteric lymph nodes, and spleens [15]. In addition, genetic ablation of both NLRC4 and NLRP3 in mice leads to increased susceptibility to *Salmonella* infection [16]. Given the emphasis on inflammasomes in resisting bacterial infection, *Salmonella* has evolved multiple mechanisms to avoid or delay inflammasome activation [17]. To evade NLRC4 detection, flagellin and SPI-1 are strongly repressed in the intracellular environment through the PhoP-PhoQ regulatory system of *Salmonella* [18]. A recent study reported that *Salmonella* uses the SPI-1 T3SS effector SopB to suppress NLRC4 inflammasome activation by preventing ASC oligomerization [19]. *Salmonella* can also down-regulate NLRC4 and interfere with other host pathways to regulate inflammasome activation, such as autophagy [20]. In addition, *Salmonella* tricarboxylic acid cycle enzymes have been reported to limit NLRP3 inflammasome-dependent antibacterial immune responses through mitochondrial reactive oxygen species [21]. However, the many strategies of *Salmonella* regulation of the activation of the inflammasome remain largely unknown.

Until now, research on the interplay between *Salmonella* and inflammasomes has focused on ST, with SE inflammasomes still poorly understood. To identify potential factors involved in regulating the activation of the inflammasome, we constructed a transposon mutant library in SE strain C50336 and screened for increased or decreased inflammasome activation in the mouse macrophage cell line J774A.1. Sequencing of candidate mutants identified *dam*, the gene encoding DNA adenine methylase, which is an essential factor for *Salmonella* virulence [22]. Intriguingly, both deletion and overexpression of Dam failed to induce inflammasome activation, and RNA-seq assays suggested that the absence and overexpression of Dam had similar effects on infected macrophages. Notably, overexpression of Dam in J774A.1 cells by lentiviral infection promoted activation of the NLRP3 inflammasome. Collectively, these results indicated that SE Dam plays a critical role in inflammasome activation during infection.

## Results

### Screening candidate mutants for alterations in inflammasome activation

**Fig. 1:**
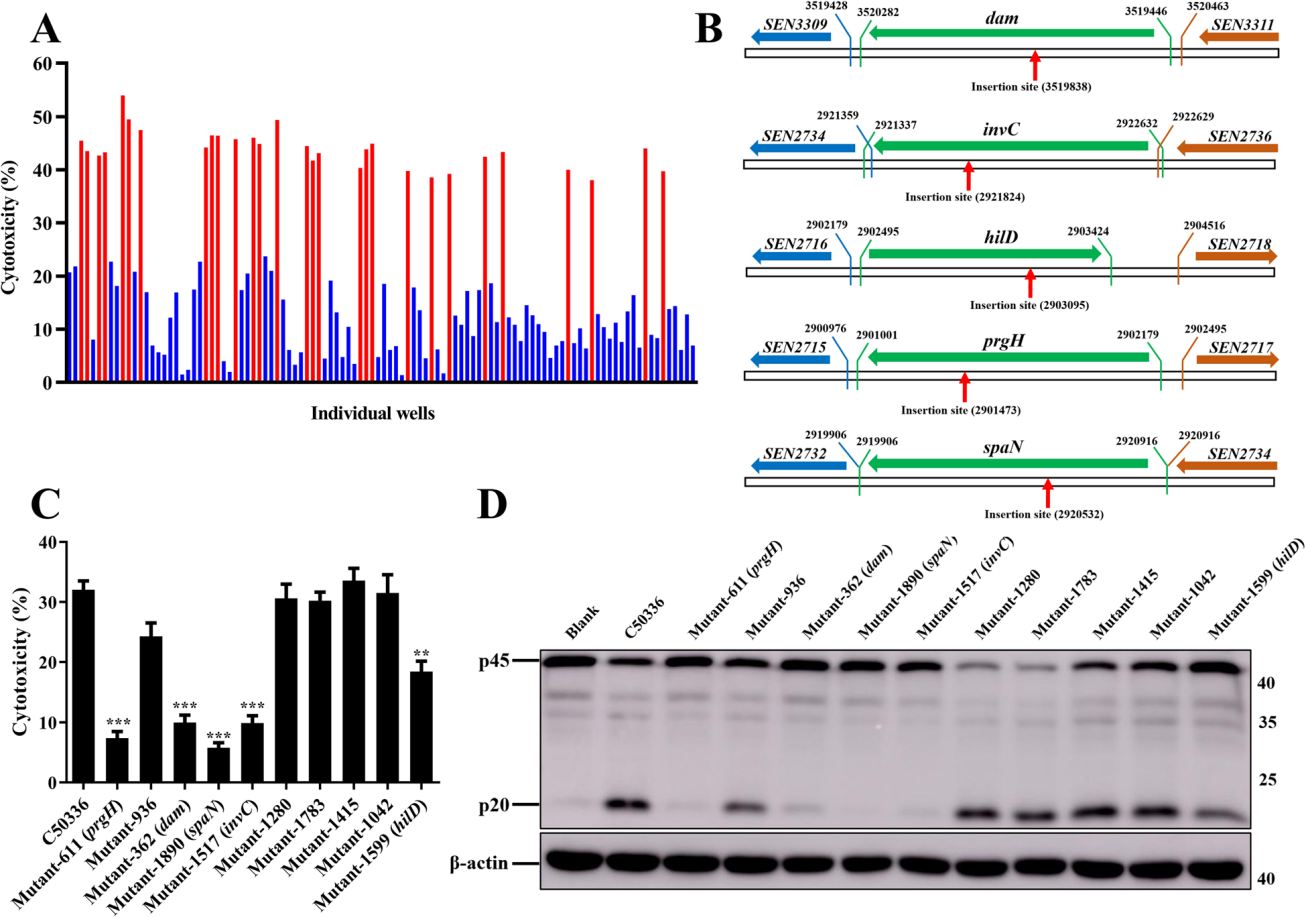
Two rounds of screening to identify the genes involved in regulating inflammasome activation in vitro. **a**. J774A.1 cells were pre-treated with LPS (1 μg/mL, 5 h) and then infected with C50336 TnpSC189 transposon mutants at an MOI of 20 for 4 h. The ratio of cell death was evaluated by the release of LDH in supernatants of infected cells. The Z score was calculated for each individual well in a 48-well cell plate, and a Z score ≤ − 2 or ≥ 2 was considered significant. Twenty-nine mutants induced significantly higher cytotoxicity levels (red, Z score ≥ 2) and 77 mutants induced significantly lower cytotoxicity levels (blue, Z score ≤ − 2). **b**. The transposon insertion sites of each candidate transposon mutants. Horizontal arrows indicate the direction of gene expression. The green genes represent the candidate genes, the blue genes represent the upstream genes of the candidate genes, the yellow genes represent the downstream genes of the candidate genes. The numbers represent the initial or terminal position in the *Salmonella* Enteritidis genome of each gene. The red vertical arrows represent the transposon insertion site of each candidate transposon mutants. **c**. J774A.1 cells were pre-treated with LPS (1 μg/mL, 5 h) and then infected with candidate transposon mutants at an MOI of 20 for 4 h, uninfected cells was used as a negative control (Blank). The ratio of cell death was evaluated by the release of LDH in supernatants of infected cells. The activation of caspase-1 (p20) was examined via western blot **d**. β-actin was blotted as a loading control. Molecular mass markers in kDa are indicated on the right. Original images of immunoblotting were shown in Fig. S2

To screen potential genes involved in modulating the activation of the inflammasome, a *mariner*-based transposon (TnpSC189) mutant library in SE strain C50336 was generated. Thus far, 2062 transposon mutants have been tested individually, and candidate mutants were selected by increased or decreased Lactate dehydrogenase (LDH) release relative to the C50336 parental strain 4 h after infection of the mouse macrophage cell line J774A.1. Following the first round of screening, 106 mutants were considered candidates for the second round of validation (Fig. 1a). The cytotoxicity induced by 29 mutants (Z score ≥ 2) was significantly higher than that induced by C50336, while 77 mutants (Z score ≤ − 2) induced significant lower cytotoxicity. To verify the selected candidates, each was tested individually in another screening round. The candidate mutants were rescreened in J774A.1 cells, LDH release was quantified, and caspase-1 activation was determined by western blot. The candidate transposon mutants 611, 362, 1890, 1517, and 1599 induced significant lower levels of cytotoxicity and activation of caspase-1 compared to WT (Fig. 1c and d). The transposon insertion sequencing of the candidate mutants identified these five genes were *dam*, *invC*, *hilD*, *prgH* and *spaN*, the transposon insertion sites were shown in Fig. 1b. The *dam* gene encodes a DNA adenine methylase, *invC* encodes a FliI/YscN family ATPase, *hilD* encodes a transcriptional regulator, *prgH* encodes a T3SS inner membrane ring protein, and *spaN* encodes an SPI-1 T3SS protein.

### Identification of candidate genes regulate the inflammasome activation

**Fig. 2:**
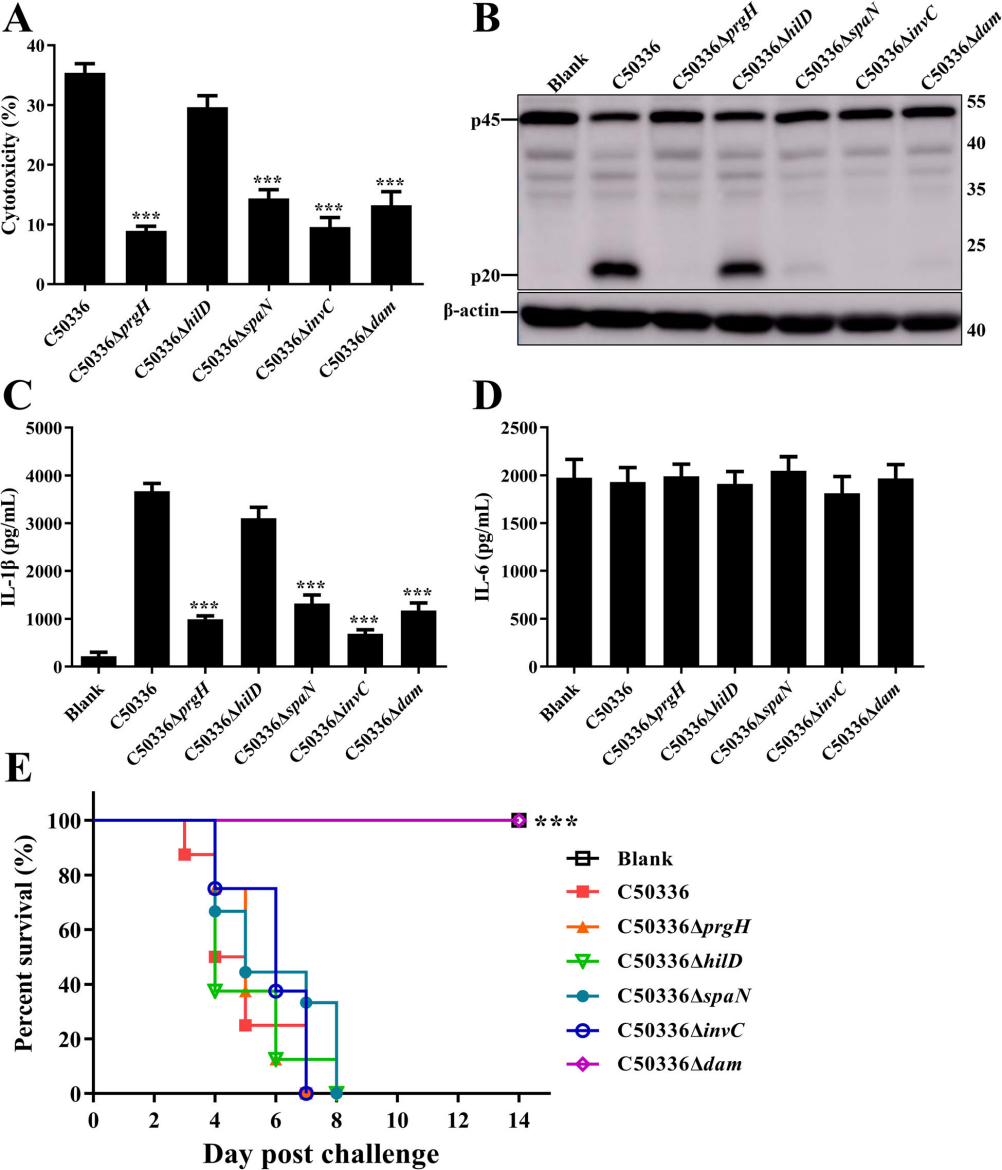
Deletion mutants of *dam*, *invC*, *prgH*, and *spaN* failed to induce inflammasome activation. J774A.1 cells were pre-treated with LPS (1 μg/mL, 5 h) and then infected with WT strain C50336 and *dam*, *invC*, *hilD*, *prgH*, and *spaN* gene deletion mutants at an MOI of 20 for 4 h, uninfected cells was used as a negative control (Blank). **a**. The ratio of cell death was evaluated by the release of LDH in supernatants of infected cells. **b**. The activation of caspase-1 (p20) was examined via western blot. β-actin was blotted as a loading control. Molecular mass markers in kDa are indicated on the right. Original images of immunoblotting were shown in Fig. S3. The production of IL-1β **c** and IL-6 **d** in supernatants were examined via ELISA. ***p < 0.001 for one-way ANOVA followed by Bonferroni’s multiple comparison test indicate significant findings in comparison with cells infected with WT strain C50336. Data are presented as mean ± SEM of triplicate samples per experimental condition from three independent experiments. **e**. C57BL/6 mice were intraperitoneally injected with PBS (negative control, Blank), C50336, and gene deletion mutants at a dose of 1 × 10^5^ CFU per mouse. The mortality was recorded over 14 dpc. ***, *p* < 0.001 compared with the C50336-infected group by log-rank (Mantel-Cox) test for the survival curve

To further investigate the function of these five genes in regulating inflammasome activation, the in-frame deletion mutants of *dam*, *invC*, *hilD*, *prgH*, and *spaN* genes were constructed. There was no significant difference in growth characteristics among WT strain, each mutant, complementation, and overexpression strains (Fig. S1). As shown in Fig. 2a, infection with *dam*, *invC*, *prgH*, and *spaN* mutant strains resulted in greatly decreased LDH release in comparison with J774A.1 cells infected by WT. We subsequently examined whether reduced cell death was caused by decreased inflammasome activation. As expected, the WT triggered strong caspase-1 activation and IL-1β expression in infected J774A.1 cells, while the production of cleaved caspase-1 p20 subunits and caspase-1-dependent cytokine IL-1β was significantly suppressed in cells infected with *dam*, *invC*, *prgH*, or *spaN* deletion mutant strains (Fig. 2b and c). Simultaneously, to further verify whether these genes can specifically influence the inflammasome pathway, we also measured the levels of non-inflammasome inflammatory cytokine IL-6 in supernatants. There was no significant difference in IL-6 secretion between WT- and deletion mutant strains-infected cells (Fig. 2d). These results suggested that *dam*, *invC*, *prgH*, and *spaN* specifically influenced inflammasome activation during SE infection in J774A.1 cells. In addition, the mouse was used as the animal model to determine the virulence of gene-deficient strains. None of the mice died in the C50336Δ*dam*-infected group, whereas all mice infected with other gene deletion mutants or WT C50336 (50) died within 8 days post-challenge (dpc) (Fig. 2e). No clinical symptoms were detected in the mice infected with C50336Δ*dam*, suggesting that Dam played an important role in the virulence of SE.

### Identification of proteins transferred into host cells

**Fig. 3:**
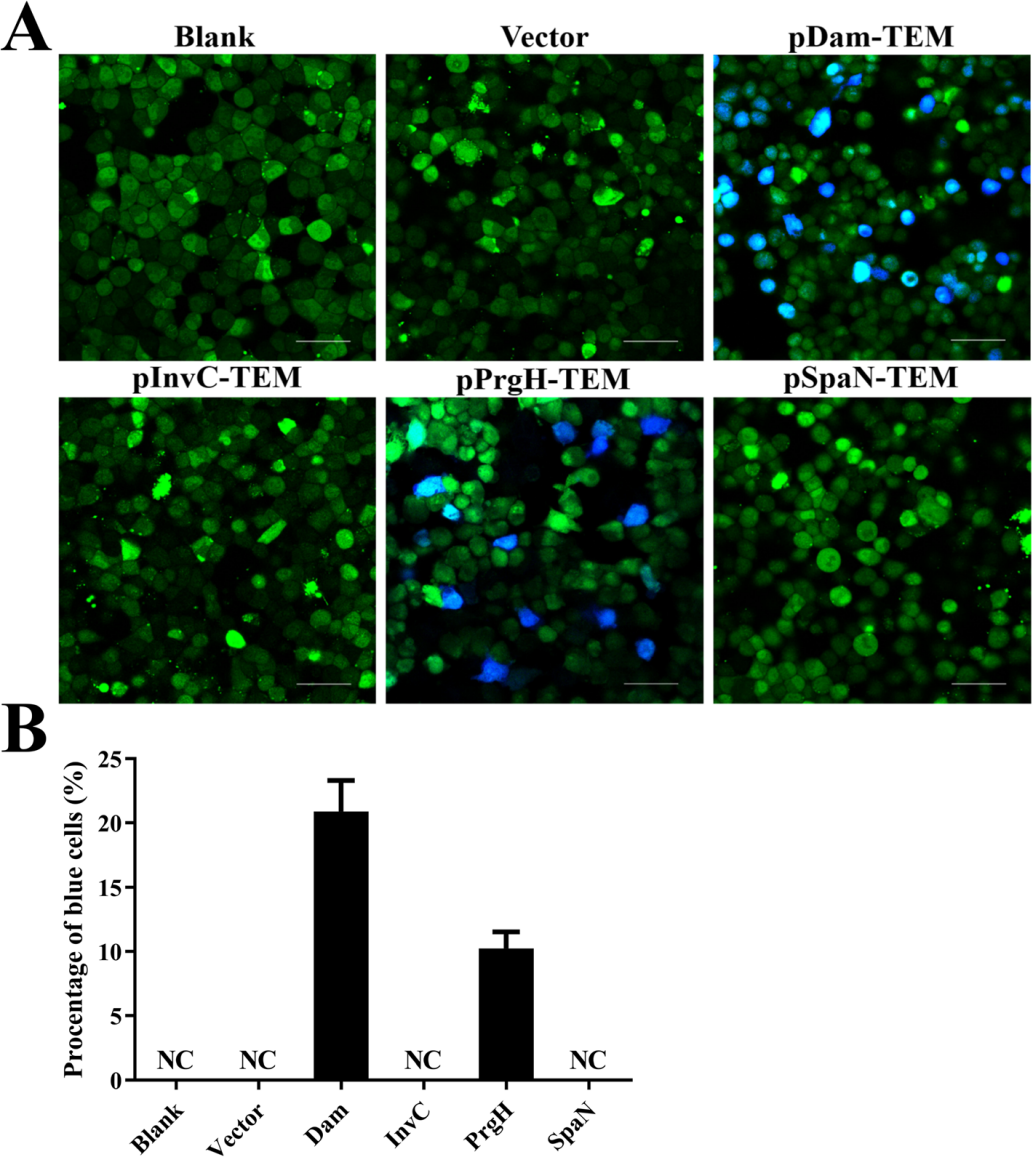
Target proteins Dam and PrgH were able to be translocated into the infected cells. HeLa cells were infected with WT C50336 bearing empty plasmid pCX340 or expressing different TEM-1 fusion proteins at an MOI of 100, uninfected HeLa cells was used as a negative control (Blank). Cells were washed and loaded with CCF2-AM after infection. **a**. Translocation of TEM-1 fusion proteins into the cell cytosol results in cleavage of CCF2-AM, emission of blue fluorescence revealed the activity of TEM β-lactamase, whereas uncleaved CCF2-AM emitted green fluorescence. Scale bar, 50 μm. **b**. The percentages of cells emitting blue fluorescence. For a particular cell well, six pictures were taken and approximately 1200–2000 cells were counted. Each picture was considered an independent observation and used to calculate the percentage of blue fluorescent cells. Data are presented as mean ± SEM of triplicate samples per experimental condition from three independent experiments

*Salmonella* injects various effector proteins into host cell cytosol to stimulate or interfere with cellular processes. To identify whether Dam, InvC, PrgH, and SpaN can be translocated into the cytosol of infected cells, β-lactamase TEM-1 was fused with each of these four proteins. The intracellular translocation was assayed via fluorescence resonance energy transfer (FRET) in infected HeLa cells (Fig. 3a). Uninfected HeLa cells and cells infected with C50336 contain an empty plasmid pCX340 appeared green, indicating the absence of TEM-1 activity in these cells. While, no blue fluorescence was detected in cells infected with C50336-pCX340-*invC* and C50336-pCX340-*spaN*, suggesting that the fusion proteins InvC-TEM and SpaN-TEM encoded by C50336 were not secreted and/or able to cleave the β-lactam ring of CCF2-AM. In contrast, infection with C50336 expressing Dam-TEM and PrgH-TEM resulted in a significant population of blue fluorescent cells. The transfer efficiency of PrgH-TEM was about 10.3% (Fig. 3b), while the expressed Dam-TEM was translocated at a higher efficiency (~ 21.1%). These data indicate that Dam and PrgH could be secreted and then translocated into the host cells during SE infection.

### Deletion of *dam* or *prgH* does not affect synthesis of inflammasome components

**Fig. 4:**
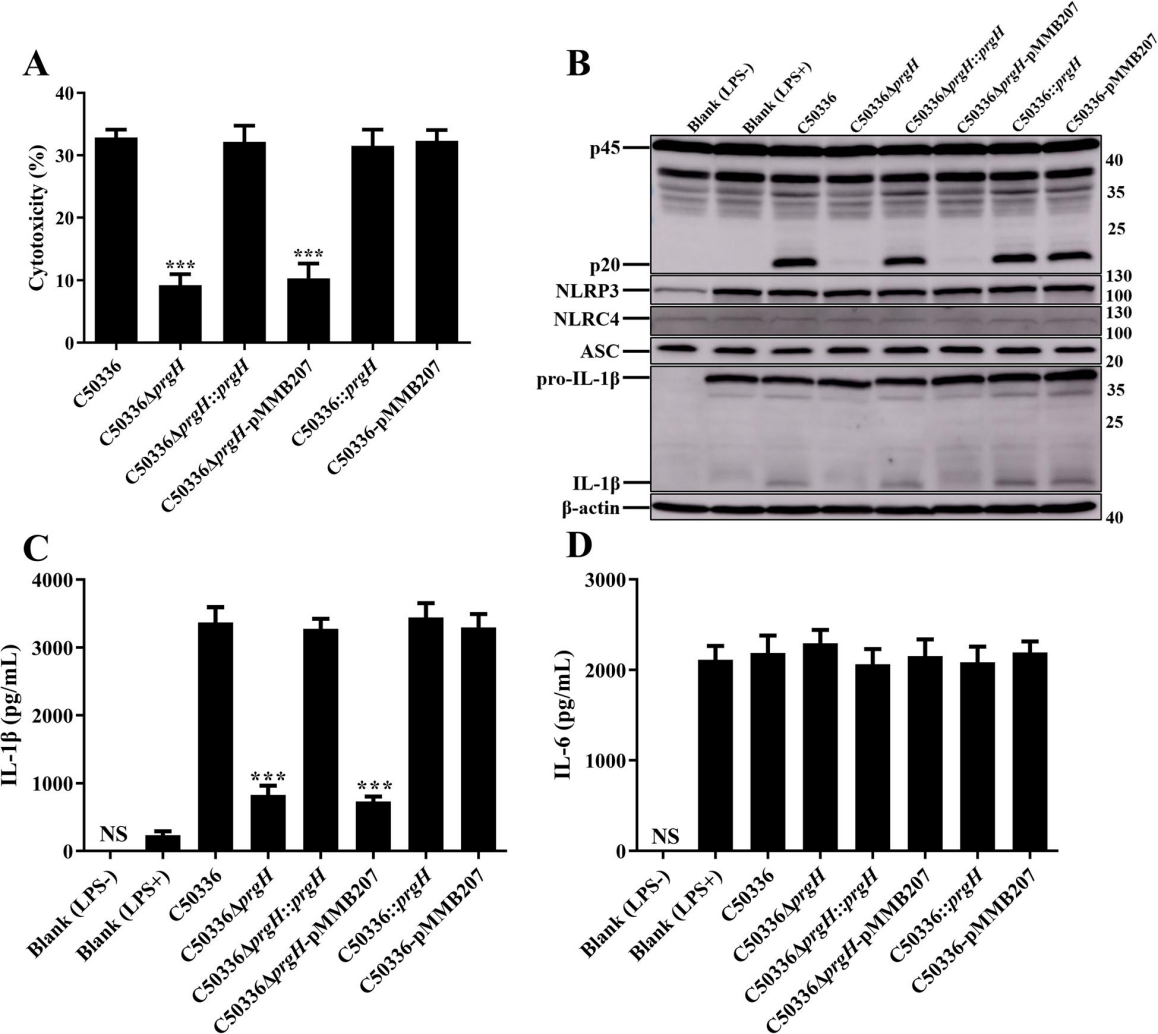
Deletion of *prgH* did not influence the synthesis of inflammasome components. C57BL/6 BMDMs were pre-treated with LPS (1 μg/mL) for 5 h (untreated and uninfected BMDMs was used as a negative control, Blank LPS-), and then infected with C50336, C50336Δ*prgH*, C50336Δ*prgH*::*prgH*, C50336Δ*prgH*-pMMB207, C50336::*prgH*, or C50336-pMMB207 at an MOI of 20 for 4 h, uninfected BMDMs was used as another negative control (Blank LPS+). Bacteria bearing pMMB207 plasmids were cultured with IPTG (0.5 mM). **a**. The ratio of cell death was evaluated by the release of LDH in the supernatants. **b**. The expression of caspase-1, NLRP3, NLRC4, ASC, and pro-IL-1β were analyzed by immunoblotting. β-actin was blotted as a loading control. Molecular mass markers in kDa are indicated on the right. Original images of immunoblotting were shown in Fig. S4. The production of IL-1β **c** and IL-6 **d** in the supernatants were examined via ELISA. ***p < 0.001 for one-way ANOVA followed by Bonferroni’s multiple comparison test indicate significant findings in comparison with cells infected with WT strain C50336. Data are presented as mean ± SEM of triplicate samples per experimental condition from three independent experiments

**Fig. 5:**
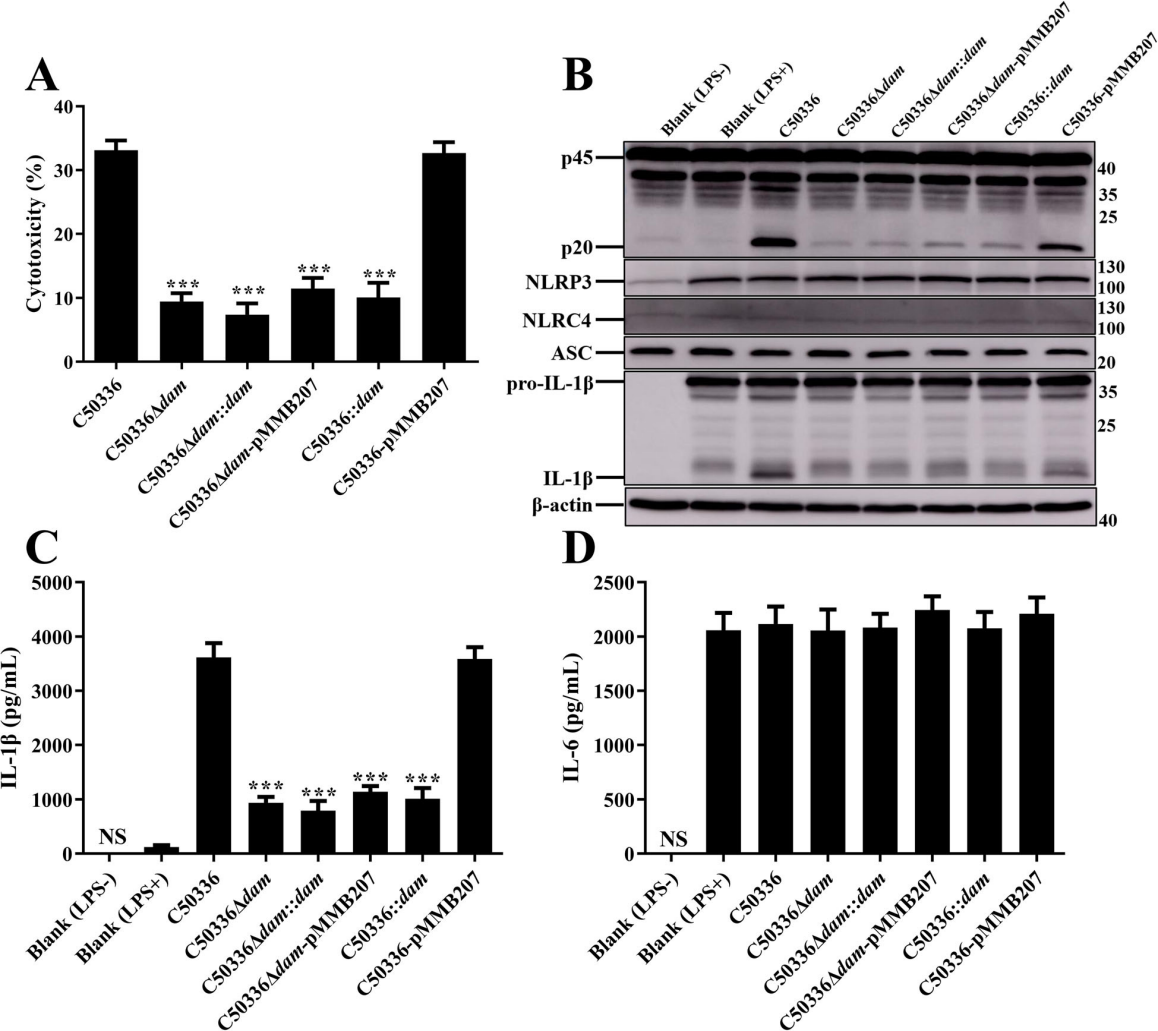
Overexpression of Dam inhibited inflammasome activation. C57BL/6 BMDMs were pre-treated with LPS (1 μg/mL) for 5 h (untreated and uninfected BMDMs was used as a negative control, Blank LPS-), and then infected with C50336, C50336Δ*dam*, C50336Δ*dam*::*dam*, C50336Δ*dam*-pMMB207, C50336::*dam*, or C50336-pMMB207 at an MOI of 20 for 4 h, uninfected BMDMs was used as another negative control (Blank LPS+). Bacteria bearing pMMB207 plasmids were cultured with IPTG (0.5 mM). **a**. The ratio of cell death was evaluated by the release of LDH in the supernatants. **b**. The expression of caspase-1, NLRP3, NLRC4, ASC, and pro-IL-1β were analyzed by immunoblotting. β-actin was blotted as a loading control. Molecular mass markers in kDa are indicated on the right. Original images of immunoblotting were shown in Fig. S5. The production of IL-1β **c** and IL-6 **d** in the supernatants were examined via ELISA. ***p < 0.001 for one-way ANOVA followed by Bonferroni’s multiple comparison test indicate significant findings in comparison with cells infected with WT strain C50336. Data are presented as mean ± SEM of triplicate samples per experimental condition from three independent experiments

Previous studies have shown that the expression of PrgH is regulated by Dam [23]. To further confirm the role of *dam* and *prgH* in regulating inflammasome activation, complementation strains and overexpression strains were constructed by employing the plasmid pMMB207. Primary bone marrow-derived macrophages (BMDMs) obtained from WT C57BL/6 mice were infected with indicated SE strains. The expression of NLRP3, NLRC4, ASC, and pro-IL-1β were analyzed by immunoblotting (Figs. 4 and 5). The results demonstrated that neither *dam* or *prgH* gene deletion nor overexpression could significantly suppress the expression of inflammasome components, indicating that the effect of Dam and PrgH on inflammasome activation was not achieved by influencing the synthesis of inflammasome components.

Similarly, LDH release was significantly decreased in BMDMs infected with C50336Δ*prgH* or C50336Δ*dam* as compared to WT strains, as was caspase-1 activation and IL-1β secretion (Figs. 4 and 5). The decreased cytotoxicity and expression of caspase-1 and IL-1β induced by C50336Δ*prgH* was recovered to a normal level when infected with C50336Δ*prgH*::*prgH*, whereas overexpression of *prgH* could not induce stronger inflammasome activation. However, the most interesting observation was that caspase-1 activation, IL-1β secretion, and LDH release were all obviously suppressed in BMDMs infected with complementation strain C50336Δ*dam*::*dam* and overexpression strain C50336::*dam* (Fig. 5), suggesting Dam may play a variety of roles in modulating inflammasome activation during SE infection. The addition of IPTG during the culture of bacteria containing plasmid pMMB207 had a great effect on promoting the expression of target proteins [24]. Moreover, we found that SE bearing plasmid pMMB207-*dam* failed to activate the inflammasome, and seemed reasonable because the expressions of Dam in both the complementation strain and overexpression strain were maintained at a high level in the presence of IPTG. The expression level of Dam may be a key factor in regulating inflammasome activation.

**Fig. 6:**
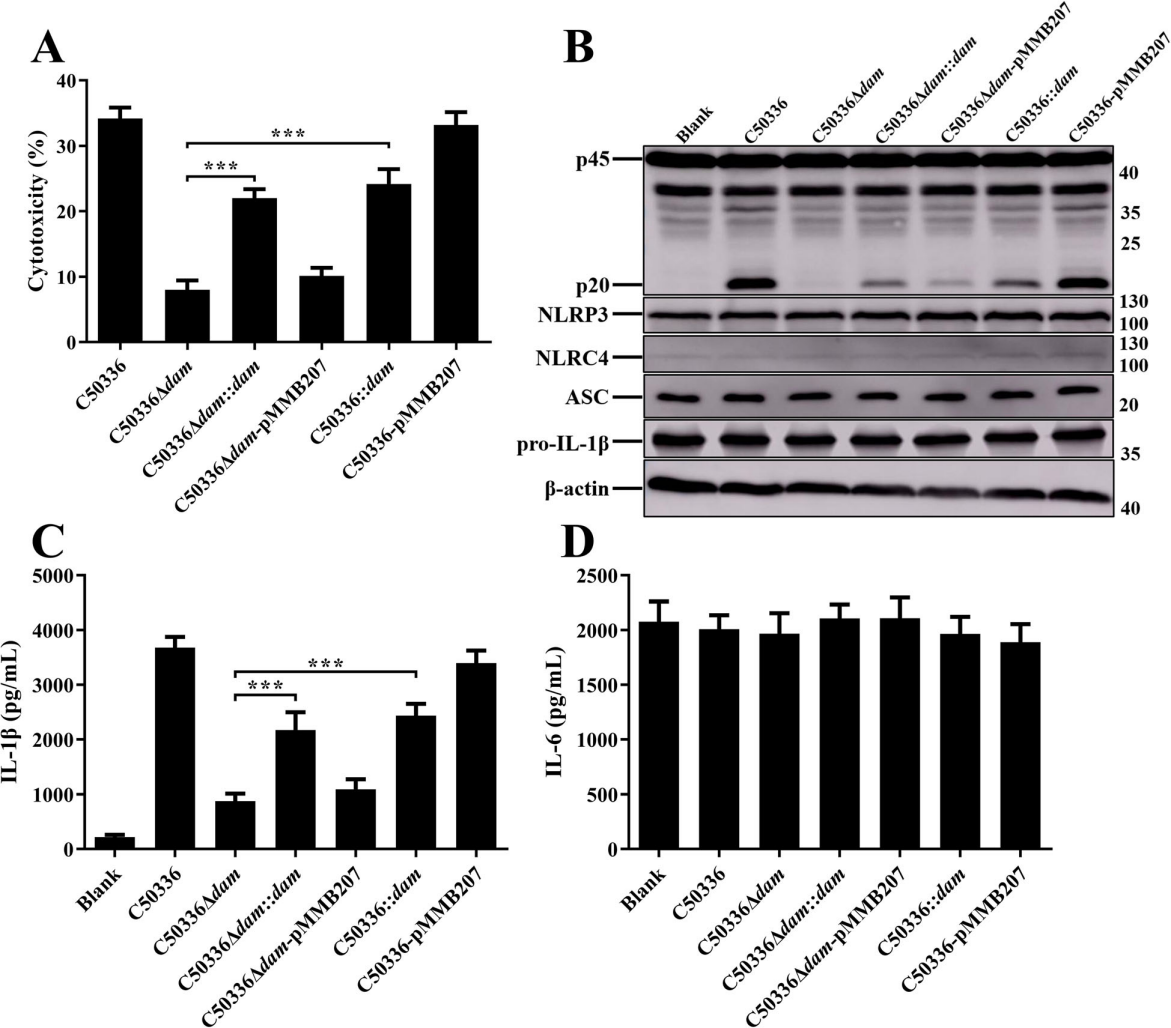
The ability of the *dam* complementation strain and overexpression strain cultured without IPTG to activate the inflammasome was improved. C57BL/6 BMDMs were pre-treated with LPS (1 μg/mL) for 5 h (untreated and uninfected BMDMs was used as a negative control, Blank LPS-), and then infected with C50336, C50336Δ*dam*, C50336Δ*dam*::*dam*, C50336Δ*dam*-pMMB207, C50336::*dam*, or C50336-pMMB207 at an MOI of 20 for 4 h uninfected BMDMs was used as another negative control (Blank LPS+). Bacteria bearing pMMB207 plasmids were cultured without IPTG. **a**. The ratio of cell death was evaluated by the release of LDH in the supernatants. **b**. The expression of caspase-1, NLRP3, NLRC4, ASC, and pro-IL-1β were analyzed by immunoblotting. β-actin was blotted as a loading control. Molecular mass markers in kDa are indicated on the right. Original images of immunoblotting were shown in Fig. S6. The production of IL-1β **c** and IL-6 **d** in the supernatants were examined via ELISA. ***p < 0.001 for one-way ANOVA followed by Bonferroni’s multiple comparison test indicate significant findings in comparison with cells infected with WT strain C50336. Data are presented as mean ± SEM of triplicate samples per experimental condition from three independent experiments

To verify this hypothesis, the complementation strains and overexpression strains cultured without IPTG were used to infect WT BMDMs. The results showed that the complementation strain induced a significant enhancement of cytotoxicity, bioactive IL-1β and caspase-1 secretion compared to C50336Δ*dam* (Fig. 6), although the ability of C50336Δ*dam*::*dam* to activate the inflammasome did not return to a normal level like C50336Δ*prgH*::*prgH* induction. Furthermore, the C50336::*dam* cultured without IPTG still induced relatively lower levels of cytotoxicity, IL-1β and caspase-1 compared with C50336-pMMB207. However, inflammasome activation induced by C50336::*dam* was significantly stronger than that induced by C50336Δ*dam.* Taken together, our results demonstrate that different expression levels of Dam could lead to different degrees of inflammasome activation during SE infection in vivo, suggesting that there may be a complex interaction between Dam and the inflammasome.

### Global transcriptome of J774A.1 cells infected with SE

**Fig. 7:**
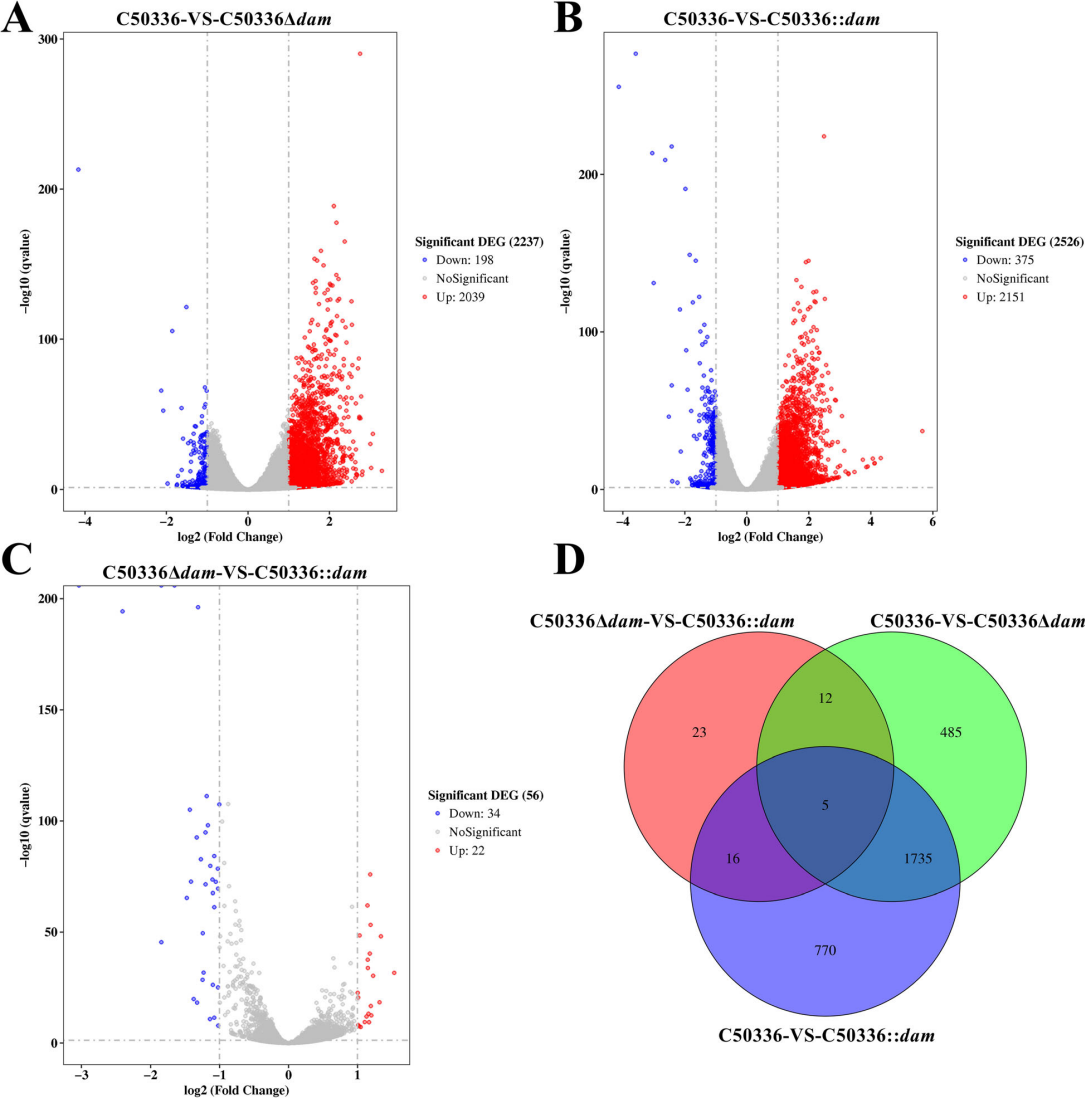
Differentially expressed genes (DEGs) between J774A.1 cells infected with SE. J774A.1 cells were pre-treated with LPS (1 μg/mL, 5 h) and then infected with C50336, C50336Δ*dam*, or C50336::*dam* (cultured with IPTG) at an MOI of 20 for 4 h. Each group comprised three independent replicates. The cells were then collected, and the total RNA was isolated for RNA sequencing. Volcano plot of all DEGs between the C50336-infected group and the C50336Δ*dam*-infected group **a**, the C50336-infected group and the C50336::*dam*-infected group **b**, and the C50336Δ*dam*-infected group and the C50336::*dam*-infected group **c**. Genes with log2 (fold change) ≤ − 1 and *p*-value < 0.05 are marked with blue dots, and genes with log2 (fold change) ≥ 1 and *p*-value < 0.05 are marked with red dots. **d**. Venn diagram displaying the number of overlapping DEGs in J774A.1 cells infected with different SE

Since the expression level of Dam was found to be important in regulating inflammasome activation, transcriptome analysis of J774A.1 cells infected with C50336, C50336Δ*dam*, or C50336::*dam* (cultured with IPTG) strains were carried out. The criteria for the selection of differentially expressed genes (DEGs) were false discovery rate (*p*-adjust) < 0.05 and a fold change value |log_2_Ratio| ≥ 1. In total, 2237 DEGs were identified between the C50336-infected and C50336Δ*dam*-infected groups, including 2039 up-regulated genes and 198 downregulated genes (Fig. 7a). Similarly, 2526 DEGs were found in the C50336-infected group compared with the C50336::*dam*-infected group, with 2151 genes up-regulated and 375 genes downregulated (Fig. 7b). Only 56 DEGs were screened between the C50336Δ*dam*-infected and the C50336::*dam*-infected groups (Fig. 7c), indicating that the gene expression pattern was similar between these two groups, which was further confirmed by Venn diagram analysis. As shown in Fig. 7d, 1738 overlapping genes were identified between the C50336-VS-C50336Δ*dam* and C50336-VS-C50336::*dam* groups, suggesting that the deletion and overexpression of Dam may had a similar effect in modulating cell responses during SE infection.

**Fig. 8:**
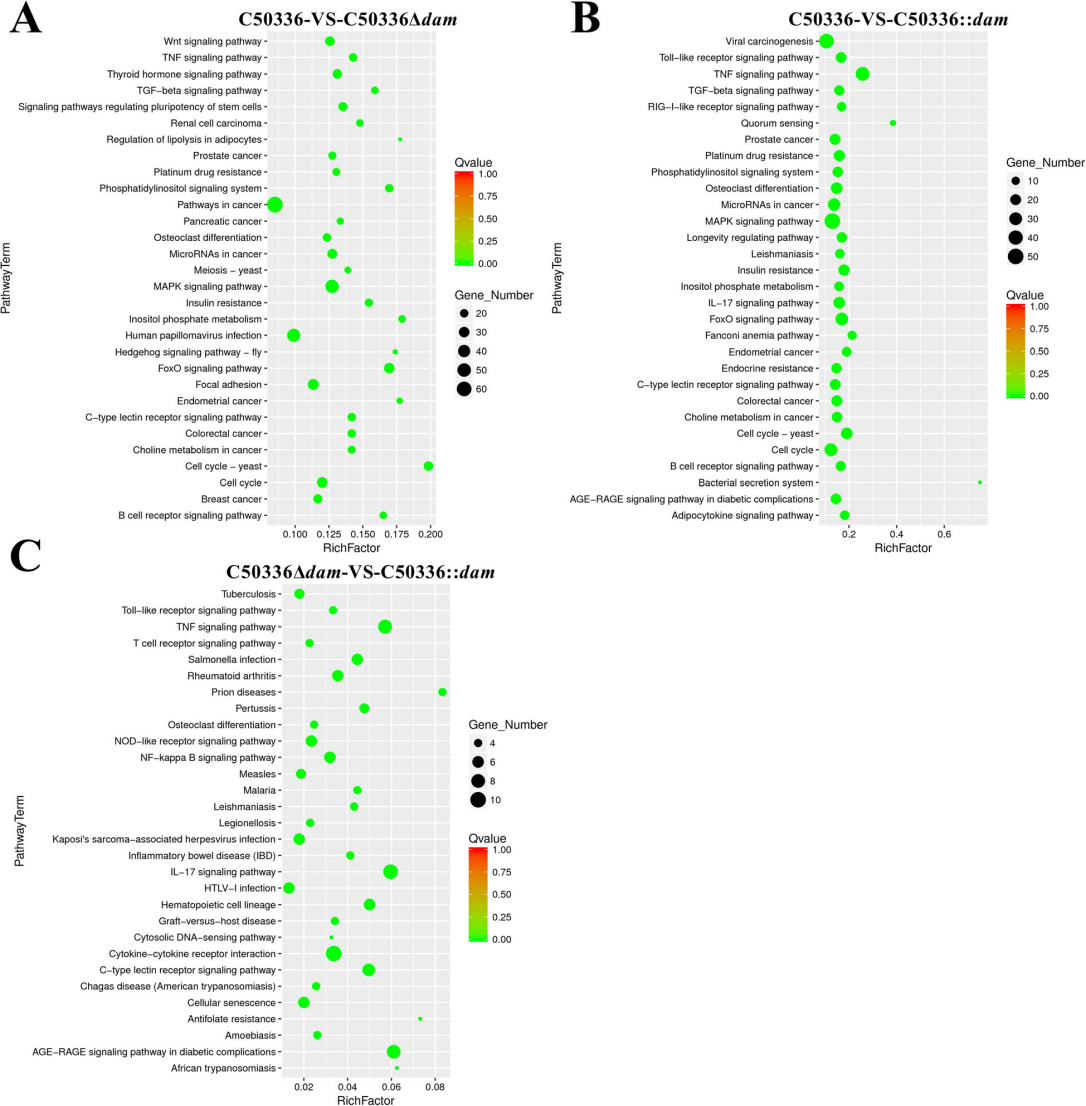
KEGG pathway enrichment of DEGs. Top 30 significant pathways involving DEGs. **a**. KEGG enrichment analysis of DEGs between the C50336-infected group and the C50336Δ*dam*-infected group. **b**. KEGG enrichment analysis of DEGs between the C50336-infected group and the C50336::*dam*-infected group. **c**. KEGG enrichment analysis of DEGs between the C50336Δ*dam*-infected group and the C50336::*dam*-infected group. The vertical axis represents the pathway category, and the horizontal axis represents the enrichment factor. Coloring indicates Q-value (high: red, low: green), and the lower Q-value indicates more significant enrichment. The point size indicates DEG numbers (more: big, less: small)

Kyoto Encyclopedia of Genes and Genomes (KEGG) pathway enrichment analysis was performed with the DEGs between these three groups to identify the potential pathways and genes affected by Dam. The 30 pathways that were most significantly enriched were shown in Fig. 8. The most significantly enriched pathways were related to cancer, mitogen-activated protein kinase (MAPK) signaling, and human papillomavirus infection between the C50336-infected group and the C50336Δ*dam*-infected group (Fig. 8a). The significantly enriched pathways were mainly involved in viral carcinogenesis, MAPK signaling, and tumor necrosis factor (TNF) signaling between the C50336-infected group and the C50336::*dam*-infected group (Fig. 8b). As expected, 18 overlapping pathways were found in the top 30 significantly enriched pathways between the C50336-VS-C50336Δ*dam* group and the C50336-VS-C50336::*dam* group, which further suggested that the deletion and overexpression of Dam may play a similar role in regulating cellular processes during SE infection. In contrast, the most significantly enriched pathways within a maximum of 10 DEGs were cytokine-cytokine receptor interaction, TNF signaling, IL-17 signaling, and AGE-RAGE signaling in diabetic complications between the C50336Δ*dam*-infected group and C50336::*dam*-infected group (Fig. 8c).

### Identification of the expression patterns of DEGs based on quantitative real-time PCR

**Fig. 9:**
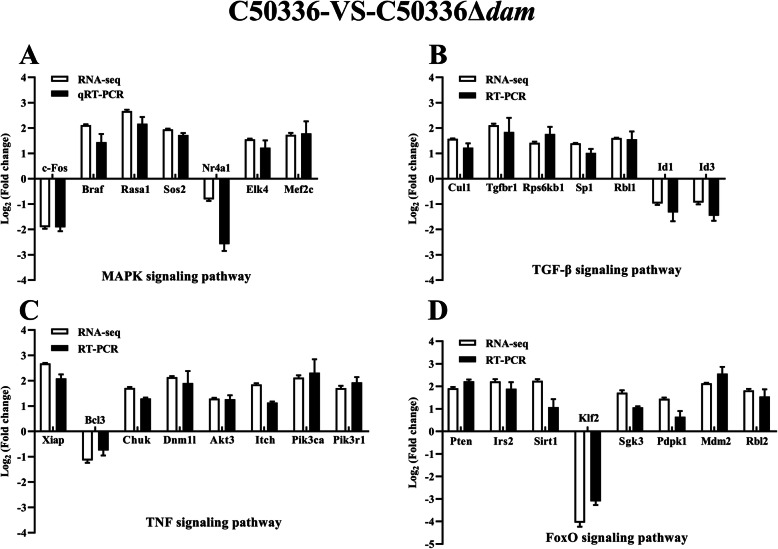
qRT-PCR validation of expression levels of the overlapping DEGs from MAPK signaling pathway **a**, TGF-β signaling pathway **b**, TNF signaling pathway **c**, and FoxO signaling pathway **d** in the C50336-VS-C50336Δ*dam* group. J774A.1 cells were pre-treated with LPS (1 μg/mL, 5 h) and then infected with C50336 or C50336Δ*dam* at an MOI of 20 for 4 h. Each group comprised three independent replicates. The cells were then collected, and the total RNA was isolated for qRT-PCR. The mRNA expression level was normalized against the mouse GAPDH transcript. Data are presented as mean ± SEM of log_2_ (fold change) of triplicate samples

**Fig. 10:**
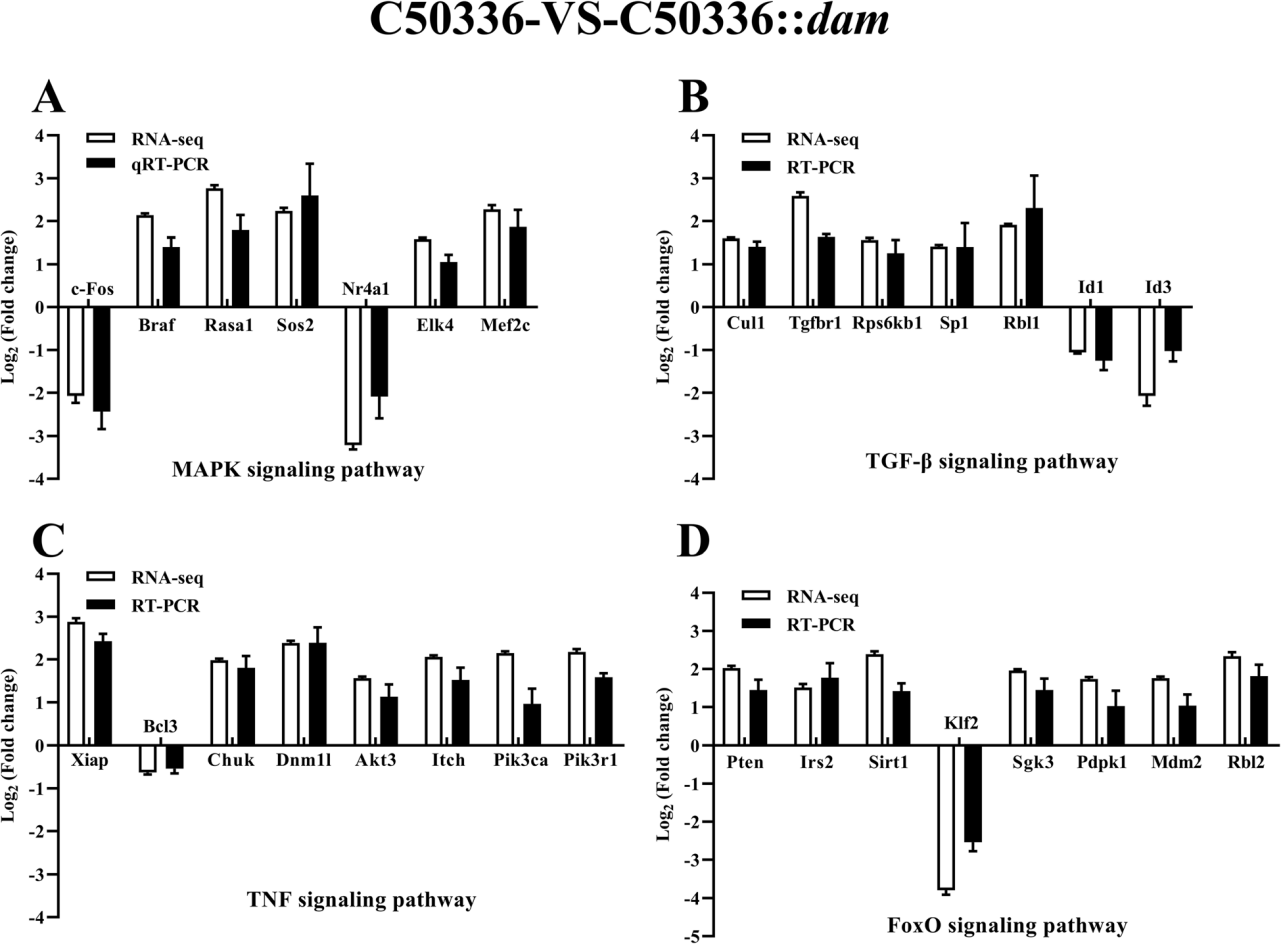
qRT-PCR validation of expression levels of the overlapping DEGs from MAPK signaling pathway **a**, TGF-β signaling pathway **b**, TNF signaling pathway **c**, and FoxO signaling pathway **d** in the C50336-VS-C50336::*dam* group. J774A.1 cells were pre-treated with LPS (1 μg/mL, 5 h) and then infected with C50336 or C50336::*dam* (cultured with IPTG) at an MOI of 20 for 4 h. Each group comprised three independent replicates. The cells were then collected, and the total RNA was isolated for qRT-PCR. The mRNA expression level was normalized against the mouse GAPDH transcript. Data are presented as mean ± SEM of log_2_ (fold change) of triplicate samples

We verifed RNA-seq results by selecting the differentially expressed genes in infected J774A.1 cells including 7 overlapping DEGs from MAPK signaling pathway, 7 overlapping DEGs from transforming growth factor-β (TGF-β) signaling pathway, 8 overlapping DEGs from TNF signaling pathway, and 8 overlapping DEGs from forkhead box, sub-group O (FoxO) signaling pathway between the C50336-VS-C50336Δ*dam* and C50336-VS-C50336::*dam* groups. Mouse glyceraldehyde-3-phosphate dehydrogenase (GAPDH) was used to normalize the gene expression. The expression of the selected overlapping DEGs was consistent with the results of RNA-seq in both C50336-VS-C50336Δ*dam* (Fig. 9) and C50336-VS-C50336::*dam* groups (Fig. 10). In addition, the fold changes of overlapping DEGs were similar between the C50336-VS-C50336Δ*dam* and C50336-VS-C50336::*dam* groups indicating that both deletion and overexpression of Dam played a similar role of regulating cellular processes during SE infection. These results demonstrated that the expression levels of Dam was essential for the regulation of these signaling pathways.

### Dam was required for Jnk pathway activation in vitro

**Fig. 11:**
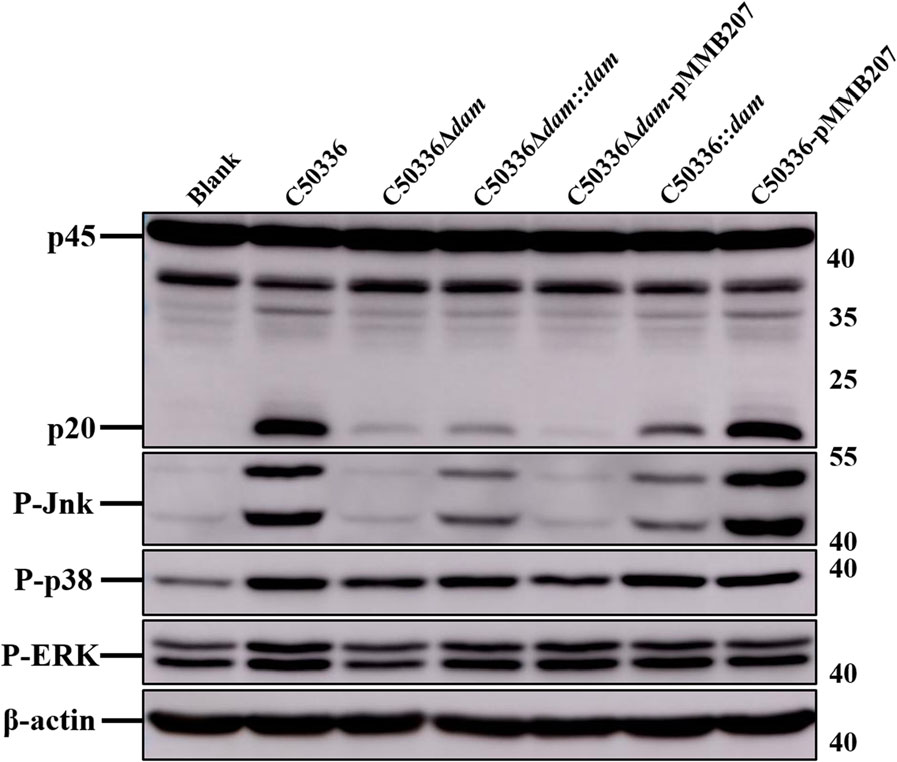
The deletion of Dam blocked the Jnk pathway in infected J774A.1 cells. J774A.1 cells were pre-treated with LPS (1 μg/mL, 5 h) and then infected with C50336, C50336Δ*dam*, C50336Δ*dam*::*dam*, C50336Δ*dam*-pMMB207, C50336::*dam*, or C50336-pMMB207 at an MOI of 20 for 4 h, uninfected cells was used as a negative control (Blank). Bacteria bearing pMMB207 plasmids were cultured without IPTG. The activation of caspase-1, phosphorylated Jnk (P-Jnk), phosphorylated p38 (P-p38), and phosphorylated ERK1/2 (P-ERK1/2) were analyzed by immunoblotting. β-actin was blotted as a loading control. Molecular mass markers in kDa are indicated on the right. Original images of immunoblotting were shown in Fig. S7

To further explore the mechanism of Dam regulates the activation of inflammasome, western blot was used to determined the activation of MAPK pathway based on the data of RNA-seq. The phosphorylation level of c-Jun N-terminal kinase (Jnk) induced by *dam* mutant strain was significantly lower than that induced by C50336 in J774A.1 cells, which was accompanied with the decreased activation of caspase-1 (Fig. 11). The Jnk phosphorylation was more significantly induced by *dam* complementation strain than by C50336Δ*dam*. In addition, the overexpression of Dam also induced a lower phosphorylation level of Jnk compared to WT. Furthermore, the phosphorylation level of p38 and extracellular regulated protein kinases 1/2 (ERK1/2) induced by C50336Δ*dam* was slightly lower than that induced by C50336, which was not significant. These results suggested that deletion of *dam* blocked the Jnk pathway in infected macrophages, which led to the inhibition of inflammasome activation.

### Dam promoted NLRP3 inflammasome activation independently via Jnk pathway in lentiviral infection assays

**Fig. 12:**
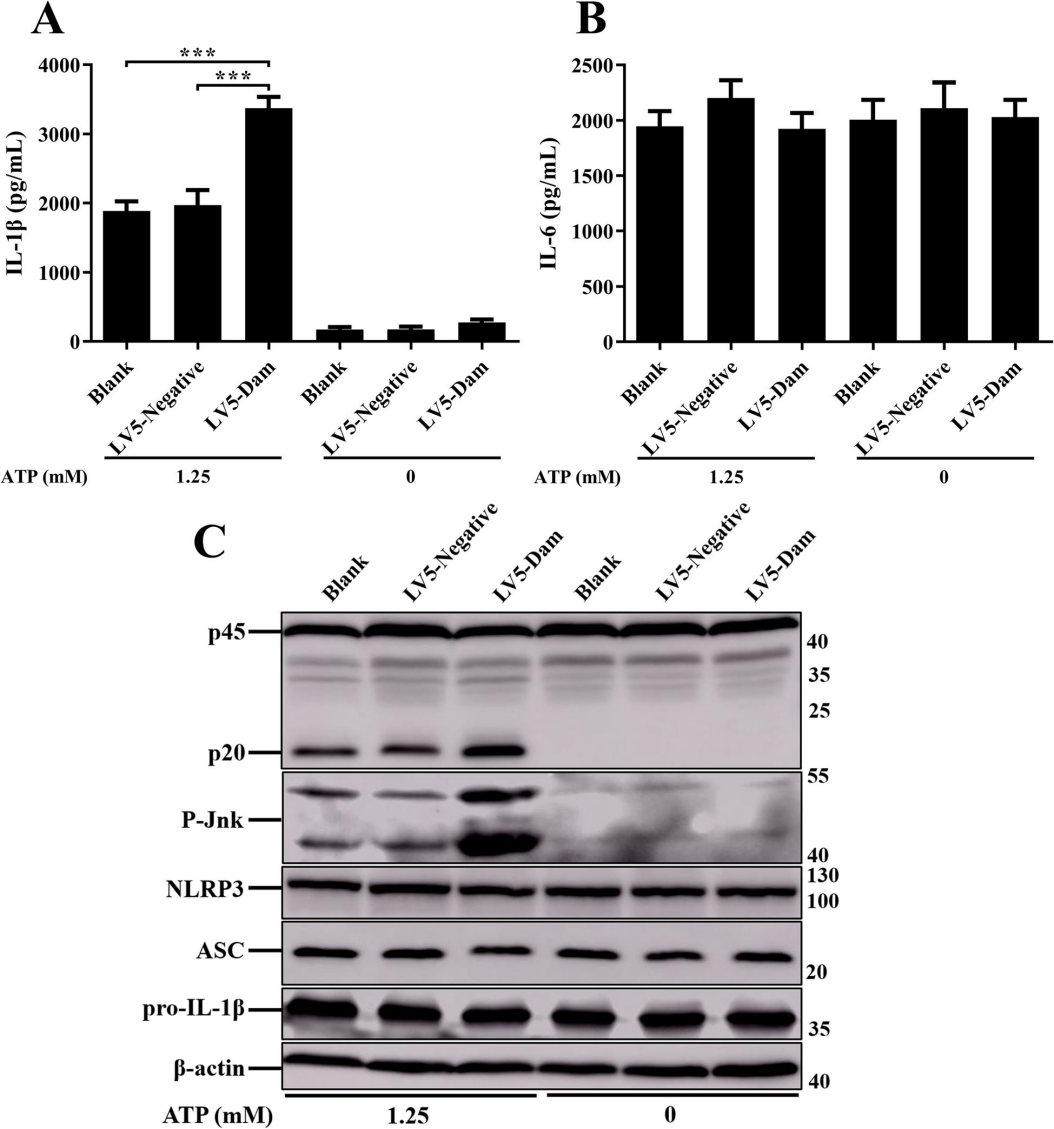
Dam independently promoted NLRP3 inflammasome activation. J774A.1 cells were transduced with LV5-Dam or LV5-negative lentivirus, untreated cells was used as a negative control (Blank). Cells were then pre-treated with LPS (1 μg/mL, 5 h) and stimulated with or without ATP (1.25 mM) for 1 h. The production of IL-1β **a** and IL-6 **b** in the supernatants were examined via ELISA. ***p < 0.001 for one-way ANOVA followed by Bonferroni’s multiple comparison test indicate significant findings in comparison with the control group. Data are presented as mean ± SEM of triplicate samples per experimental condition from three independent experiments. **c**. The expression of caspase-1, NLRP3, ASC, pro-IL-1β, and P-Jnk were analyzed by immunoblotting. β-actin was blotted as a loading control. Molecular mass markers in kDa are indicated on the right. Original images of immunoblotting were shown in Fig. S8

To further explore the effect of Dam on inflammasome activation, lentiviral vectors pGLV5-*dam* (EF-1aF/GFP&Puro) were constructed, and the lentivirus LV5-Dam particles were packaged and transfected into J774A.1 cells. To test the efficacy of lentivirus delivery into the macrophages, green fluorescent protein (GFP) expression was examined. The cells with LV5-Dam or LV5-negative lentivirus treatment exhibited robust GFP expression, indicating the effective infection of lentivirus in mice macrophages. After LPS priming, J774A.1 cells were stimulated with a classical NLRP3 inflammasome activator adenosine triphosphate (ATP, 1.25 mM). Significantly up-regulated inflammasome signals were detected in Dam-expressing J774A.1 cells, compared with those in negative lentivirus transduced cells and control cells (Fig. 12a and c). Furthermore, the expression of IL-6 and the synthesis of NLRP3 inflammasome components, including NLRP3, ASC, and pro-IL-1β were not affected by lentiviral infection (Fig. 12b and c). In addition, the deletion of *dam* failed to induce Jnk phosphorylation in infected J774A.1 cells, the phosphorylation of Jnk was also determined by immunoblotting with the infection of lentivirus LV5-Dam. As expected, there was a significant increased phosphorylation level of Jnk in Dam-expressing macrophages compared to those in negative lentivirus transduced cells and control cells. These results demonstrated that Dam could specifically enhance the NLRP3 inflammasome activation independently via promoting Jnk activation in macrophages.

## Discussion

Inflammatory caspases are multi-functional proteins, and activation of caspases not only play an essential role in mediating host defense against infection by pathogens but are also indispensable for the regulation of tumor development, metabolic syndromes, autoinflammatory disease, tissue repair, and cell survival [25]. NLRC4 and NLRP3 inflammasomes can detect exogenous and endogenous molecules that serve as indicators of *Salmonella* infection. Inflammasome activation triggers the secretion of the pro-inflammatory cytokines IL-1β and IL-18, as well as rapid cell lysis. Intracellular *Salmonella* is then released into the extracellular space, which can be a double-edged sword for both the host and bacteria. *Salmonella* exposed to neutrophil-mediated killing is extremely fragile [26], but the interaction also provides an opportunity for *Salmonella* to invade the intestinal epithelium or access the liver and spleen, which may promote systemic infection [27]. Inflammasomes play a key and multilayered role at distinct stages of immune defense against *Salmonella* infection, although unexpected redundancy was found among NLRP3 and NLRC4 in vivo [16]. To further understand the complex interplay of detection and evasion between SE and the inflammasome, a transposon library based on SE strain C50336 was generated and screened in this study. The mutant strains were identifed by increased or decreased LDH release compared to the WT C50336 parental strain after infection of J774A.1 cells in the first round of screening. Then, the candidate mutant strains were further rescreened for LDH release and activation of caspase-1 in the second round screening. Four T3SS related genes (*dam*, *invC*, *prgH*, and *spaN*) were identified after two rounds of screening, which the mutants of these four genes failed to activate the inflammasome indicating the importance of T3SS during modulating inflammasome activation. Our study offers new insights into understanding the interactions between SE and the inflammasome.

T3SS, as an important apparatus for inserting virulence factors into host cells, is often utilized by *Salmonella* to regulate the inflammasome responses. T3SS1 effector SopB was found to inhibit the activation of NLRC4 inflammasome during ST infection [19]. The SPI-2 T3SS is employed by ST to subvert human NLRP3 and NLRC4 inflammasome activation [28]. An essential component of T3SS is a highly conserved SPI-1 associated ATPase InvC in ST, which could couple with proton motive force to provide energy for the secretion process [29]. The deletion of *invC* gene resulted in a significant decrease in secretion and translocation of effectors, which is required for *Salmonella* entry into epithelial cells [30]. Additionally, we also identified the *spaN* gene, which was known to be a target of the invasion secretion system [31]. The invasion-defective *invC* mutant of ST failed to secrete SpaN upon contact with live epithelial cells [32], which could explain why InvC and SpaN mutant strains are defective in activation of the inflammasome. In addition, InvC and SpaN were both reported to be essential for *Salmonella* virulence in a murine model [33]. The InvC deficient mutant of ST significantly reduced virulence in swine [34].

The needle complex (NC) is a structure of connected oligomeric rings assembled by T3SS that spans both membranes of bacteria and host cells for the delivery of virulence effectors [35]. The earliest step in NC morphogenesis is the association of PrgH and PrgK to form a base substructure and inner membrane rings. PrgH and PrgK are demonstrated to be the only T3SS components that are absolutely required for the formation of NC [36]. The previous studies showed that the NC rod proteins PrgI and PrgJ can be detected by NLRC4, and resulting in rapid activation of the inflammasome [14]. However, the rod proteins could not be detected in a correct location within the T3SS apparatus [11]. If T3SS exports excess rod monomers from the bacterial cytosol or if rod monomers slough into the interior channel of the needle apparatus, NLRC4 can respond to such inadvertent translocation of PrgI and PrgJ monomer [37]. In this study, the β-lactamase-based translocation assay indicated that the PrgH of SE could be transferred into host cells (Fig. 3). The transfer efficient of PrgH-TEM fusion protein was lower, and this is understandable considering that PrgH is a structural protein of NC and may also depend on inadvertent translocation, like PrgJ [11]. The persistent overexpression of PrgJ resulted in strong detection via NLRC4 and the complete clearance of ST in mice [38]. However, overexpression of PrgH induced by exogenous plasmid pMMB207 failed to trigger stronger expression of bioactive caspase-1 and IL-1β in this work, indicating that NLRC4 could not directly detect PrgH to activate the inflammasome. None of the mutant strains C50336Δ*invC*, C50336Δ*spaN*, and C50336Δ*prgH* presented here decreased the mortality rate in the C57BL/6 mice model, in comparison with WT C50336. Whereas all of three gene deletion mutant strains were defective in inducing macrophage death in vitro, suggesting that the role of *Salmonella* SPI-1 T3SS in pathogenesis was different in vivo and in vitro. Besides, there may be other potential mechanisms for T3SS-defective SE mutants to induce activation of inflammasome in vivo.

Crucial for DNA methylation, Dam modulates bacterial gene expression through N-6 methylation of adenosine residues in GATC sites [39]. While a broad range of processes are modulated by Dam, such as DNA replication and mismatch repair, chromosome segregation, surface protein expression, and transcription of insertion elements, lack of Dam does not impair bacterial viability [22, 40]. In this study, a *dam* deletion mutant strain of SE was found to be defective in inducing activation of the inflammasome and pyroptosis, which was consistent with the findings of another study that found that IL-1β secretion is reduced in the absence of Dam compared with a WT ST strain [41]. Unlike the T3SS component deletion strains, *dam* mutations had an LD_50_ that was > 10^4^ higher than the WT parent strain in mice [22]. Similar results were found in this study. *Salmonella* Dam mutants were also attenuated for virulence in avian [42, 43], sheep [44], and bovine models [45, 46], which has been shown to confer cross-protective immunity to multiple *Salmonella* to prevent salmonellosis in animals and have been demonstrated as safe. Previous studies indicated that Dam is involved in the regulation of LPS synthesis, flagellar genes, fimbrial genes, and SPI-1 effector genes, and *prgH* also showed Dam-dependent regulation in both stationary-phase and static cultures [23], which seemed to be correlated with certain defects in the induction of pyroptosis and caspase-1 activation. Even then, *dam* mutants were able to persist in the liver, spleen, and lymph nodes of infected mice [47]. One likely explanation was that the reduced activation of the inflammasome induced by *dam* mutants resulted in *Salmonella* colonization in organs that could not be subjected to rapid and complete clearance, given that pyroptosis and secretion of IL-1β and IL-18 were an effective mechanism by which caspase-1 clears *Salmonella* [15]. A previous study showed that an ST strain that persistently expresses the flagellin protein FliC failed to evade NLRC4 detection and had been cleared efficiently [14].

Interestingly, the overexpression of Dam still prevented caspase-1 activation and cell death, which was consistent with the findings that a Dam-overproducing ST strain was highly attenuated in mice [22]. Therefore, we speculated that the deletion and overexpression of Dam may have a similar effect on infected macrophages, and confirmed by RNA-seq and quantitative real-time PCR (qRT-PCR). In infected macrophages, the number of overlapping DEGs and enriched pathways were very high between the C50336-VS-C50336Δ*dam* and C50336-VS-C50336::*dam* groups, suggesting that homeostasis of *dam* expression was essential for *Salmonella* virulence and the ability to induce inflammasome activation. The MAPK signaling pathway [48], TGF-β signaling pathway [49], TNF signaling pathway [50, 51], and FoxO signaling pathway [52] from overlapping enriched pathways were all reported to be related to the activation of inflammasome. The transcription regulator c-Fos plays an important role in regulating multiple signal pathways including MAPK and TNF [53]. A recent study reported that the c-Fos could bind to the promoter region of NLRP3 gene and positively regulate the inflammasome activation [54]. In this study, the expression levels of c-Fos induced by C50336Δ*dam* and C50336::*dam* were both significantly lower than that induced by WT in J774A.1 cells (Figs. 9 and 10), which could explain the defective activation of caspase-1 in both strains. Braf is an adaptor kinase in the MAPK pathway that transmits stimulatory signals of receptor binding growth factors [55]. Braf inhibitors were reported to upregulate the inflammasome activation and the production of IL-1β in dendritic cells [56]. Cullin1 (Cul1) was a key component of the Skp1-Cul1-F-box E3 ligase, which could bind and promote NLRP3 ubiquitination to repress systematic inflammasome activation [57]. Tgfbr1 and Pten were able to inhibit the expression of NLRP3, ASC, caspase-1, IL-1β, and IL-18 in a mouse squamous cell carcinoma of the head and neck model [58]. X-linked inhibitor of apoptosis protein (Xiap) was reported to restrict TNF- and RIP3-dependent cell death and inflammasome activation [50]. Besides, the IKKα (Chuk) was reported as a critical negative regulator of ASC-dependent inflammasomes, while the loss of IKKα kinase activity result in inflammasome hyperactivation [59]. In the FoxO pathway, the overexpression of Irs2 was able to inhibit the IL-1β promoter activity [60]. The anti-inflammatory factor Sirtuin 1 (Sirt1) is a conserved NAD-dependent protein deacetylase, which has been demonstrated to attenuate the activation of NLRP3 inflammasome [61]. In this study, we observed that both C50336Δ*dam* and C50336::*dam* could induce the high expression level of anti-inflammasome factors including Braf, Cul1, Tgfbr1, Pten, XIAP, Chuk, Irs2, and Sirt1 (Figs. 9 and 10), which could further explain why inflammasome could not be activated in the Dam deletion and overexpression strains.

A previous study demonstrated that apoptotic cell rates and NOS-2 and COX-2 expression were diminished in *dam* mutant strain-infected cells, coinciding with impaired activation of p38 and ERK MAPKs [62]. However, our result showed that the absence of Dam could not sufficiently inhibit the phosphorylation level of p38 and ERK1/2 for blocking the inflammasome activation. In addition, the Jnk pathway has been shown to regulate both at the priming stage and activation stage of NLRP3 inflammasome activation [63]. The phosphorylated Jnk is essentially required for ASC oligomerization, which is a critical step for NLRP3 inflammasome activation [64]. Recent studies reported that Jnk inhibitor could mediate Jnk inactivation, and further decrease the secretion of IL-1β and NLRP3 inflammasome activation [65]. In this study, both the deletion and overexpression of Dam decreased the phosphorylation level of Jnk, which could be related to the failure of activating inflammasome in SE infected macrophages, indicating that Jnk might become a target for SE in manipulating inflammasomes.

Moreover, the most surprising discovery in this study was that SE Dam specifically enhanced the activation of NLRP3 inflammasome independently via promoting Jnk phosphorylation in the absence of bacteria. Unlike the T6SS effector EvpP of *Edwardsiella tarda*, which could suppress activation of the NLRP3 inflammasome independently by preventing Jnk activation in macrophages [66]. Dam was not a direct activator and was unable to activate the NLRP3 inflammasome without the help of ATP, indicating that the function of Dam expressed in host cells independently may not be the same as that in bacteria. It is accepted that the extracellular stimuli could promote NLRP3 inflammasome activation by inducing various molecular mechanisms including lysosomal permeability, potassium efflux, and mitochondrial reactive oxygen species (ROS) production [67, 68]. The activation of Jnk pathway could enhance the activation of NLRP3 inflammasome through the production of MAPKs upstream signal ROS [65]. In this study, the expression level of Nr4a1 induced by C50336 was significantly higher than that induced by C50336Δ*dam*, while the overexpression of Nr4a1 could enhance the production of ROS [69]. Jnk-mediated caspase activation has been known to enhance the cleavage of Xiap and disrupt its function [70]. Pten inactivation was associated with increased activity of the Jnk pathway in human prostate cancer [71]. The expression level of Sirt1 protein was reported to be downregulated via Jnk activation-mediated protein degradation [72]. In this study, the expression level of Xiap, Pten, and Sirt1 induced by WT C50336 was significantly lower than that induced by *dam* deletion mutant strain, which is consistent with studies mentioned above. The increasing of intracellular Ca^2+^ was essential for activation of NLRP3 inflammasome [73]. The ASC oligomerization requires the release of Ca^2+^, which could activate the TAK1-Jnk pathway by an upstream kinase TAK1, and trigger the activation of NLRP3 inflammasome [63]. Base on the findings above, we speculated that Dam might have a potential function in regulating Ca^2+^ release via Jnk pathway, which could subsequently influence the inflammasome activation.

## Conclusions

In summary, we idenfied that Dam is a regulator of T3SS, which play an essential role in inducing inflammasome activation and pyroptosis during SE infection. We first described that the deletion and overexpression of Dam had a similar effect on inducing the macrophage death and secretion of inflammatory cytokines. The results of RNA-seq and qRT-PCR suggested that both C50336Δ*dam* and C50336::*dam* were defective in inhibiting the expression of several anti-inflammasome factors (such as Braf, Cul1, Xiap, Pten, and Sirt1) compared to WT. In addition, both the absence and overexpression of Dam blocked the activation of Jnk pathway during SE infection. To our knowledge, it was the frst demonstration that Dam could enhance the activation of the NLRP3 inflammasome independently via promoting Jnk phosphorylation in the absence of bacteria. Further research will focus on exploring more specific molecular mechanism of Dam regulates the activation of inflammasome and the effect of different expression levels of Dam on inflammasome activation. Collectively, our results provides a novel role of Dam in modulating inflammasome response and offers a new insights of the complex interplay between Dam and the inflammasome.

## Methods

### Bacterial strains

The wild-type SE strain C50336 was obtained from the National Institute for the Control of Pharmaceutical and Biological Products (Beijing, China). *Escherichia coli* X7213 *λpir* is stored in our laboratory. The bacterial strains and plasmids used in this study are listed in Table 1. The gene deletion mutant strains were constructed by double exchange of homologous recombination as described previously [77]. The upstream and downstream fragments of target genes were amplified using PCR by employing the primers listed in Table 2. The PCR products were purified using the TaKaRa MiniBEST Agarose Gel DNA Extraction Kit Ver 4.0 (TaKaRa Biotechnology Co. Ltd., Dalian, China). The pDM4 plasmid was digested by restriction endonucleases *Sal* I and *Sac* I (TaKaRa). The purified plasmid and upstream and downstream fragments were fused using the ClonExpress MultiS One Step Cloning Kit (Vazyme Biotechnology Co. Ltd., Nanjing, China). The recombinant plasmids were transferred into X7213 *λpir* cells and sequenced. Single-crossover mutants were obtained by conjugal transfer of the recombinant suicide plasmids into C50336. Deletion mutants were screened on 15% sucrose Luria-Bertani (LB) plates. Construction of gene complementation strains and overexpression strains were performed as described previously [24]. Target genes were cloned into the low copy number plasmid pMMB207 by employing the ClonExpress II One Step Cloning Kit (Vazyme). The recombinant plasmids were mated from X7213 *λpir* into mutant or WT C50336 by conjugation. Chloramphenicol resistant transconjugants were selected, and the presence of the plasmids was confirmed by PCR analysis with flanking primers and sequencing.

**Table 1:** Bacterial strains and plasmids used in this study

Strain or plasmid	Relevant characteristics	Reference
***Escherichia coli***		
X7213 λ*pir*	Host for π requiring plasmids, conjugal donor	Labortaory collection
X7213 λ*pir*-pSC189	X7213 λ*pir* with pSC189, Km^r^, Cm^r^	This study
***Salmonella*Enteritidis**		
C50336	Wild type	Obtained from Chinese National Institute for the Control of Pharmaceutical and Biological
C50336∆*dam*	C50336, In-frame deletion in *dam*	This study
C50336∆*invC*	C50336, In-frame deletion in *invC*	This study
C50336∆*hilD*	C50336, In-frame deletion in *hilD*	This study
C50336∆*prgH*	C50336, In-frame deletion in *prgH*	This study
C50336∆*spaN*	C50336, In-frame deletion in *spaN*	This study
C50336∆*dam*::*dam*	C50336∆*dam* with pMMB207 expressing the *dam* gene,Cm^r^	This study
C50336∆ *invC*::*invC*	C50336∆ *invC* with pMMB207 expressing the *invC* gene,Cm^r^	This study
C50336∆*hilD*::*hilD*	C50336∆ *invC* with pMMB207 expressing the *hilD* gene,Cm^r^	This study
C50336∆ *prgH*::*prgH*	C50336∆ *prgH* with pMMB207 expressing the *prgH* gene,Cm^r^	This study
C50336∆ *spaN*::*spaN*	C50336∆*dam* with pMMB207 expressing the *dam* gene,Cm^r^	This study
C50336-pMMB207	C50336 with pMMB207,Cm^r^	This study
C50336::*dam*	C50336 with pMMB207 expressing the *dam* gene,Cm^r^	This study
C50336::*prgH*	C50336 with pMMB207 expressing the *prgH* gene,Cm^r^	This study
C50336∆*dam*-pMMB207	C50336∆*dam* with pMMB207,Cm^r^	This study
C50336∆*prgH*-pMMB207	C50336∆*prgH* with pMMB207,Cm^r^	This study
C50336-pCX340	C50336 with pCX340, Tet^r^	This study
C50336-pCX340-*dam*	C50336 with pCX340 expressing the *dam* gene, Tet^r^	This study
C50336-pCX340-*invC*	C50336 with pCX340 expressing the *invC* gene, Tet^r^	This study
C50336-pCX340-*prgH*	C50336 with pCX340 expressing the *prgH* gene, Tet^r^	This study
C50336-pCX340-*spaN*	C50336 with pCX340 expressing the *dam* gene, Tet^r^	This study
**Plasmids**		
pSC189	Transposon delivery vector, R6K, Km^r^, Cm^r^	[74]
pDM4	Suicide vector, *pir* dependent, R6K, *SacBR*, Cm^r^	[75]
pMMB207	IncQ lacI^q^ Δ*bla* P_tac-lac_*lacZa*, Cm^r^	[24]
pMMB207-*dam*	pMMB207 derivative containing *dam*, Cm^r^	This study
pMMB207-*invC*	pMMB207 derivative containing *invC*, Cm^r^	This study
pMMB207-*hilD*	pMMB207 derivative containing *hilD*, Cm^r^	This study
pMMB207-*prgH*	pMMB207 derivative containing *prgH*, Cm^r^	This study
pMMB207-*spaN*	pMMB207 derivative containing *spaN*, Cm^r^	This study
pCX340	pBR322 derivative, cloning vector used to fuse effectors to TEM-1-β-lactamase, Tet^r^	[76]
pCX340-*dam*	pCX340 derivative containing *dam*	This study
pCX340-*invC*	pCX340 derivative containing *invC*	This study
pCX340-*prgH*	pCX340 derivative containing *prgH*	This study
pCX340-*spaN*	pCX340 derivative containing *spaN*	This study

The antibiotics as follows: kanamycin (Km^r^), 100 μg/mL; chloramphenicol (Cm^r^), 25 μg/mL; tetracycline (Tet^r^), 12.5 μg/mL

**Table 2:** Primers used in this study

Primer name	Primer sequence (5′ to 3′)	Target
AB1	GGCCACGCGTCGACTAGTACNNNNNNNNNNACGCC	For transposon insertion sequencing
AB2	GGCCACGCGTCGACTAGTACNNNNNNNNNNCCTGG	For transposon insertion sequencing
AB3	GGCCACGCGTCGACTAGTACNNNNNNNNNNCCTCG	For transposon insertion sequencing
ABS	GGCCACGCGTCGACTAGTAC	For transposon insertion sequencing
SP1	GCTGACCGCTTCCTCGTGCTTTACG	For transposon insertion sequencing
SP2	CATCGCCTTCTATCGCCTTCTTGAC	For transposon insertion sequencing
pSC189-seq	CGCGAAGTTCCTATTCCGAAGTTCC	For transposon insertion sequencing
pDM4-F	GGTGCTCCAGTGGCTTCTGTTTCTA	For deletion mutants
pDM4*-R*	CAGCAACTTAAATAGCCTCTAAT	For deletion mutants
*dam*-up-F	GAGCGGATAACAATTTGTGGAATCCCGGGAGACGCCGAAAGCGACCACCACGACG	For *dam* deletion mutant
*dam*-up-R	TTGAGAATTACATGCTGACTAACTAATTACACCTT	For *dam* deletion mutant
*dam*-down-F	AGTCAGCATGTAATTCTCAAGGAGAAGCGGATGAA	For *dam* deletion mutant
*dam*-down-R	AGCGGAGTGTATATCAAGCTTATCGATACCCGATCTCGCCGATATTGTTCACCTT	For *dam* deletion mutant
*dam*-in-F	CGAGTGCCTTGTCGAACCTTTTGTG	For *dam* deletion mutant
*dam-*in-R	ACAGAGCCAGCAGTTCGTCCACCTT	For *dam* deletion mutant
*dam*-out-F	AACCACGACTGCGGAACCGAAGAAA	For *dam* deletion mutant
*dam*-out-R	TCGGGTTTATCGAAAATTGCCGACC	For *dam* deletion mutant
*invC*-up-F	GAGCGGATAACAATTTGTGGAATCCCGGGAGTCCTCCTTACGTCTGTCGATGTCC	For *invC* deletion mutant
*invC*-up-R	GCGAATGCATTCATCTCATTAGCGACCGACTAAAA	For *invC* deletion mutant
*invC*-down-F	AATGAGATGAATGCATTCGCTGACCAGAATTAAAG	For *invC* deletion mutant
*invC*-down-R	AGCGGAGTGTATATCAAGCTTATCGATACCAATGCTTCTGATAAACCGCCAACCT	For *invC* deletion mutant
*invC*-in-F	GGGAACGCACCGTGTTGAGCCTTAT	For *invC* deletion mutant
*invC-*in-R	CCAGGACGATATTCTCCCAAGTCAA	For *invC* deletion mutant
*invC*-out-F	CAGTACCTTCCTCAGCCTTGACCCG	For *invC* deletion mutant
*invC*-out-R	GCATTACGAAAGCATCGCCATAGTC	For *invC* deletion mutant
*hilD*-up-F	GAGCGGATAACAATTTGTGGAATCCCGGGAGCTCATGGAGTATAATTTCGGTCGT	For *hilD* deletion mutant
*hilD*-up-R	AAAAATGTTACATATTATCCCTTTGTTGATGTTAT	For *hilD* deletion mutant
*hilD*-down-F	GGATAATATGTAACATTTTTTGTATCTGTCACTTA	For *hilD* deletion mutant
*hilD*-down-R	AGCGGAGTGTATATCAAGCTTATCGATACCTGAAAGACTGTTTTTAATGGTGCGC	For *hilD* deletion mutant
*hilD*-in-F	GTCAGACTCAGCAGGTTACCATCAA	For *hilD* deletion mutant
*hilD-*in-R	CATTATGGTTGCCTATGCGTAAAAG	For *hilD* deletion mutant
*hilD*-out-F	TTCACCGACCTGTATTGGCGTATTT	For *hilD* deletion mutant
*hilD*-out-R	TTTTGGGGTGTAAATGCTGCTTATT	For *hilD* deletion mutant
*prgH*-up-F	GAGCGGATAACAATTTGTGGAATCCCGGGACACCAACATCCCAGGTTCGTCACAG	For *prgH* deletion mutant
*prgH*-up-R	CGTTAAATTACATATATACTGTTAGCGATGTCTGT	For *prgH* deletion mutant
*prgH*-down-F	AGTATATATGTAATTTAACGTAAATAAGGAAGTCA	For *prgH* deletion mutant
*prgH*-down-R	AGCGGAGTGTATATCAAGCTTATCGATACCTTCAACAGCCCCGACTCCTTTACGA	For *prgH* deletion mutant
*prgH*-in-F	GTTTGCTGCTCGTTTGGGATAAGTG	For *prgH* deletion mutant
*prgH-*in-R	GGCAAGGGTCATTACCAGCAGAAAG	For *prgH* deletion mutant
*prgH*-out-F	GAACGGCTGTGAGTTTCCATTGCTG	For *prgH* deletion mutant
*prgH*-out-R	GACGGGCTCTGAGTATTTCTACATC	For *prgH* deletion mutant
*spaN*-up-F	GAGCGGATAACAATTTGTGGAATCCCGGGAATTGACTTGGGAGAATATCGTCCTG	For *spaN* deletion mutant
*spaN*-up-R	AATGACATCACATTAAATTATCTCCTCTGACTCGG	For *spaN* deletion mutant
*spaN*-down-F	TAATTTAATGTGATGTCATTGCGTGTGAGACAGAT	For *spaN* deletion mutant
*spaN*-down-R	AGCGGAGTGTATATCAAGCTTATCGATACCCGCACGGGAAGTACGAATCAGGAGT	For *spaN* deletion mutant
*spaN*-in-F	CGACTATGGCGATGCTTTCGTAATG	For *spaN* deletion mutant
*spaN-*in-R	CAAACGATGTTCAACCTGCGTATTT	For *spaN* deletion mutant
*spaN*-out-F	AAAGCGTAAGCCGCGTTTTTGGACA	For *spaN* deletion mutant
*spaN*-out-R	CAATTGATTCAAGCCAGGCAGAGTT	For *spaN* deletion mutant
pMMB207-F	CTCCCGTTCTGGATAATGTT	For complemented mutants
pMMB207-R	GGCGTTTCACTTCTGAGTTCG	For complemented mutants
pMMB207*-dam*-F	AGCTCGGTACCCGGGGATCCTCTAGCTAAAGGAAGACGTTATGAAAAAAAATCGCGCTTTTTTGA	For complemented mutant of ∆*dam*
pMMB207*-dam*-R	TCTCATCCGCCAAAACAGCCAAGCTTTATTTTCTTGCAGGCGTTGCGACT	For complemented mutant of ∆*dam*
pMMB207*-invC*-F	AGCTCGGTACCCGGGGATCCTCTAGCTAAAGGAAGACGTTATGAAAACACCTCGTTTACTGCAAT	For complemented mutant of ∆ *invC*
pMMB207*-invC*-R	TCTCATCCGCCAAAACAGCCAAGCTTTAATTCTGGTCAGCGAATGCATTC	For complemented mutant of ∆ *invC*
pMMB207*-prgH*-F	AGCTCGGTACCCGGGGATCCTCTAGCTAAAGGAAGACGTTATGGAAACATCAAAAGAGAAGACGA	For complemented mutant of ∆*prgH*
pMMB207*-prgH*-R	TCTCATCCGCCAAAACAGCCAAGCTTTAAAGTGGGCTTGGGAAATACCAA	For complemented mutant of ∆*prgH*
pMMB207*-spaN*-F	AGCTCGGTACCCGGGGATCCTCTAGCTAAAGGAAGACGTTATGGGCGATGTGTCAGCTGTCAGTT	For complemented mutant of ∆*spaN*
pMMB207*-spaN*-R	TCTCATCCGCCAAAACAGCCAAGCTTCAGGCGTCATCCTCCTCGCCAGAT	For complemented mutant of ∆*spaN*
pCX340-F	AGACAATCTGTGTGGGCACTCGACC	For β-lactamase TEM-1 fusion plasmid
pCX340*-*R	TTCTGAGAATAGTGTATGCGGCGAC	For β-lactamase TEM-1 fusion plasmid
pCX340*-dam*-F	AAGGAGGAATAACATATGATGAAAAAAAATCGCGCTTTTTTGA	For β-lactamase TEM-1 fusion plasmid
pCX340*-dam*-R	GTGCGAATTCTCCGCGGAGGTACCTTTTCTTGCAGGCGTTGCGACT	For β-lactamase TEM-1 fusion plasmid
pCX340*-invC*-F	AAGGAGGAATAACATATGATGAAAACACCTCGTTTACTGCAAT	For β-lactamase TEM-1 fusion plasmid
pCX340*-invC*-R	GTGCGAATTCTCCGCGGAGGTACCATTCTGGTCAGCGAATGCATTC	For β-lactamase TEM-1 fusion plasmid
pCX340*-prgH*-F	AAGGAGGAATAACATATGATGGAAACATCAAAAGAGAAGACGA	For β-lactamase TEM-1 fusion plasmid
pCX340*-prgH*-R	GTGCGAATTCTCCGCGGAGGTACCAAGTGGGCTTGGGAAATACCAA	For β-lactamase TEM-1 fusion plasmid
pCX340*-spaN*-F	AAGGAGGAATAACATATGATGGGCGATGTGTCAGCTGTCAGTT	For β-lactamase TEM-1 fusion plasmid
pCX340*-spaN*-R	GTGCGAATTCTCCGCGGAGGTACCGGCGTCATCCTCCTCGCCAGAT	For β-lactamase TEM-1 fusion plasmid

### Construction of transposon mutant library and transposon insertion sequencing

Random transposition of TnpSC189 into the C50336 chromosome was achieved by using the delivery plasmid pSC189 [74]. For conjugation, the X7213 *λpir* donor strain bearing the plasmid pSC189 was cultivated in LB broth containing 50 μg/mL DAP (diaminopimelic acid) and 100 μg/mL kanamycin, while recipient strain C50336 was cultured in ordinary LB broth and grown until mid-logarithmic phase (OD_600_ = 0.6). Equal volumes (100 μL) of bacterial suspensions were mixed after two washes with fresh LB medium. The mixed suspension was plated on nitrocellulose filters placed on the surface of LB agar plates supplemented with DAP (50 μg/mL) and incubated for 12 h at 37 °C. The conjugation mixtures were then diluted with sterile phosphate-buffered saline (PBS) and plated on LB agar supplemented with kanamycin (100 μg/mL).

Two-round semi-arbitrary PCR was used to determine the location of the transposon insertion [78]. PrimeSTAR® Max DNA Polymerase (TaKaRa) was employed in a 10-μL reaction volume consisting of 2 × PrimeSTAR Max Premix (5 μL), 0.25 μM of SP1 primer, 0.1 μM of AB1, AB2, and AB3 primers (Table 2), and 1 μL of an overnight LB-grown culture as the source of DNA. The first-round reaction conditions were as follows: 1 cycle of 95 °C for 5 min, 6 cycles of 95 °C for 30 s, 42–36 °C for 30 s (1 °C reduction per cycle), and 72 °C for 3 min, then 26 cycles of 95 °C for 30 s, 58 °C for 30 s, and 72 °C for 3 min, followed by 1 cycle of 72 °C for 10 min. The second-round reaction was performed in a total volume of 30 μL containing 15 μL of 2 × PrimeSTAR Max Premix, 0.25 μM of SP2 and ABS primers (Table 2), with 2 μL of the first-round reaction product as the source of DNA. The second-round PCR amplifications were performed as follows: 1 cycle of 95 °C for 5 min, 30 cycles of 95 °C for 30 s, 64 °C for 30 s, and 72 °C for 3 min, followed by 1 cycle of 72 °C for 10 min. The second-round PCR products were sequenced using the pSC189-seq primer (Table 2) and the sequences were compared with the GenBank DNA sequence database using the BLASTX program.

### Mice and cell culture

Specific pathogen-free (SPF) female C57BL/6 mice (age, 6–8 weeks; body weight, 20 ± 2 g) were obtained from the Comparative Medical Center of Yangzhou University (Yangzhou, China). All mice were bred under specific pathogen-free conditions, in the mouse isolators (Suzhou monkey animal experiment equipment Technology Co. Ltd., Suzhou, China). Five mice were raised in a cage, fed with pathogen-free diet and water. All animal experiments were approved by the Animal Welfare and Ethics Committees of Yangzhou University and complied with the guidelines of the Institutional Administrative Committee and Ethics Committee of Laboratory Animals (IACUC license number: YZUDWLL-201811-001). All animals were subjected to a clinical examination to assess their physical appearance and the normality of their behavior, and those presenting signs of disease were removed. All animals were humanely handled. For bone marrow preparations animals were sacrificed by cervical dislocation after anesthesia by intraperitoneal injection of 20% urethane (ethyl carbamate) solution. Animals used for the evaluation of bacterial virulence in this study were euthanized by CO_2_ delivery from a compressed CO_2_ gas cylinder directly in the mouse cages to reduce stress. Euthanasia was accomplished by slow exposure to increasing levels of CO_2_, replacing approximately 15–30% of the cage volume per minute, outside the mouse cages. Flow was kept for 2 min and the animals were maintained in the same cage for an additional 5 min.

BMDMs are primary macrophages obtained from C57BL/6 mouse bone marrow of the tibia and femur as described previously [79]. Three mice were uesd for BMDM preparation in each individual experiment, three independent experiments were performed. Bone marrow cells were cultured at 37 °C with 5% CO_2_ in Dulbecco’s modified Eagle’s medium (DMEM, Gibco, Grand Island, NY, USA) supplemented with 10% (v/v) fetal bovine serum (FBS, Gibco), 100 U/mL penicillin, 100 μg/mL streptomycin (Gibco), and 25 ng/mL macrophage colony-stimulating factor (M-CSF, PeproTech, Rocky Hill, NJ, USA). Fresh medium was added on day 3. Differentiated BMDMs were collected for experiments on day 6. J774A.1 and HeLa cells were were purchased from American Tissue Culture Collection (ATCC, Manassas, VA, USA) and cultured in complete DMEM containing 10% FBS, penicillin (100 U/mL), and streptomycin (100 μg/mL). Cell viability and number were determined by trypan blue exclusion assays.

### *Salmonella* infection

Each single SE colony was cultured in LB medium, followed by overnight incubation at 37 °C with shaking at 180 rpm. Overnight cultures were diluted 1:100 and cultured for another 3 h to an OD_600_ of 0.7 to induce SPI-1 expression [80]. J774A.1 cells or BMDMs were seeded into cell culture plates the day before infection. For inflammasome activation, the cells were washed with Dulbecco’s PBS (DPBS, Gibco) and pre-treated with 1 μg/mL LPS (Sigma-Aldrich, St. Louis, MO, USA) diluted in Opti-MEM (Gibco) for 5 h. Bacteria strains were washed twice with sterile PBS, added to the cells at an MOI of 20:1, and spun onto the cells at 1000 rpm for 10 min. Cells were incubated at 37 °C for 1 h, subsequently washed twice, and incubated for an additional 3 h with Opti-MEM containing 50 μg/mL gentamicin.

In total, 70 female C57BL/6 mice (6–8 weeks old) were randomly assigned to seven groups (*n* = 10). Each group was intraperitoneally infected with 100 μL of WT C50336 or gene deletion mutant strains diluted in PBS at a dose of 1 × 10^5^ CFU per mouse. The control mice received 100 μL of PBS via the same route. All mice deaths were recorded over 14 dpc.

### Cytotoxicity assays and cytokine measurements

J774A.1 cells or BMDMs were seeded at a concentration of 1 × 10^5^ cells per well in 48-well plates. The cells were infected with SE strains as described above, and supernatants were harvested at 4 h after infection and centrifuged at 2000 rpm for 5 min to remove cell debris. LDH release was quantified using the LDH Cytotoxicity Assay Kit (Beyotime Biotechnology Co. Ltd., Haimen, China) according to the manufacturer’s instructions. Quantitative determination of pro-inflammatory cytokines IL-1β and IL-6 in supernatants were performed through an enzyme-linked immunosorbent assay (ELISA) by employing Mouse IL-1 beta/IL-1F2 DuoSet ELISA and Mouse IL-6 DuoSet ELISA (R&D Systems, Minneapolis, MN, USA) according to the manufacturer’s manual.

### Immunoblotting and antibodies

J774A.1 cells or BMDMs were seeded into 12-well plates at a density of 5 × 10^5^ cells per well and infected with bacteria as described above. After harvesting the supernatants, the remaining cells were directly lysed with 300 μL cell lysis buffer per well for western and IP (Beyotime). For each single well, the supernatant and lysate were pooled separately. The mixtures were centrifuged at 2000 rpm for 5 min to remove cell debris. An equal volume of methanol and a 0.25 volume of chloroform were added, and the solution was vortexed vigorously for 30 s. The samples were centrifuged at 12000 rpm for 5 min. The upper aqueous phase was removed, and an equal volume of methanol was added to each sample. The mixtures were centrifuged at 12000 rpm for 5 min after vortexing for 10 s. The protein pellets were dried at 55 °C for 5–10 min, resuspended with 40 μL of 1 × SDS-PAGE Sample Loading Buffer (Beyotime), and boiled for 10 min at 95 °C. The samples were loaded onto 15% Tris-glycine gels. The proteins were then transferred from the gels onto nitrocellulose membranes, which were blocked with blocking buffer (3% nonfat dry milk in PBS) for 2 h at room temperature. The membranes were subsequently incubated on a rotator overnight at 4 °C with a primary antibody (diluted 1:1000 in blocking buffer). After 5 washes with PBST (0.05% Tween 20 in PBS), the membranes were incubated at room temperature for 1.5 h with a secondary antibody diluted 1:5000 in blocking buffer. Images of antibody reactions with an ECL chemiluminescence substrate (Thermo Scientific, Waltham, MA, USA) were acquired using an Amersham Imager 600 Imaging System (GE Healthcare Life Sciences, Pittsburgh, PA, USA).

The primary antibodies used in this study were as follows: anti-caspase-1 p20 antibody (AG-20B-0042, AdipoGen, San Diego, CA, USA), anti-NLRP3/NALP3 antibody (AG-20B-0014, AdipoGen), anti-NLRC4 antibody (ab201792, Abcam, Cambridge, UK), anti-ASC/TMS1 antibody (67824S, Cell Signaling Technology, Danvers, MA, USA), anti-IL-1β antibody (12507S, Cell Signaling Technology), anti-β-actin antibody (A5441, Sigma-Aldrich), anti-Phospho-SAPK/Jnk antibody (4671S, Cell Signaling Technology), anti-Phospho-p44/42 MAPK (ERK1/2) antibody (4377S, Cell Signaling Technology), and anti-Phospho-p38 MAPK antibody (9215S, Cell Signaling Technology). The secondary antibodies were goat anti-mouse IgG-HRP (401,215, Sigma-Aldrich) and goat anti-rabbit IgG-HRP (BS13278, Bioworld Technology, Bloomington, MN, USA).

### Protein translocation assays

Plasmid pCX340 was used for a TEM-1 β-lactamase fusion protein FRET assay as described previously [76]. The target genes were amplified from the genome of wild-type C50336 and then ligated into the *Nde* I and *Kpn* I restriction sites in the multiple cloning region of pCX340 using the ClonExpress II One Step Cloning Kit (Vazyme). All recombinant plasmids were transferred into C50336 by electroporation. Overnight bacterial cultures were diluted to 1:100 in LB broth supplemented with 12.5 μg/mL tetracycline for 3 h and induced with 1 mM IPTG for another 3 h.

HeLa cells were trypsinized and seeded in black, clear-bottomed 24-well plates (Cellvis, Mountain View, CA, USA) at 2 × 10^5^ cells per well. Bacterial strains bearing plasmid pCX340 were washed twice with DMEM, added to the cells at an MOI of 100:1, and spun onto the cells at 1000 rpm for 10 min. The cells were incubated for 3 h, subsequently washed 4 times, and incubated for an additional 4 h with DMEM. The cells were washed with DMEM and covered with 300 μL DMEM plus 60 μL of 6 × CCF2/AM solution freshly prepared with the CCF2/AM loading kit (CCF2/AM final concentration,1 μM; Invitrogen, Carlsbad, CA, USA). The plates were incubated for 2 h at room temperature, and images were acquired using a Leica confocal microscope (Leica Microsystems, Wetzlar, Germany). The appropriate filters were selected to allow for excitation of coumarin (~ 410 nm) and detection of blue coumarin (~ 450 nm) and green fluorescein (~ 520 nm) emissions, enabling the simultaneous observation of green fluorescence emitted by the CCF2 substrate and blue fluorescence emitted by the cleaved CCF2 product. Translocation of β-lactamase fusions into CCF2-loaded cells was determined by counting the number of blue fluorescent cells in the images. Cells that did not exhibit secretion of TEM fusions appeared green. For a particular cell well from three separate experiments, six pictures were taken, and an average of 1200–2000 cells were counted. Each picture was considered an independent observation and used to calculate the percentage of blue fluorescent cells.

### Lentivirus infection assays

Lentiviral vectors pGLV5-*dam* (EF-1aF/GFP&Puro) and pGLV5-*dam*-His (EF-1aF/GFP&Puro) for the Dam gene encoding a GFP sequence were designed, constructed, amplified, and purified by GenePharma (Shanghai, China). The packaging, collection, and titer determination of the lentivirus were also conducted by GenePharma. In general, 293 T producer cells were transfected with optimized packaging plasmids (pGag/Pol, pRev, and pVSV-G) along with lentiviral vectors expressing Dam or Dam-His constructs by lipofectamine. Six hours post-transfection, the transfection mix was replaced with fresh DMEM containing 10% FBS. The virus-containing supernatant was harvested 72 h post-transfection, centrifuged at 4 °C and 4000 rpm for 4 min, filtered through a 0.45 μm filter (Millipore, Burlington, MA, USA), and centrifuged at 4 °C and 20,000 rpm for 2 h. The lentivirus particles were titrated by adding serial dilutions to fresh 293 T cells and assessed using GFP expression after 72 h. The viral titers of approximately 1 × 10^9^ infectious units/mL were obtained and stored at − 80 °C before infection. The negative control lentivirus Lv-NC (10^9^ TU/mL) was also provided by GenePharma.

J774A.1 cells were seeded into 12-well plates at a density of 1 × 10^5^ cells per well. A 100 μL volume of lentivirus was mixed with 400 μL of complete DMEM, and polybrene (GenePharma) was added to a final concentration of 5 μg/mL. The cells were incubated at 37 °C for 24 h, the medium was removed, and the cells were cultured in 500 μL of complete DMEM for another 48 h. The efficiency of lentivirus infection was evaluated by detection of GFP fluorescence. The cells were pre-treated with LPS as described above and stimulated with 1.25 mM ATP (Sigma) diluted in Opti-MEM (Gibco) for 1 h. Then supernatants and cell lysates were collected for immunoblotting or ELISA as described above.

### RNA sequencing

Overnight cultures of the wild-type strain C50336, gene deletion mutant strain C50336Δ*dam*, and overexpression strain C50336::*dam* were diluted to 1:100 in LB broth and cultured to logarithmic phase (OD_600_ = 0.7). J774A.1 cells were seeded at a density of 5 × 10^6^ cells per 10-cm dish. The cells were pre-treated with LPS and infected with SE strains as described above. Each group comprised three independent replicates. The cells were then collected with 1 mL Trizol (Ambion, Carlsbad, CA, USA) and sent to GENEWIZ (Suzhou, China) for analysis. The total RNA was isolated, purified, and quantified by GENEWIZ. The eukaryotic transcriptome libraries were pooled and sequenced on an Illumina platform (Illumina, San Diego, CA, USA). The subsequent procedures and statistical analyses were conducted by GENEWIZ as previously described [81]. All sequences were deposited at NCBI Sequence Read Archive (SRA) with accession number SRP253144.

### qRT-PCR analysis

J774A.1 cells were seeded into 12-well plates at a density of 5 × 10^5^ cells per well and infected with WT C50336, *dam* deletion mutant, complementation, and overexpression strains as described above. After infection for 4 h, cells were harvested and total RNA was extracted using E.Z.N.A.™ Total RNA Kit I (Omega Bio-tek, Norcross, GA, USA) by following the manufacturer’s instructions. RNase-free DNase I (TaKaRa) was used to remove contaminating DNA in purified total RNA, in accordance with the manufacturer’s instructions. RNA of the infected cells were quantified using One drop™ spectrophotometer (Wins Technology Co. Ltd., Nanjing, China). PrimeScript RT Reagent Kit (TaKaRa) was used to reverse transcribe total RNA into cDNA. Reaction was performed in a total volume of 20 μL, containing 1 μg of total RNA, 1 μL of PrimeScript RT Enzyme Mix I, 4 μL of RT Primer Mix, 4 μL of 5 × PrimeScript Buffer 2. The mixture was incubated at 37 °C for 15 min, followed by incubation at 85 °C for 5 s, subsequently stored at − 20 °C until further use.

The Applied Biosystems QuantStudio 6 Flex Real-Time PCR System (Applied Biosystems, Foster City, CA, USA) was used to measure the mRNA expression levels of c-Fos, Braf, Rasa1, Sos2, Nr4a1, Elk4, Mef2c, Cul1, Tgfbr1, Rps6kb1, Rbl1, Id1, Id3, Xiap, Bcl3, Chuk, Dnm1l, Akt3, Itch, Pik3ca, Pik3r1, Pten, Irs2, Sirt1, Klf2, Sgk3, Pdpk1, Mdm2, and Rbl2 as well as of the internal control gene, mouse GAPDH. Primers were designed using Primer Express software v3.0 (Applied Biosystems, Carlsbad, CA, USA) and were listed in Table 3. The qRT-PCR reaction was performed in a total volume of 20 μL, containing 200 ng of cDNA, 10 μL of 2 × SYBR Green Master Mix (TaKaRa), 0.6 μL each of 10 μM forward and reverse primers, and 6.8 μL RNase-free water. The comparative threshold cycle (2^–ΔΔC(T)^ method) was used to calculate relative concentrations. All qRT-PCR reactions were performed in triplicates.

**Table 3:** Primers used in qRT-PCR

Primer name	Primer sequence (5′ to 3′)	Target
c-Fos-F	CTCCCGTGGTCACCTGTACT	Detection of mRNA
c-Fos-R	TTGCCTTCTCTGACTGCTCA	Detection of mRNA
Braf-F	GTAGCGCCTGTTCAGTCCTC	Detection of mRNA
Braf-R	GAGCAGCCTGAGTGGTTAGG	Detection of mRNA
Rasa1-F	CGGGGTCCTTTGTACTTTCA	Detection of mRNA
Rasa1-R	TGGTGGTGCAACTGGATAGA	Detection of mRNA
Sos2-F	CAGTCCTCTTGCCACACTCA	Detection of mRNA
Sos2-R	GTGGAATAGCAGGAGGGTCA	Detection of mRNA
Nr4a1-F	CTTGAGTTCGGCAAGCCTAC	Detection of mRNA
Nr4a1-R	CGAGGATGAGGAAGAAGACG	Detection of mRNA
Elk4-F	AGCTTTGCCAGAAAAGGACA	Detection of mRNA
Elk4-R	TGGTGTAAGAGACGCTGTCG	Detection of mRNA
Mef2c-F	ACGCCTGTCACCTAACATCC	Detection of mRNA
Mef2c-R	AGCTCTCAAACGCCACACTT	Detection of mRNA
Cul1-F	GAATTGGGGCTGAATGAAGA	Detection of mRNA
Cul1-R	AACTCTCCGCTGTTCCTCAA	Detection of mRNA
Tgfbr1-F	GGCGAAGGCATTACAGTGTT	Detection of mRNA
Tgfbr1-R	TGCACATACAAATGGCCTGT	Detection of mRNA
Rps6kb1-F	GCTGTGGATTGGTGGAGTTT	Detection of mRNA
Rps6kb1-R	GCTTGGACTTCTCCAGCATC	Detection of mRNA
Sp1-F	TGCAGCAGAATTGAGTCACC	Detection of mRNA
Sp1-R	CACAACATACTGCCCACCAG	Detection of mRNA
Rbl1-F	AAACCTTGCACCACAAGTACG	Detection of mRNA
Rbl1-R	GCACAGGAGACATTTGATCATT	Detection of mRNA
Id1-F	CCAGTGGGTAGAGGGTTTGA	Detection of mRNA
Id1-R	AGAAATCCGAGAAGCACGAA	Detection of mRNA
Id3-F	ACTCAGCTTAGCCAGGTGGA	Detection of mRNA
Id3-R	GTCAGTGGCAAAAGCTCCTC	Detection of mRNA
Xiap-F	TTGGAACATGGACATCCTCA	Detection of mRNA
Xiap-R	TGCCCCTTCTCATCCAATAG	Detection of mRNA
Bcl3-F	TTACTCTACCCCGACGATGG	Detection of mRNA
Bcl3-R	CCAAGCTTGAAAAGGCTGAG	Detection of mRNA
Chuk-F	TGGAGCCTACGAAGCTGTTT	Detection of mRNA
Chuk-R	CCCTCATTAGTTGCGGTGTT	Detection of mRNA
Dnm1l-F	ACCCGGAGACCTCTCATTCT	Detection of mRNA
Dnm1l-R	GGCGAGAAAACCTTGAGATG	Detection of mRNA
Akt3-F	GAAACTGGCCACTTCTGCTC	Detection of mRNA
Akt3-R	ACTGAGGTGTGGTGGAGACC	Detection of mRNA
Itch-F	CATGTGGTTTTGGCAGTTTG	Detection of mRNA
Itch-R	TTGTAAGGTGGGAGGTCCAG	Detection of mRNA
Pik3ca-F	ACTGTTCAGAGAGGCCAGGA	Detection of mRNA
Pik3ca-R	CGGTTGCCTACTGGTTCAAT	Detection of mRNA
Pik3r1-F	GCGTGACATGTAGGCTCTCA	Detection of mRNA
Pik3r1-R	CAGTTTCCTTGGCTTTGCTC	Detection of mRNA
Pten-F	CATAACGATGGCTGTGGTTG	Detection of mRNA
Pten-R	CGGGGTAAGGCTGTTTTACA	Detection of mRNA
Irs2-F	GTAGTTCAGGTCGCCTCTGC	Detection of mRNA
Irs2-R	CAGCTATTGGGACCACCACT	Detection of mRNA
Sirt1-F	AGTTCCAGCCGTCTCTGTGT	Detection of mRNA
Sirt1-R	CTCCACGAACAGCTTCACAA	Detection of mRNA
Klf2-F	GCCTGTGGGTTCGCTATAAA	Detection of mRNA
Klf2-R	AAGGAATGGTCAGCCACATC	Detection of mRNA
Sgk3-F	ATCCAGATGTCCGAGCATTC	Detection of mRNA
Sgk3-R	GAAGAACCTTGCCAAAGCTG	Detection of mRNA
Pdpk1-F	GGTCCAGTGGATAAGCGAAA	Detection of mRNA
Pdpk1-R	TTTCTGCACCACTTGTGAGC	Detection of mRNA
Mdm2-F	TGCAAGCACCTCACAGATTC	Detection of mRNA
Mdm2-R	ACACAATGTGCTGCTGCTTC	Detection of mRNA

### Statistical analysis

The Z score was calculated for each well in a 48-well plate as described previously [82]. For a given well, the Z score was calculated by subtracting the mean value of the wells on that plate from the value of the given well and dividing by the standard deviation value for all of the plate wells. A Z score ≤ − 2 or ≥ 2 was considered significant. All data are presented as mean ± standard error (SEM) of triplicate samples per experimental condition from three independent experiments using GraphPad Prism 5 software (La Jolla, USA). To detect significant differences between experimental groups, an one-way analysis of variance (ANOVA) followed by Bonferroni’s multiple comparison test were conducted. Statistical significance was determined at *p* values of < 0.05 (*), < 0.01(**), or < 0.001 (***).

## Supplementary information

**Additional file 1: Figure S1.** Growth curves of *dam*, *invC*, *hilD*, *prgH*, and *spaN* gene deletion mutants, complementation, and overexpression strains. **Figure S2.** Two rounds of screening to identify the genes involved in regulating inflammasome activation in vitro (original images of immunoblotting). **Figure S3.** Deletion mutants of *dam*, *invC*, *prgH*, and *spaN* failed to induce inflammasome activation (original images of immunoblotting). **Figure S4.** Deletion of *prgH* did not influence the synthesis of inflammasome components (original images of immunoblotting). **Figure S5.** Overexpression of Dam inhibited inflammasome activation (original images of immunoblotting). **Figure S6.** The ability of the *dam* complementation strain and overexpression strain cultured without IPTG to activate the inflammasome was improved (original images of immunoblotting). **Figure S7.** The deletion of Dam blocked the Jnk pathway in infected J774A.1 cells (original images of immunoblotting). **Figure S8.** Dam independently promoted NLRP3 inflammasome activation (original images of immunoblotting).

## Data Availability

All data generated or analyzed during this study are included in this published article. Any additional information will be made available from the corresponding author on reasonable request. RNA sequences were deposited at NCBI Sequence Read Archive (SRA) with accession number SRP253144.

## Abbreviations

SE: *Salmonella* Enteritidis
SPI: *Salmonella* pathogenicity island
T3SS: type III secretion system
SCV: *Salmonella*-containing vacuole
PRRs: pattern recognition receptors
NLRs: Nod-like receptors
AIM2: Melanoma 2
ASC: Apoptosis-associated speck-like proteins containing caspase recruitment domains
IL-1β: Interleukin-1β
WT: Wild type
Dam: DNA adenine methylase
LDH: Lactate dehydrogenase
DEGs: Differentially expressed genes
LB: Luria-Bertani
DAP: Diaminopimelic acid
PBS: Phosphate-buffered saline
DMEM: Dulbecco’s modified Eagle’s medium
FBS: Fetal bovine serum
M-CSF: Macrophage colony-stimulating factor
DPBS: Dulbecco’s phosphate-buffered saline
dpc: Days post-challenge
ELISA: Enzyme-linked immunosorbent assay
FRET: Fluorescence resonance energy transfer
Km^r^: Kanamycin
Cm^r^: Chloramphenicol
Tet^r^: Tetracycline
MAPK: Mitogen-activated protein kinase
KEGG: Kyoto Encyclopedia of Genes and Genomes
TNF: Tumor necrosis factor
qRT-PCR: Quantitative real-time PCR
GAPDH: Glyceraldehyde-3-phosphate dehydrogenase
Jnk: C-Jun N-terminal kinase
ERK1/2: Extracellular regulated protein kinases 1/2
GFP: Green fluorescent protein
ATP: Adenosine triphosphate
FoxO: Forkhead box, sub-group O
Xiap: X-linked inhibitor of apoptosis protein
ROS: Reactive oxygen species
