# Supplementary material for: Essential role of Salmonella Enteritidis DNA adenine methylase in modulating inflammasome activation
Source: BMC Microbiol. 2020 Jul 28;20:226. doi: 10.1186/s12866-020-01919-z (PMC7389876; doi:10.1186/s12866-020-01919-z)
Supplement: Supplementary file 1 — Additional file 1: Figure S1. Growth curves of dam, invC, hilD, prgH, and spaN gene deletion mutants, complementation, and overexpression strains. Figure S2. Two rounds of screening to identify the genes involved in regulating inflammasome activation in vitro (original images of immunoblotting). Figure S3. Deletion mutants of dam, invC, prgH, and spaN failed to induce inflammasome activation (original images of immunoblotting). Figure S4. Deletion of prgH did not influence the synthesis of inflammasome components (original images of immunoblotting). Figure S5. Overexpression of Dam inhibited inflammasome activation (original images of immunoblotting). Figure S6. The ability of the dam complementation strain and overexpression strain cultured without IPTG to activate the inflammasome was improved (original images of immunoblotting). Figure S7. The deletion of Dam blocked the Jnk pathway in infected J774A.1 cells (original images of immunoblotting). Figure S8. Dam independently promoted NLRP3 inflammasome activation (original images of immunoblotting). [file 12866_2020_1919_MOESM1_ESM.pdf]

Supplemental Information

Essential role of *Salmonella* Enteritidis DNA adenine methylase in modulating inflammasome activation

Yaxin Guo, Dan Gu, Tingting Huang, Liyan Cao, Xinyu Zhu, Yi Zhou, Kangru Wang, Xilong Kang, Chuang Meng, Xinan Jiao, Zhiming Pan

Figure S1

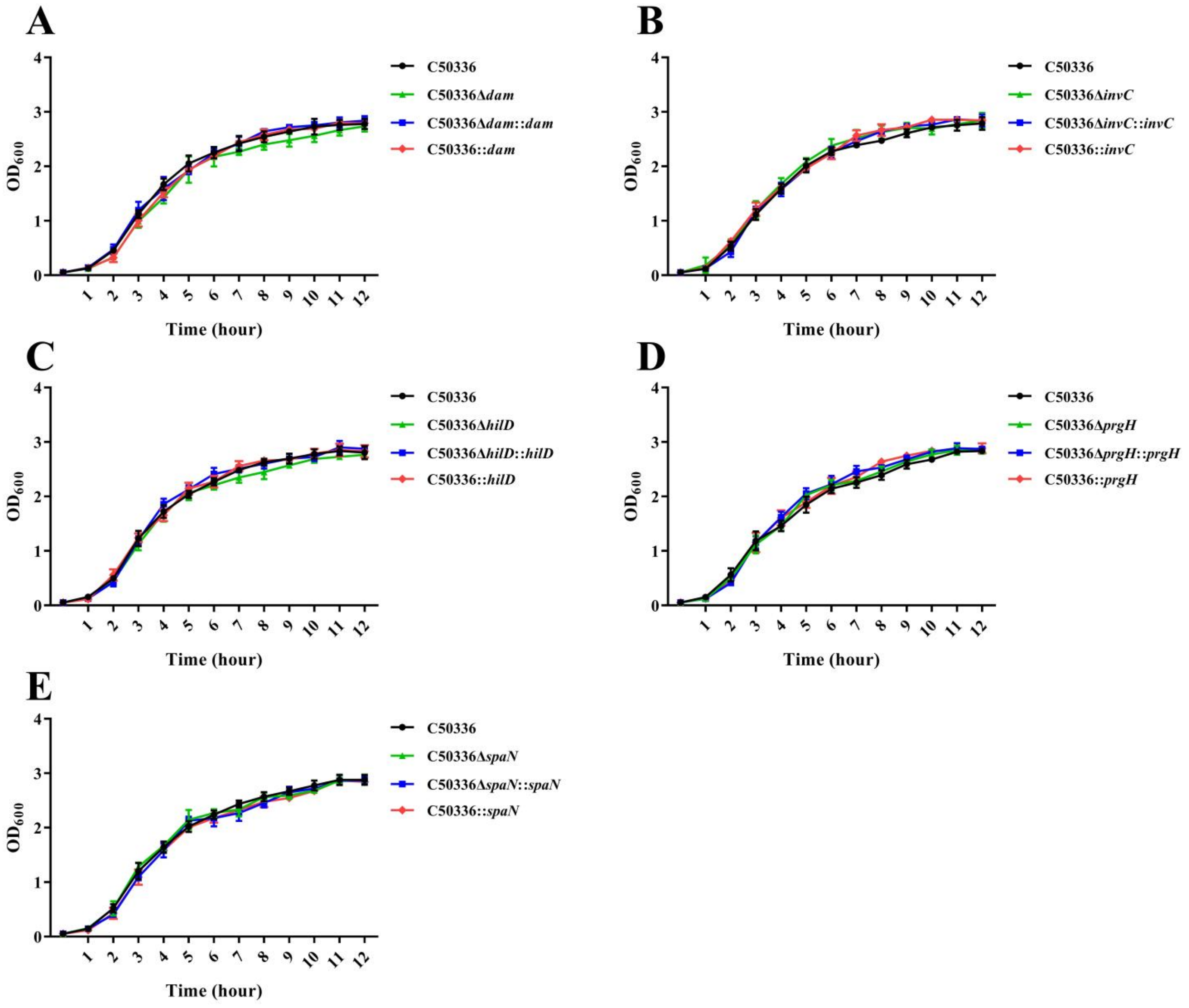

A
C50336
C50336Δdam
C50336Δdam::dam
C50336::dam
B
C50336
C50336ΔinvC
C50336ΔinvC::invC
C50336::invC
C
C50336
C50336ΔhilD
C50336ΔhilD::hilD
C50336::hilD
D
C50336
C50336ΔprgH
C50336ΔprgH::prgH
C50336::prgH
E
C50336
C50336ΔspaN
C50336ΔspaN::spaN
C50336::spaN
OD_{600}
Time (hour)

Figure S2

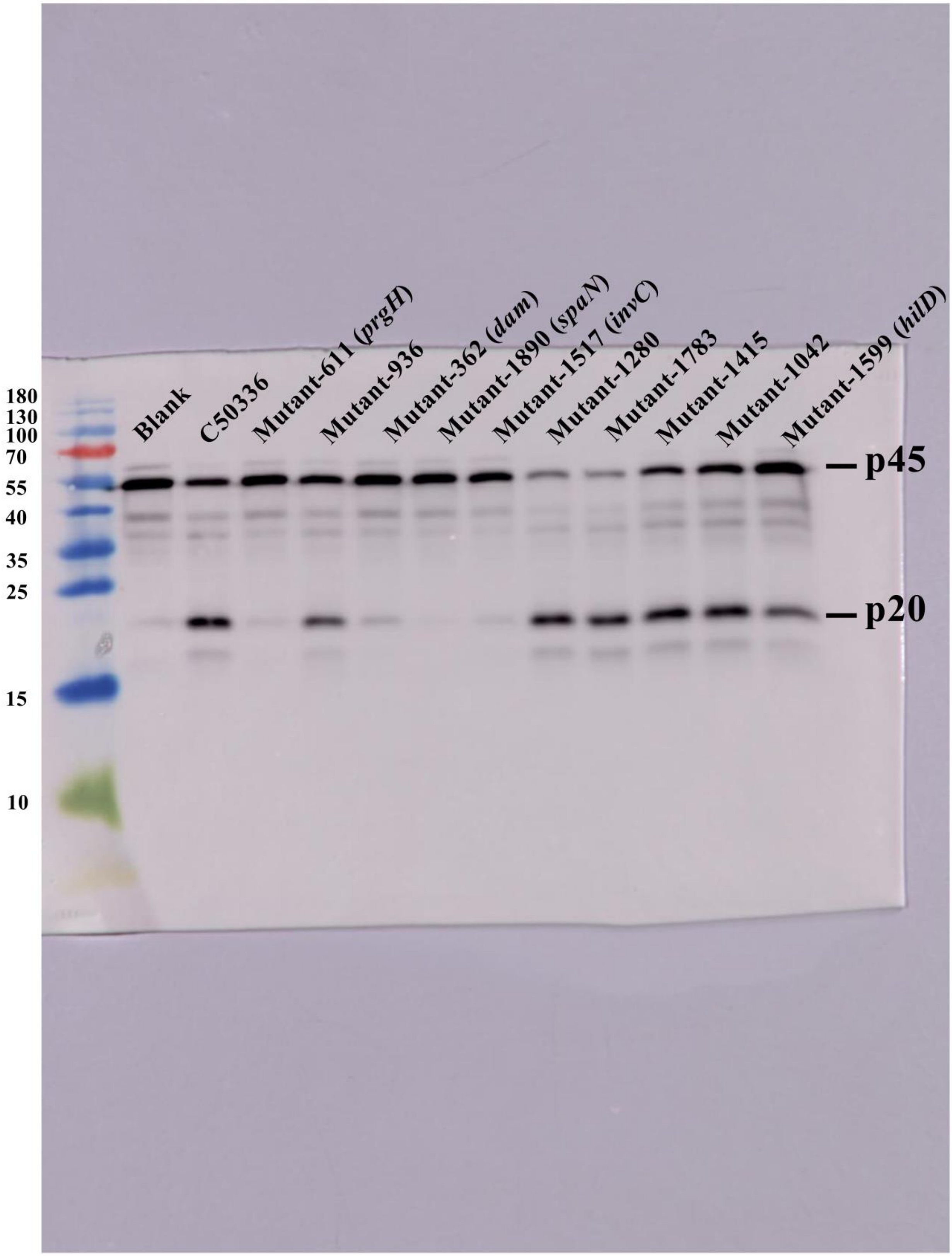

180
130
100
70
55
40
35
25
15
10
Blank
C50336
Mutant-611 (prgH)
Mutant-936
Mutant-362 (dam)
Mutant-1890 (spaN)
Mutant-1517 (invC)
Mutant-1280
Mutant-1783
Mutant-1415
Mutant-1042
Mutant-1599 (hilD)
p45
p20

Figure S2

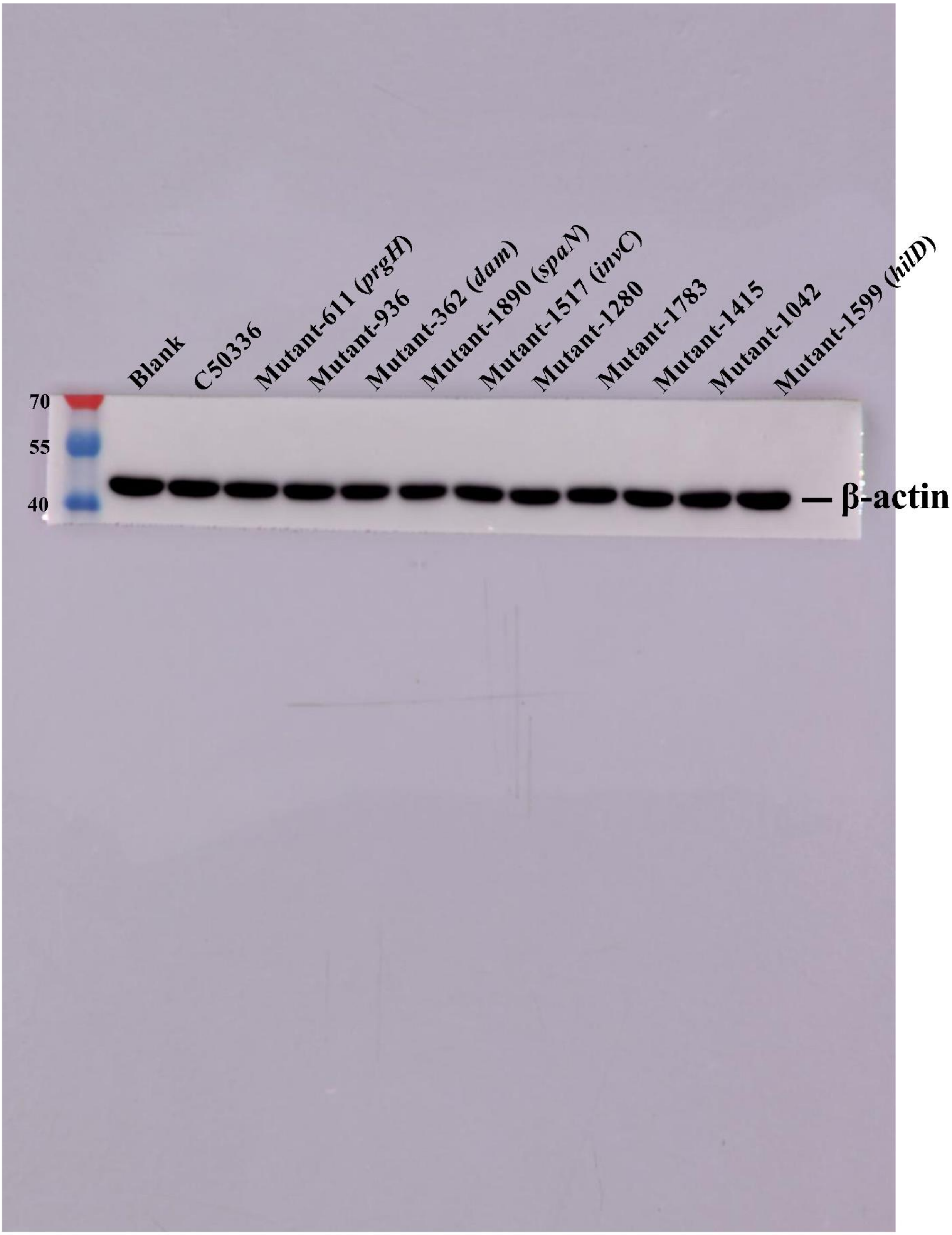
Blank
C50336
Mutant-611 (prgH)
Mutant-936
Mutant-362 (dam)
Mutant-1890 (spaN)
Mutant-1517 (invC)
Mutant-1280
Mutant-1783
Mutant-1415
Mutant-1042
Mutant-1599 (hilD)
70
55
40
β-actin

Figure S3

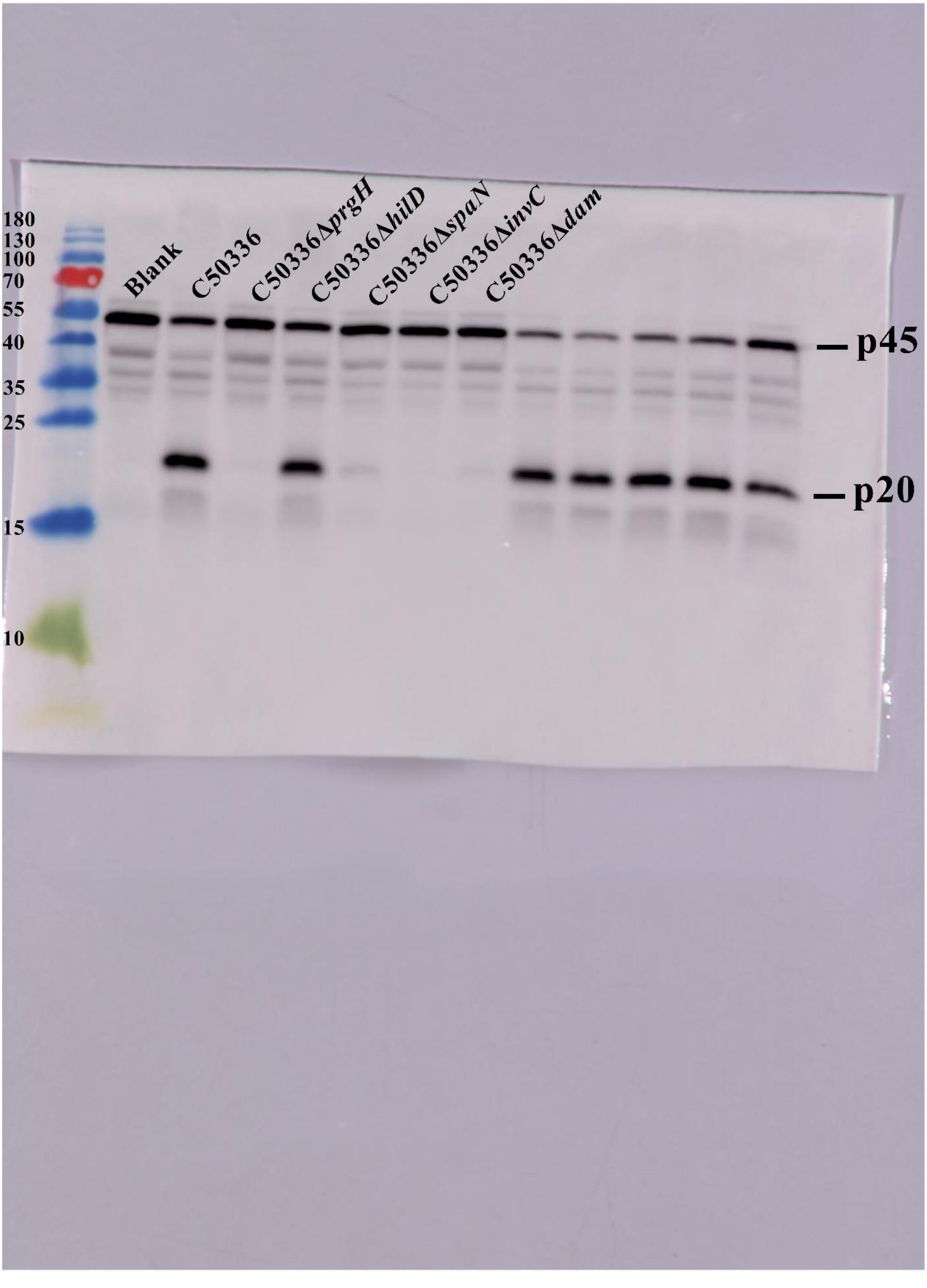
180
130
100
70
55
40
35
25
15
10
Blank
C50336
C50336ΔprgH
C50336ΔhilD
C50336ΔspaN
C50336ΔinvC
C50336Δdam
p45
p20

Figure S3

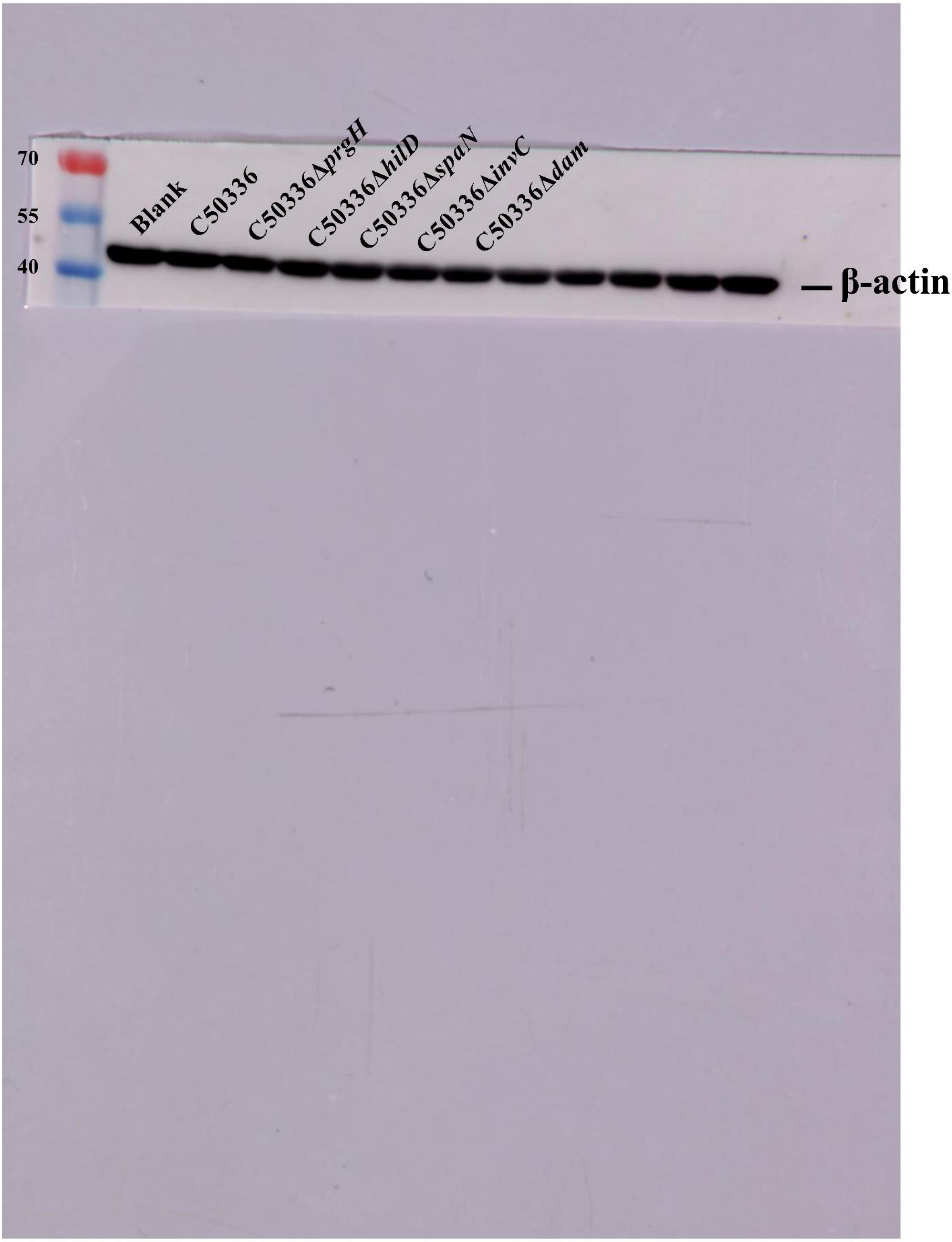
70
55
40
Blank
C50336
C50336ΔprgH
C50336ΔhilD
C50336ΔspaN
C50336ΔinvC
C50336Δdam
β-actin

Figure S4

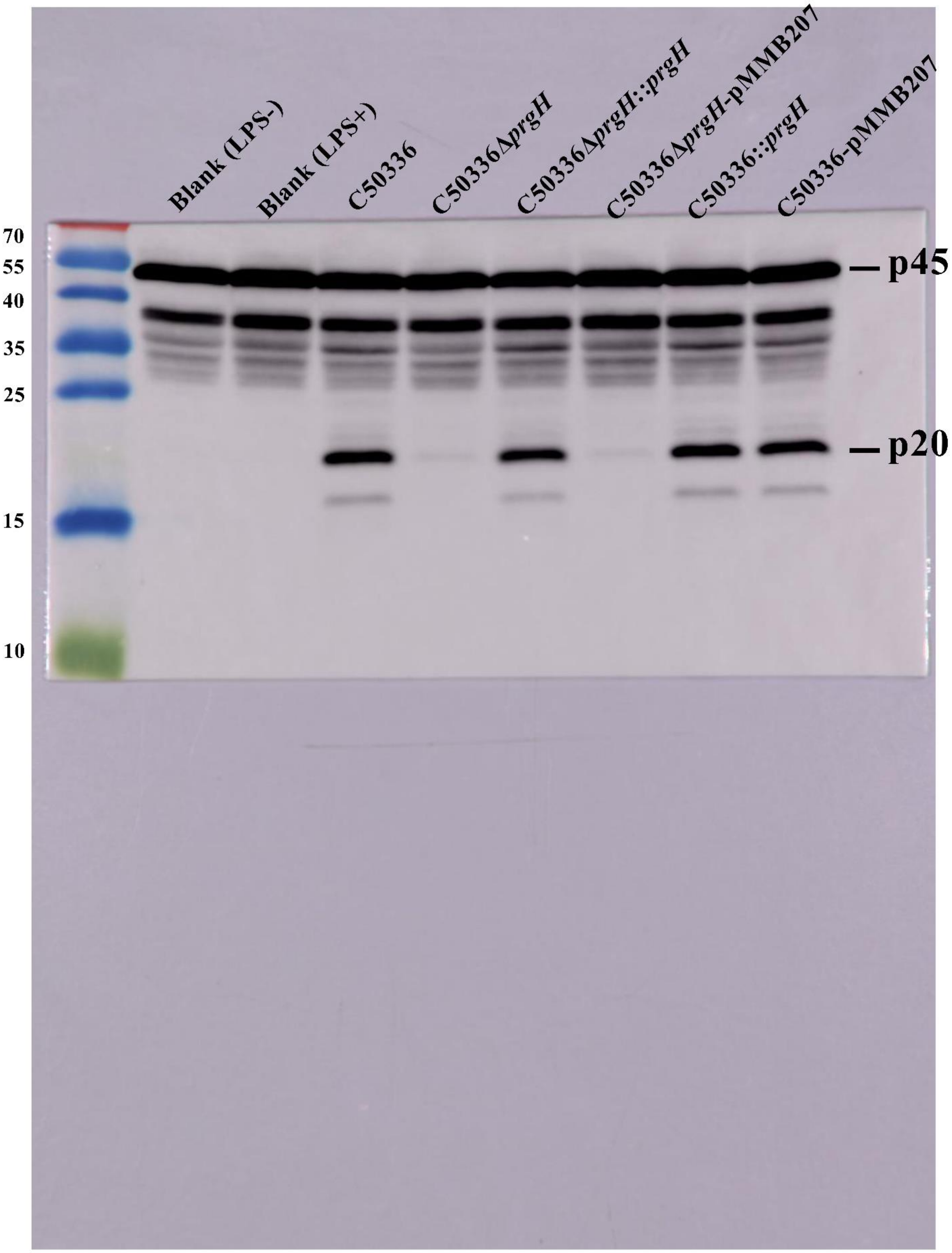
Blank (LPS-)
Blank (LPS+)
C50336
C50336Δ*prgH*
C50336Δ*prgH*::*prgH*
C50336Δ*prgH*-pMMB207
C50336::*prgH*
C50336-pMMB207
70
55
40
35
25
15
10
p45
p20

Figure S4

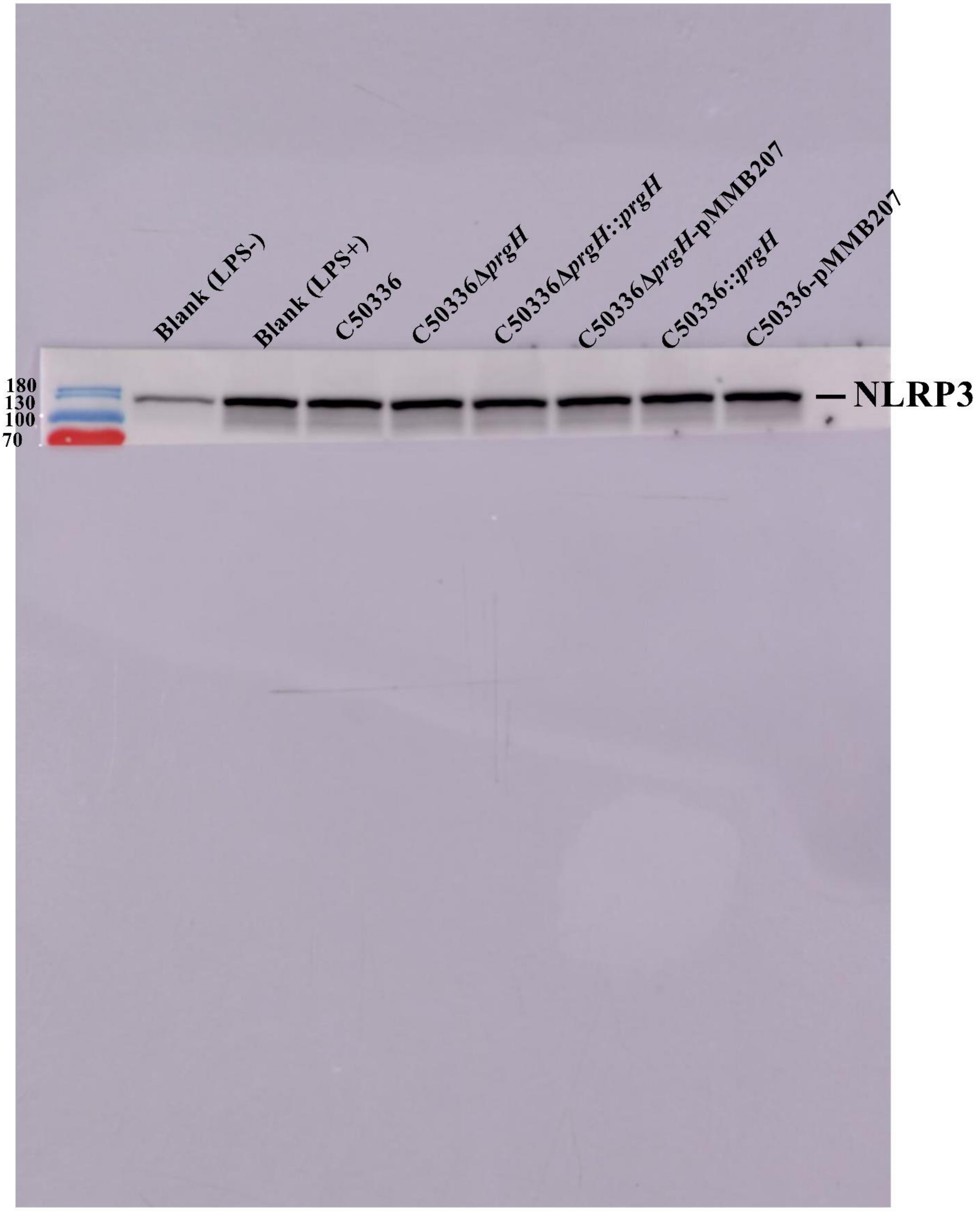

Blank (LPS-)
Blank (LPS+)
C50336
C50336Δ*prgH*
C50336Δ*prgH*::*prgH*
C50336Δ*prgH*-pMMB207
C50336::*prgH*
C50336-pMMB207
180
130
100
70
NLRP3

Figure S4

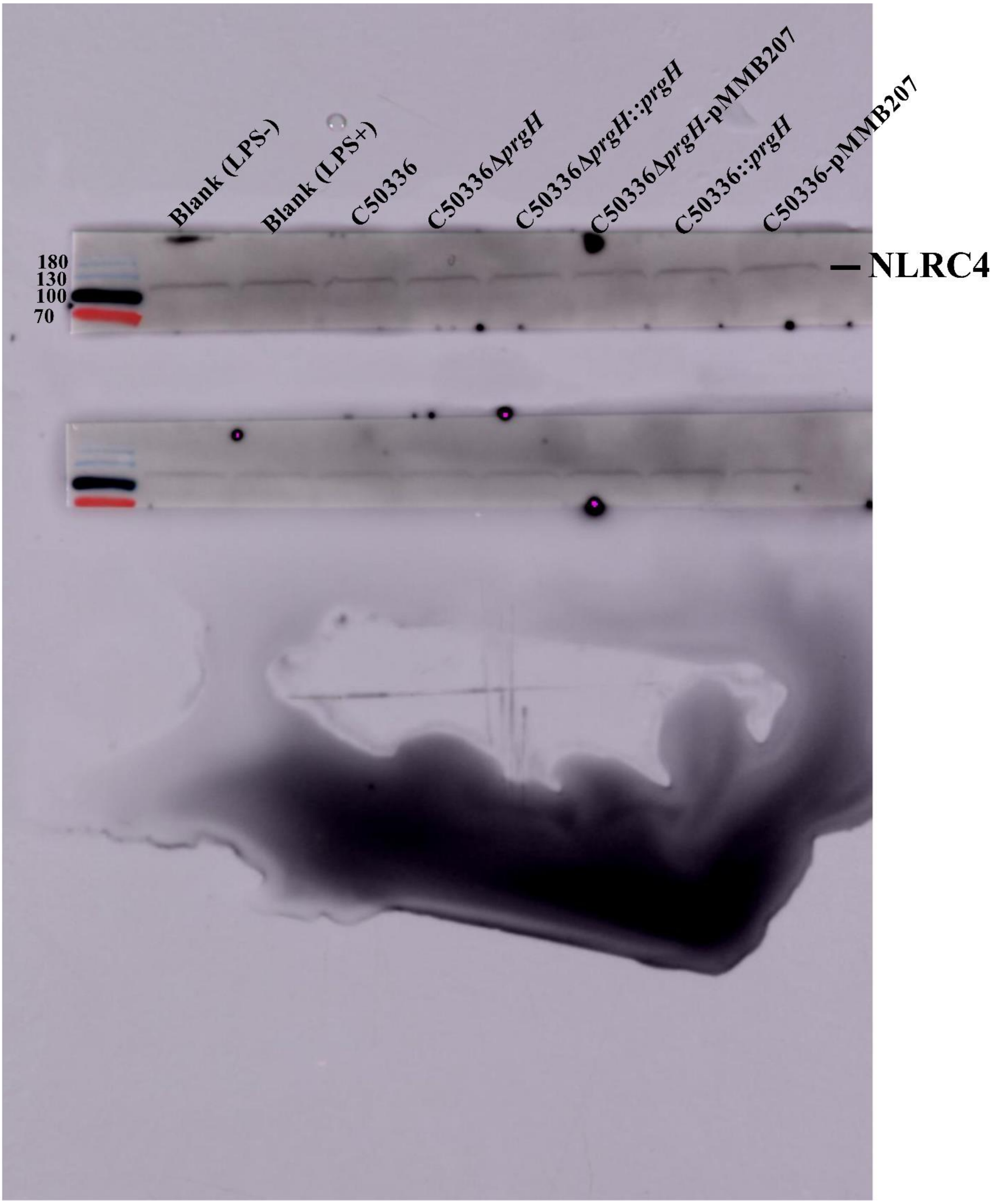

Blank (LPS-)
Blank (LPS+)
C50336
C50336Δ*prgH*
C50336Δ*prgH*::*prgH*
C50336Δ*prgH*-pMMB207
C50336::*prgH*
C50336-pMMB207
180
130
100
70
NLRC4

Figure S4

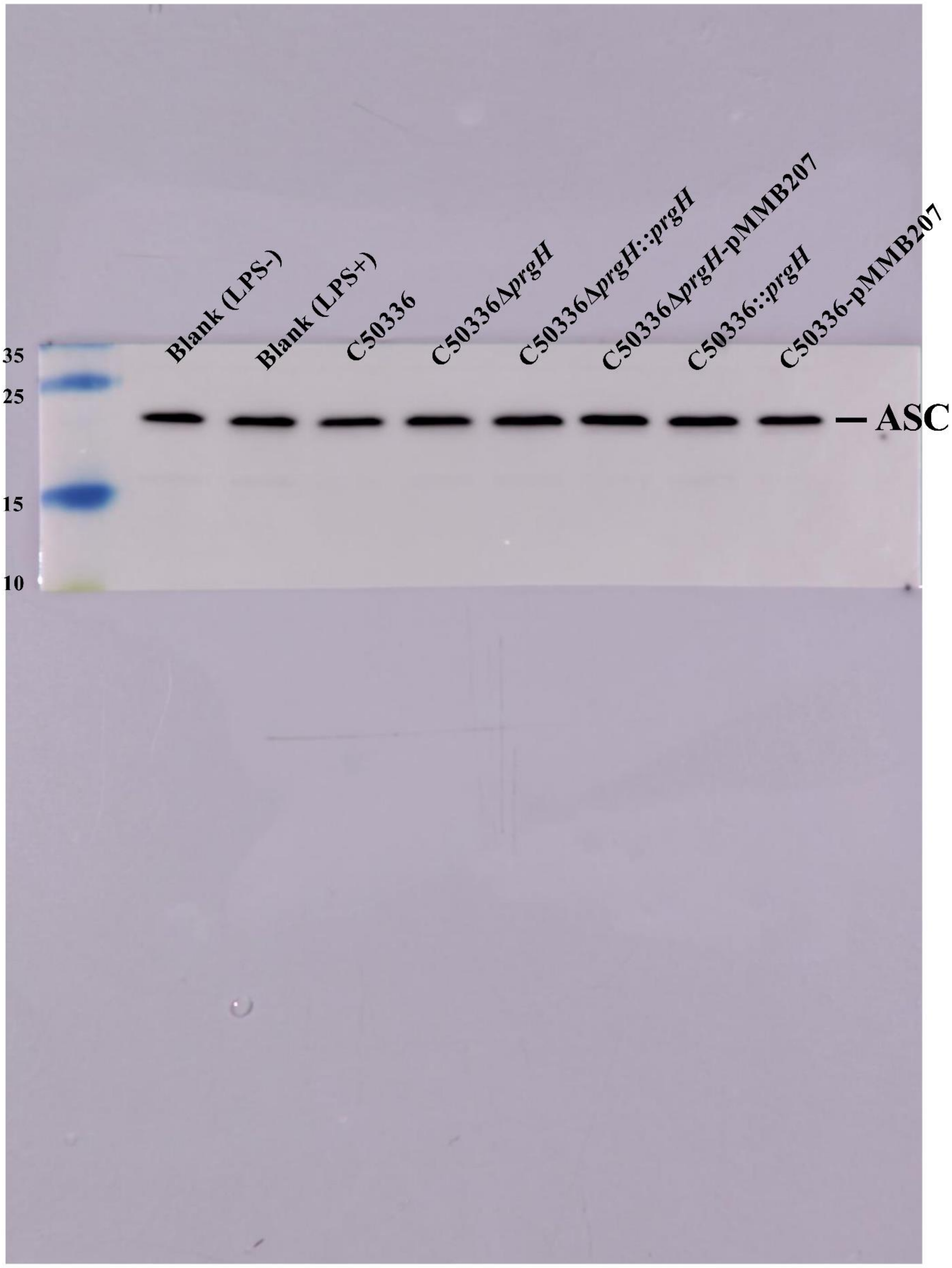

Blank (LPS-)
Blank (LPS+)
C50336
C50336Δ*prgH*
C50336Δ*prgH*::*prgH*
C50336Δ*prgH*-pMMB207
C50336::*prgH*
C50336-pMMB207
35
25
15
10
ASC

Figure S4

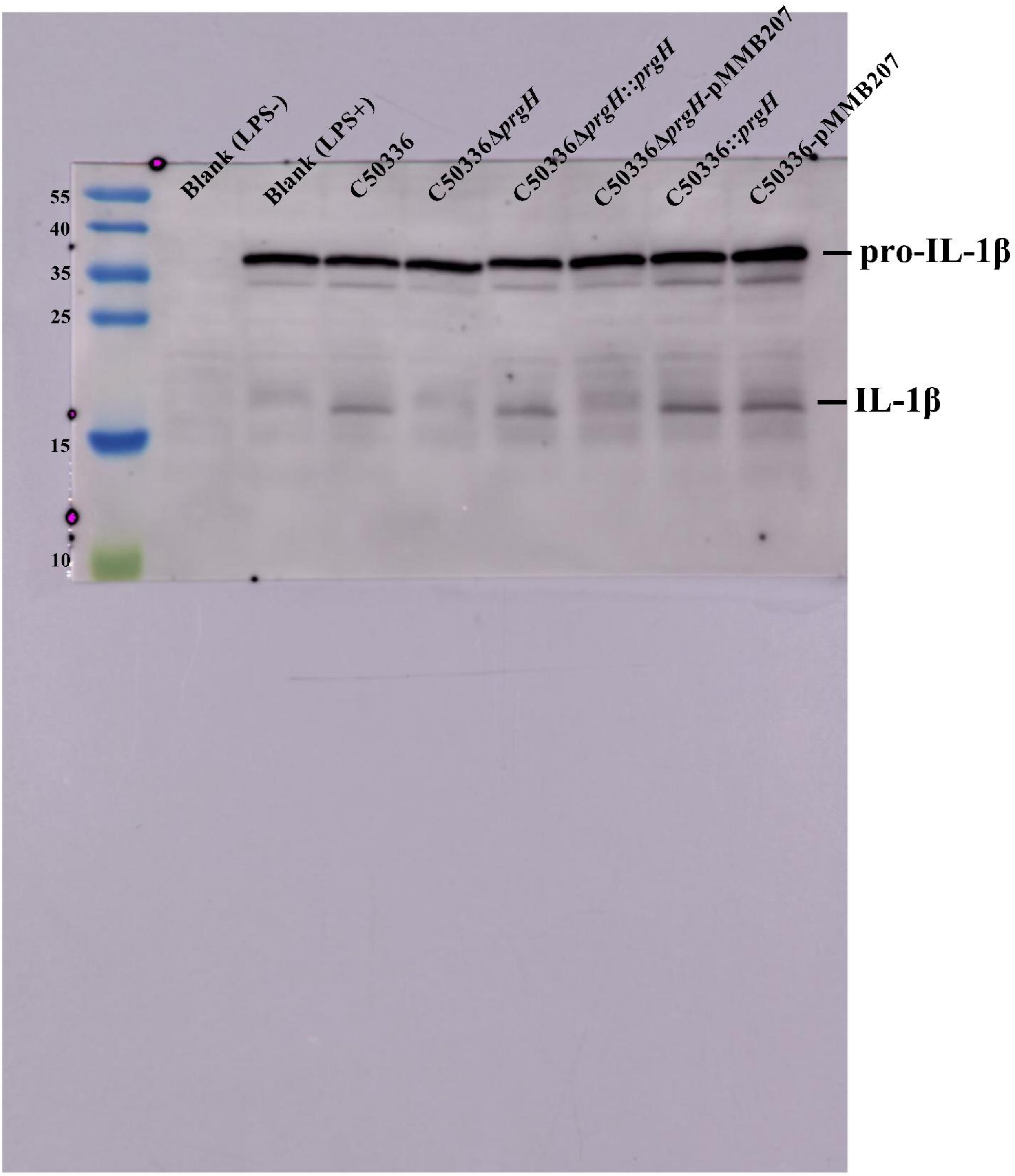
Blank (LPS-)
Blank (LPS+)
C50336
C50336Δ*prgH*
C50336Δ*prgH*::*prgH*
C50336Δ*prgH*-pMMB207
C50336::*prgH*
C50336-pMMB207
55
40
35
25
15
10
pro-IL-1β
IL-1β

Figure S4

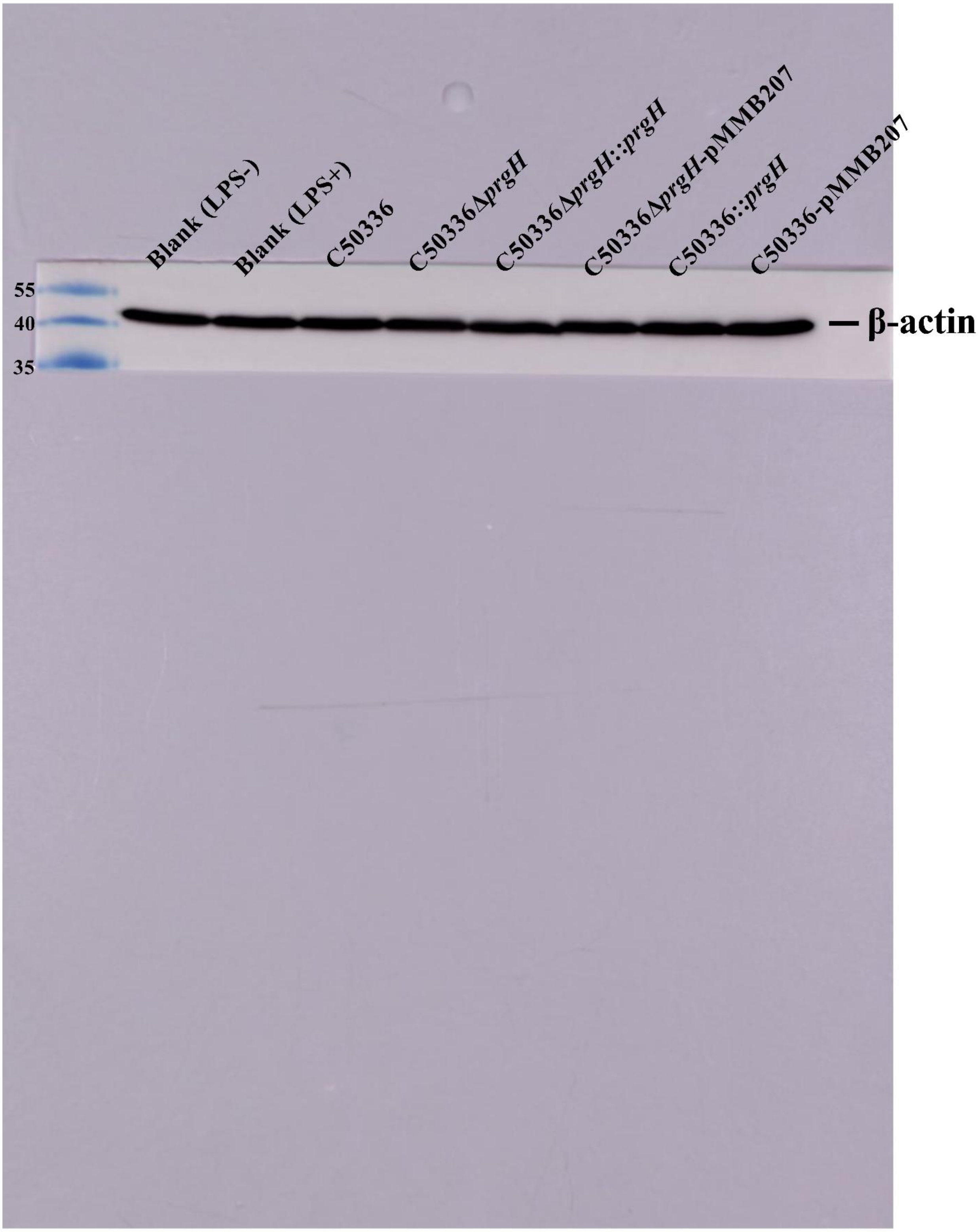
Blank (LPS-)
Blank (LPS+)
C50336
C50336ΔprgH
C50336ΔprgH::prgH
C50336ΔprgH-pMMB207
C50336::prgH
C50336-pMMB207
55
40
35
β-actin

Figure S5

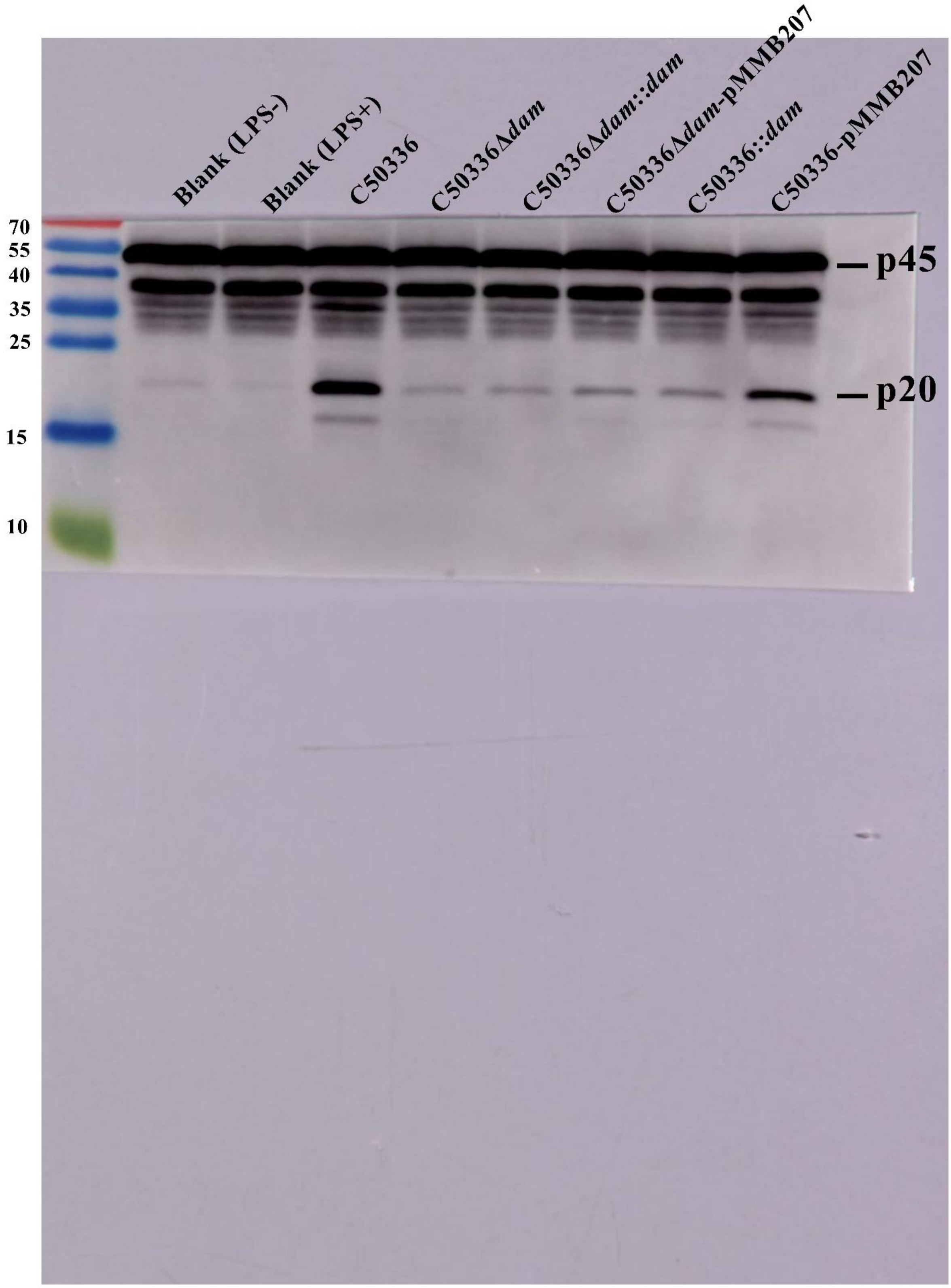

Blank (LPS-)
Blank (LPS+)
C50336
C50336Δ*dam*
C50336Δ*dam*::*dam*
C50336Δ*dam*-pMMB207
C50336::*dam*
C50336-pMMB207
70
55
40
35
25
15
10
p45
p20

Figure S5

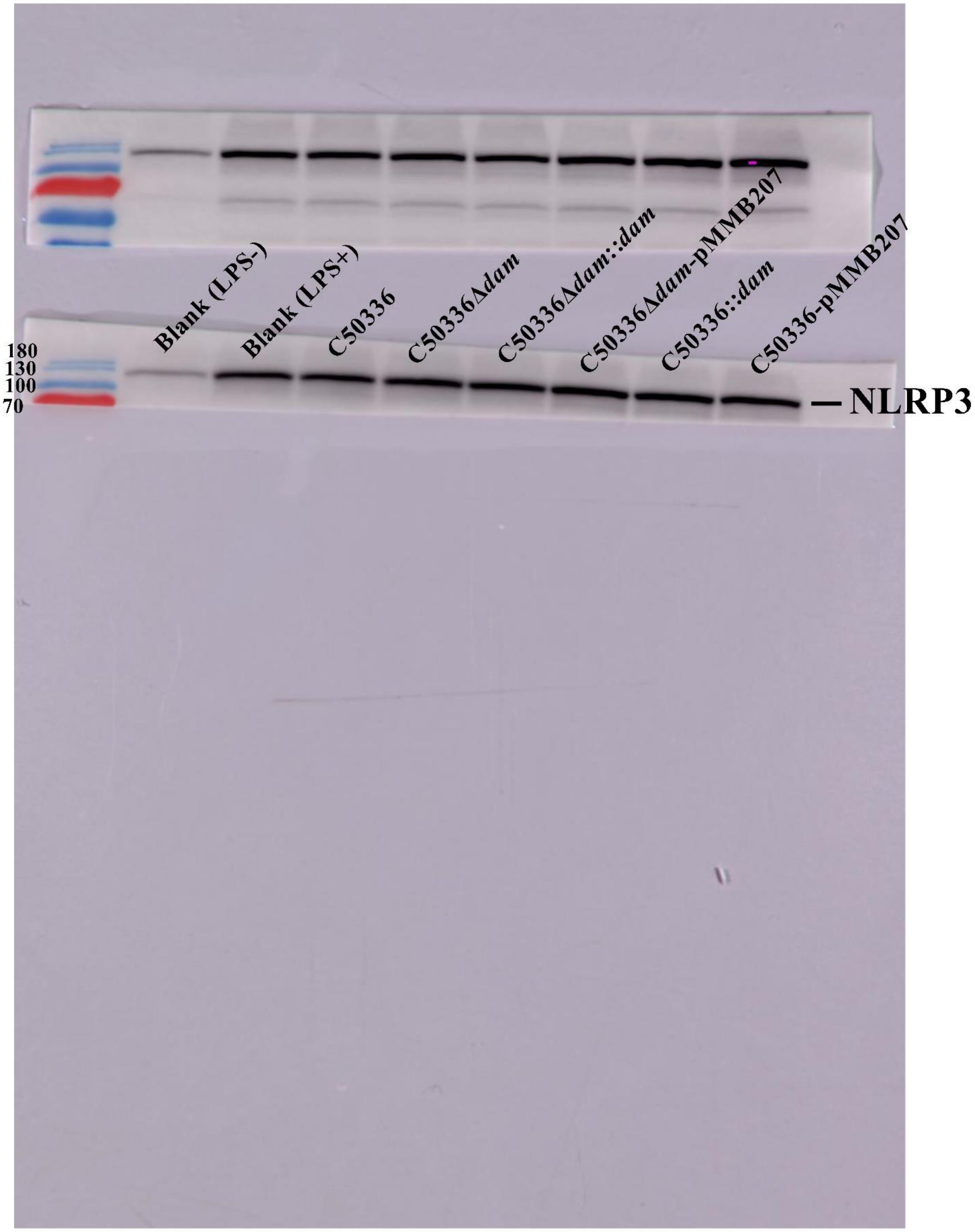

180
130
100
70
Blank (LPS-)
Blank (LPS+)
C50336
C50336Δdam
C50336Δdam::dam
C50336Δdam-pMMB207
C50336::dam
C50336-pMMB207
NLRP3

Figure S5

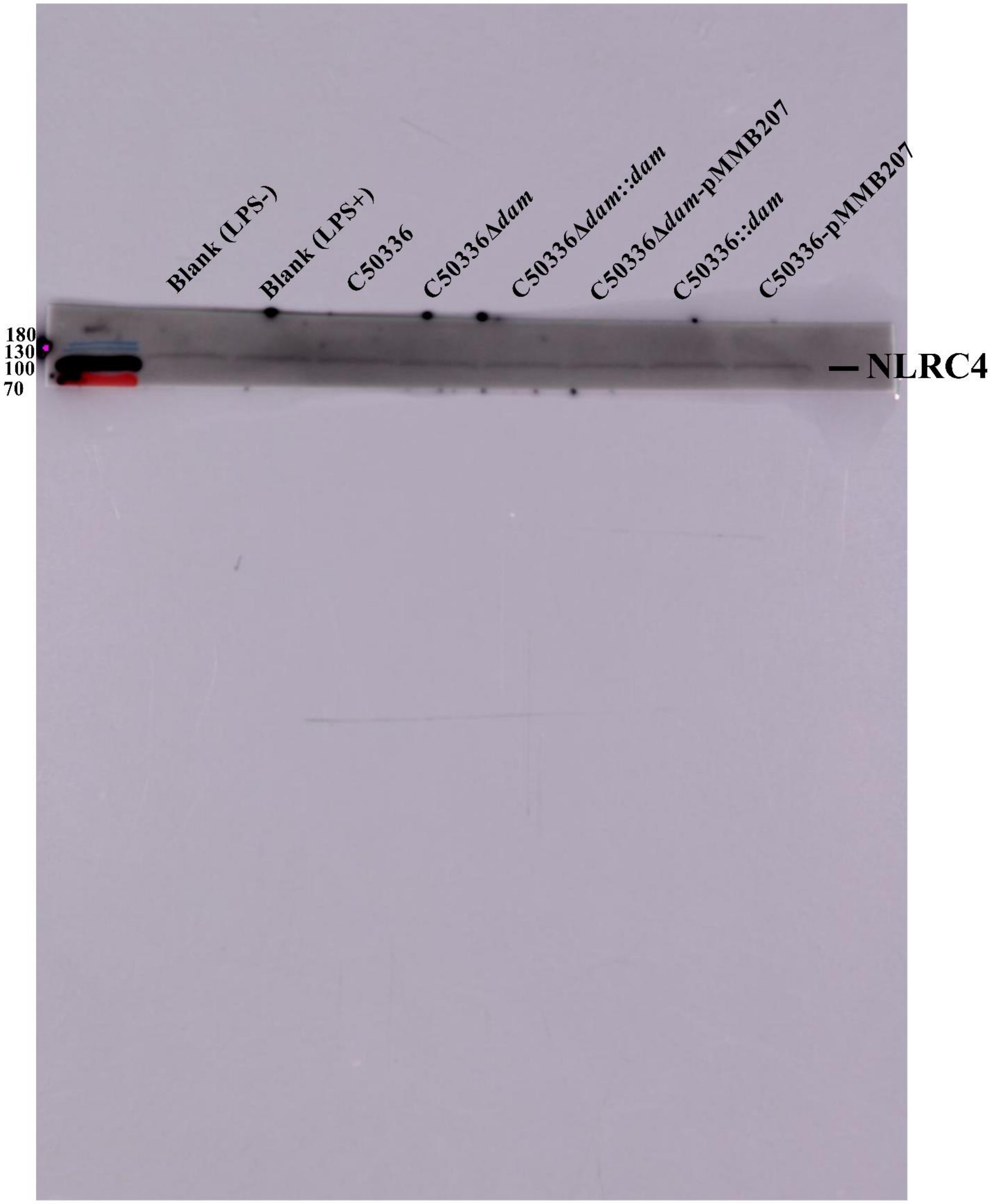

Blank (LPS-)
Blank (LPS+)
C50336
C50336Δ*dam*
C50336Δ*dam*::*dam*
C50336Δ*dam*-pMMB207
C50336::*dam*
C50336-pMMB207
180
130
100
70
NLRC4

Figure S5

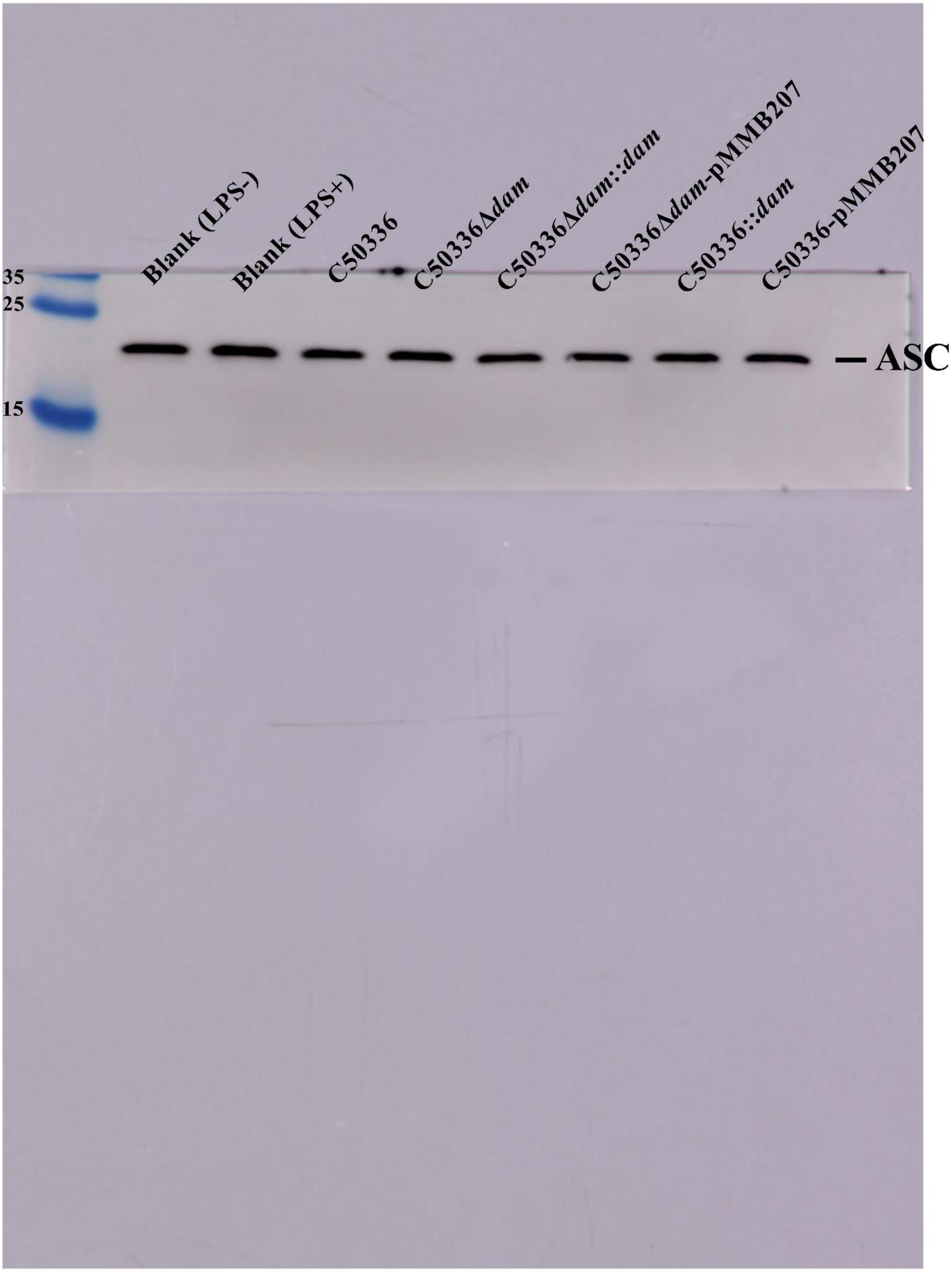

Blank (LPS-)
Blank (LPS+)
C50336
C50336Δ*dam*
C50336Δ*dam*::*dam*
C50336Δ*dam*-pMMB207
C50336::*dam*
C50336-pMMB207
35
25
15
ASC

Figure S5

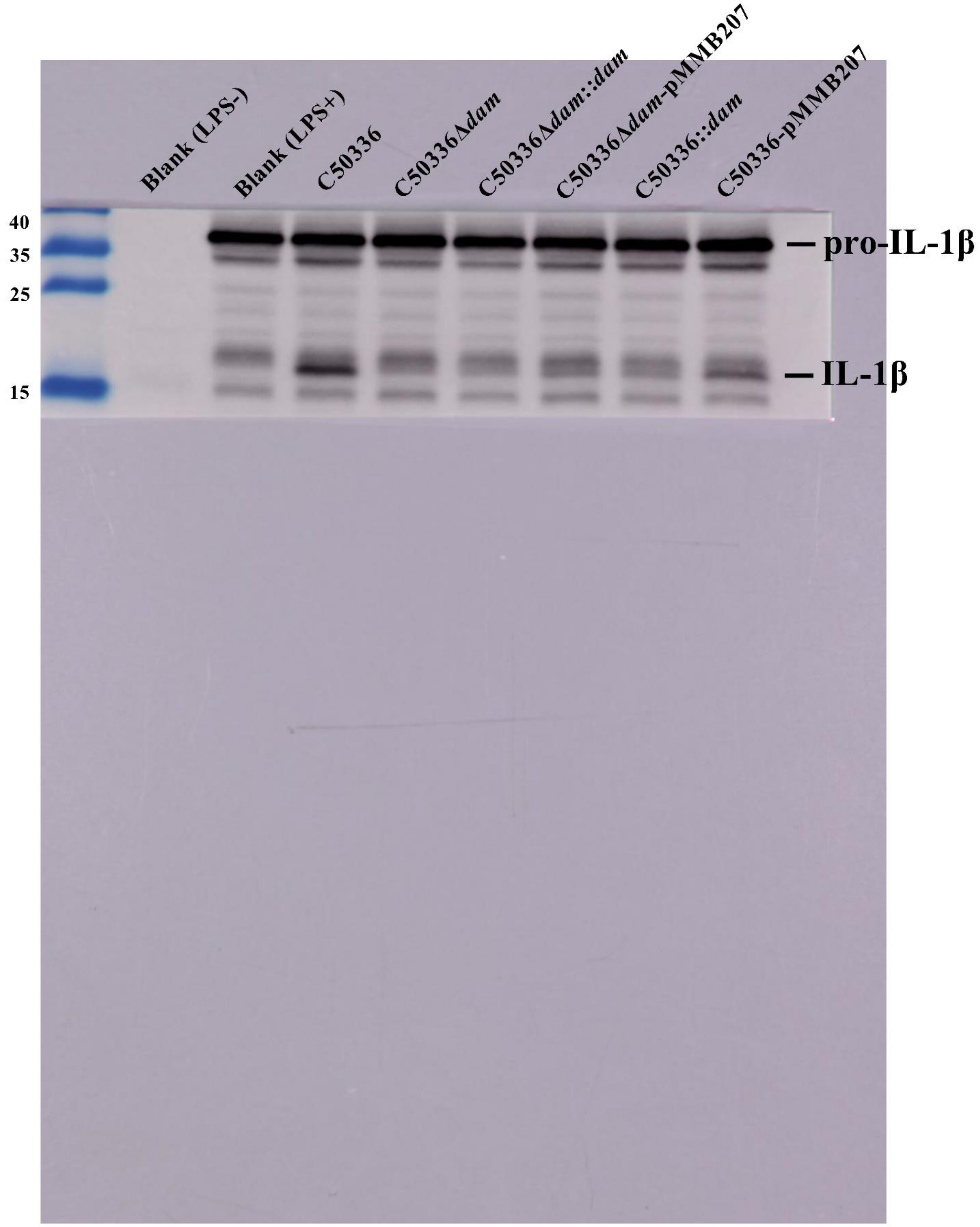

Blank (LPS-)
Blank (LPS+)
C50336
C50336Δdam
C50336Δdam::dam
C50336Δdam-pMMB207
C50336::dam
C50336-pMMB207
40
35
25
15
pro-IL-1β
IL-1β

Figure S5

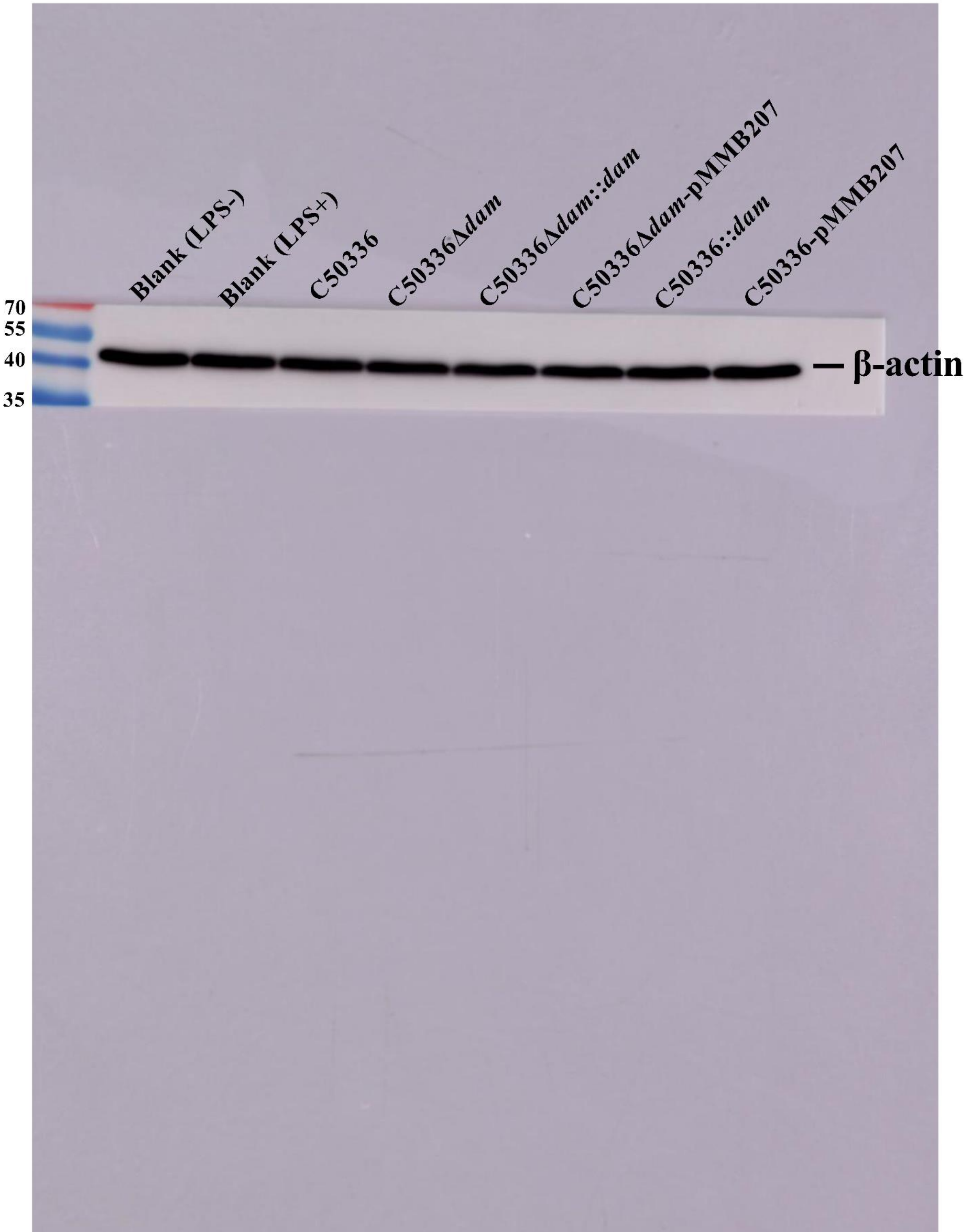
Blank (LPS-)
Blank (LPS+)
C50336
C50336Δ*dam*
C50336Δ*dam*::*dam*
C50336Δ*dam*-pMMB207
C50336::*dam*
C50336-pMMB207
70
55
40
35
β-actin

Figure S6

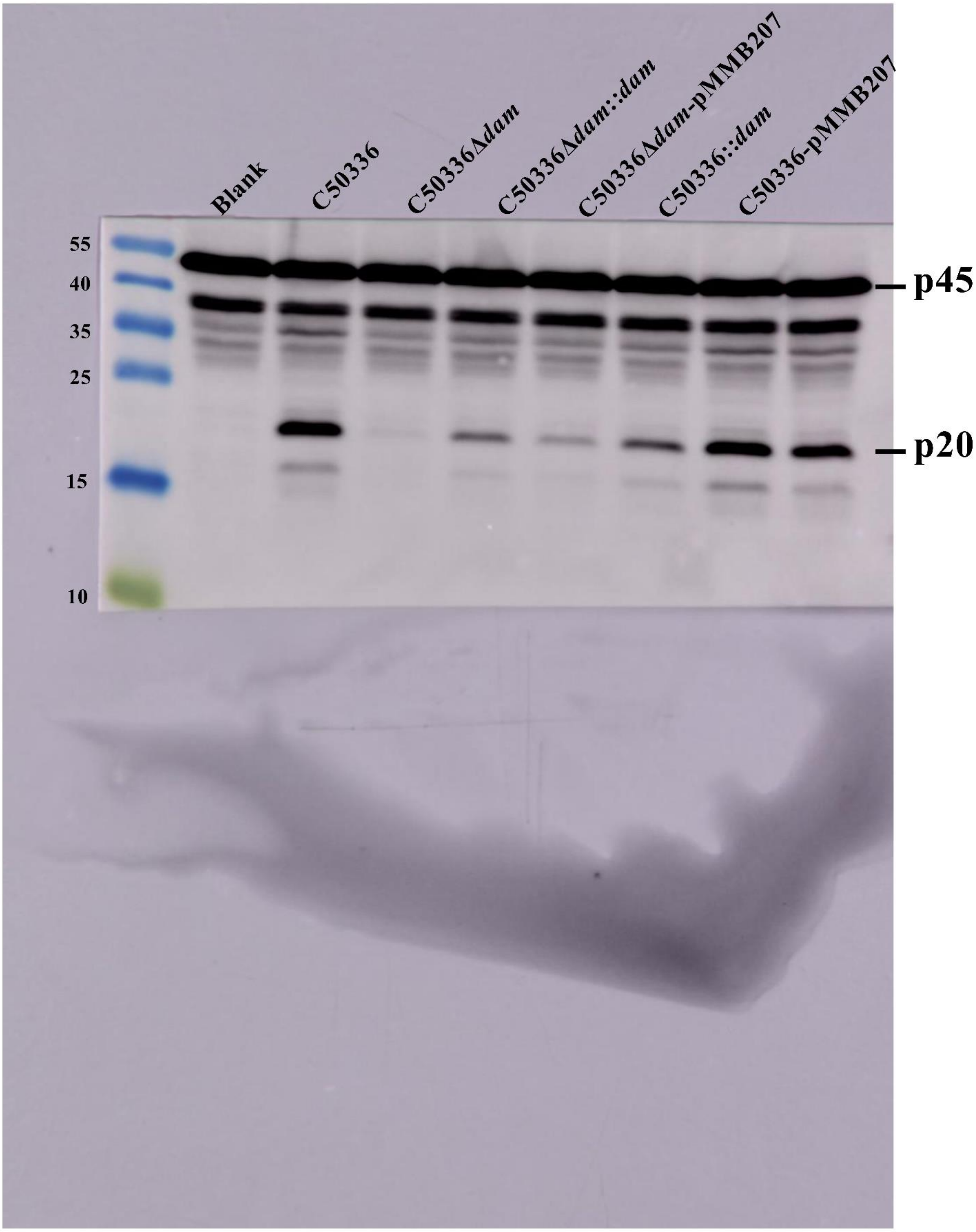

Blank
C50336
C50336Δ*dam*
C50336Δ*dam*::*dam*
C50336Δ*dam*-pMMB207
C50336::*dam*
C50336-pMMB207
55
40
35
25
15
10
p45
p20

Figure S6

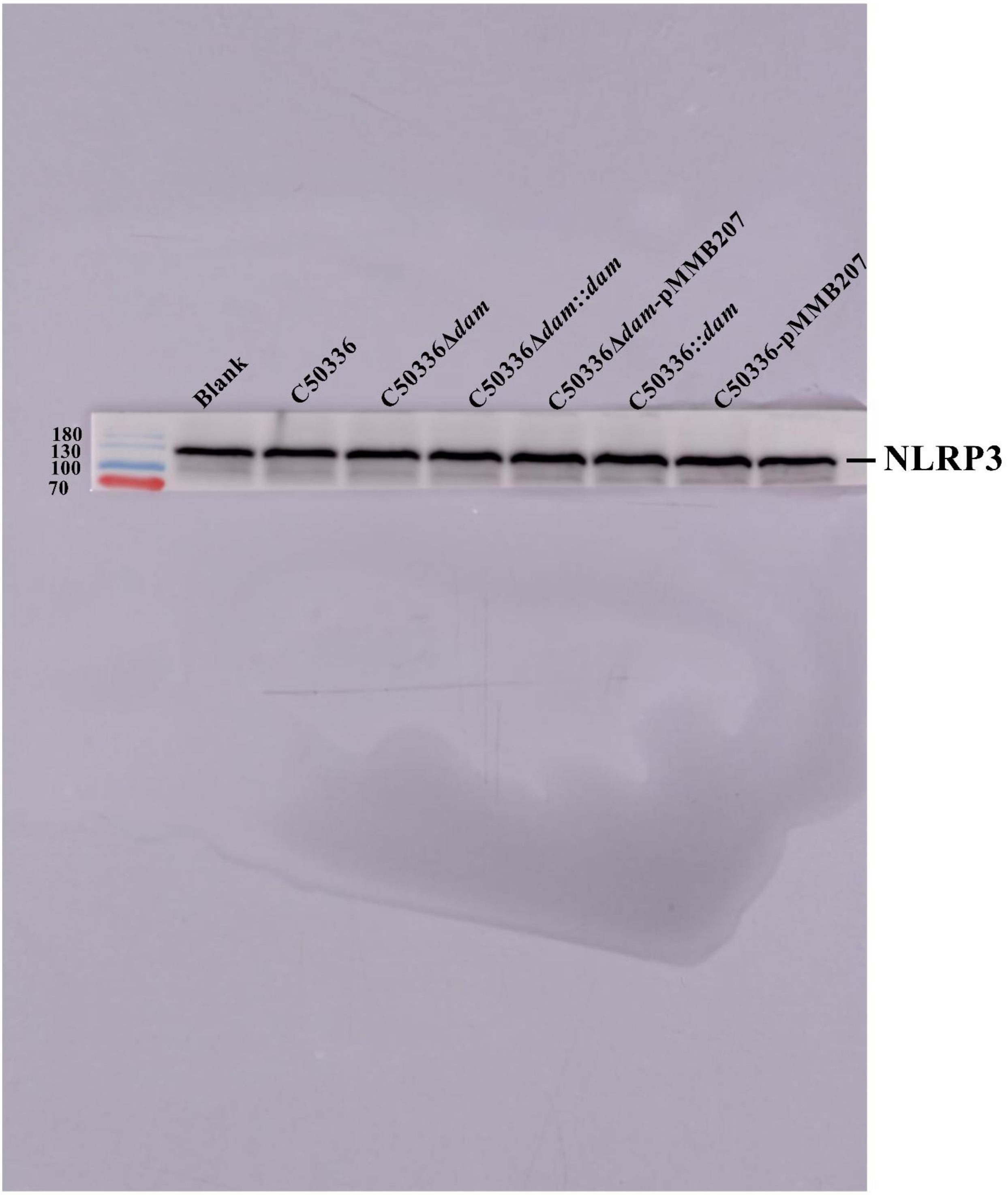

Blank
C50336
C50336Δdam
C50336Δdam::dam
C50336Δdam-pMMB207
C50336::dam
C50336-pMMB207
180
130
100
70
NLRP3

Figure S6

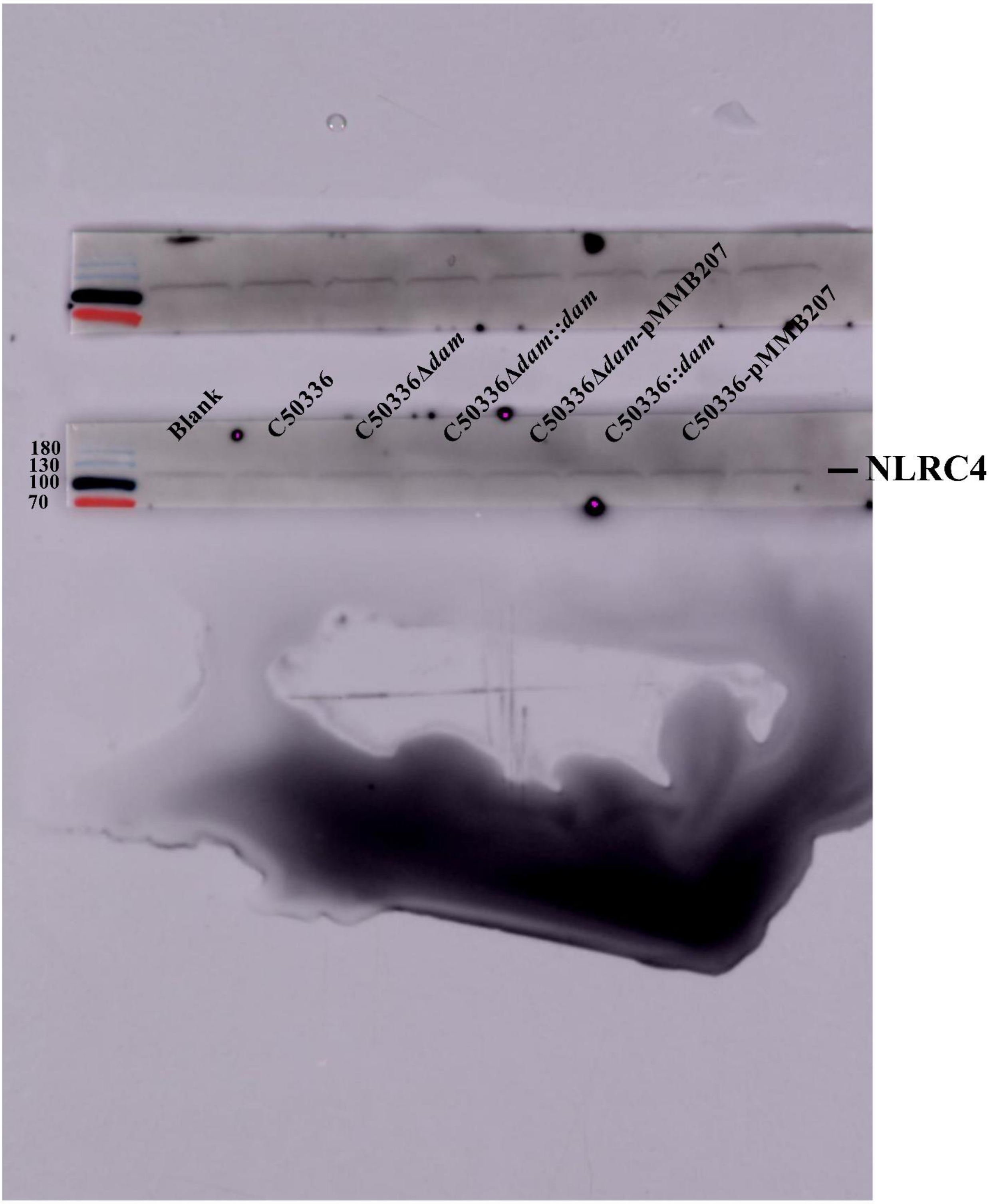
Blank
C50336
C50336Δ*dam*
C50336Δ*dam*::*dam*
C50336Δ*dam*-pMMB207
C50336::*dam*
C50336-pMMB207
180
130
100
70
NLRC4

Figure S6

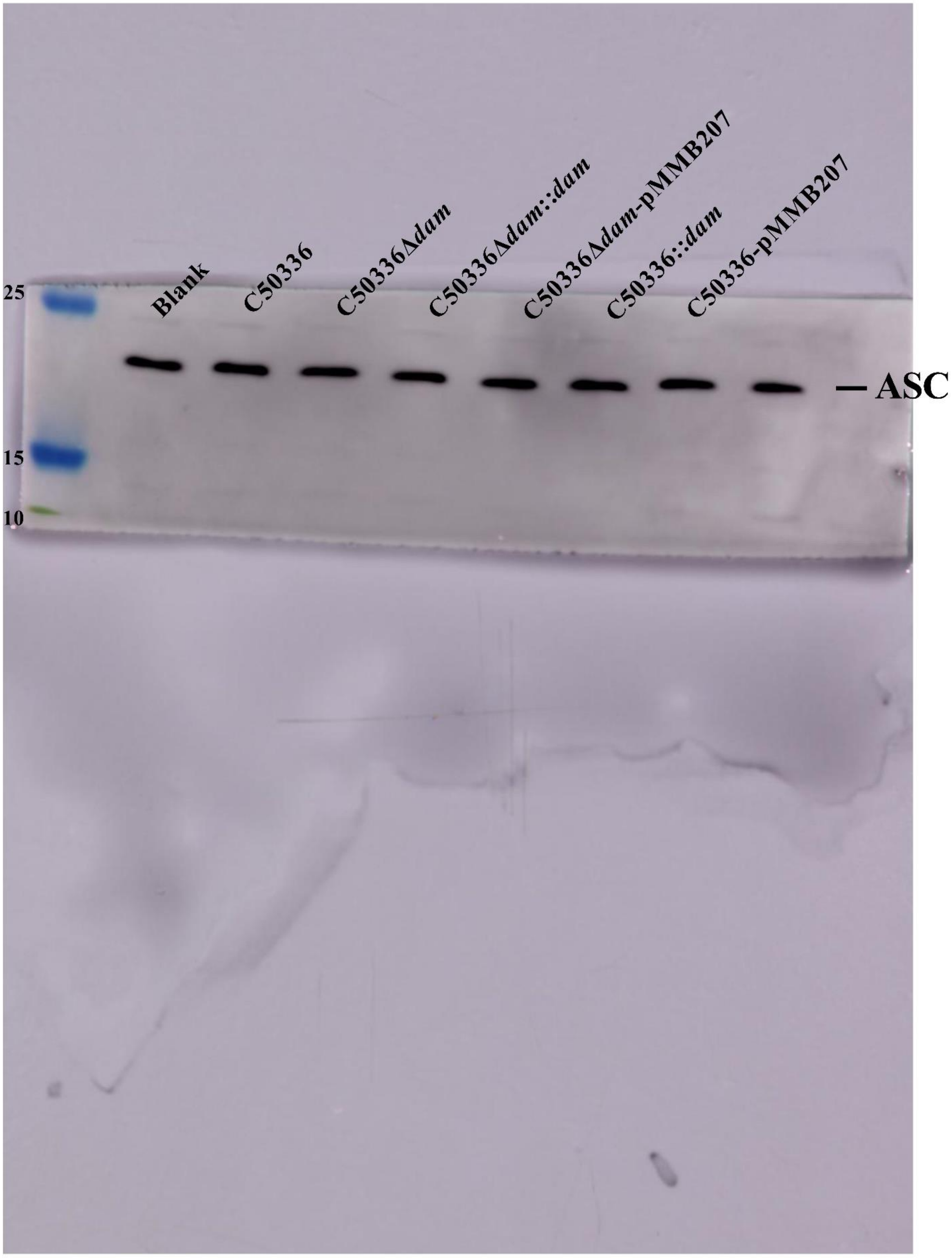
25
15
10
Blank
C50336
C50336Δ*dam*
C50336Δ*dam*::*dam*
C50336Δ*dam*-pMMB207
C50336::*dam*
C50336-pMMB207
ASC

Figure S6

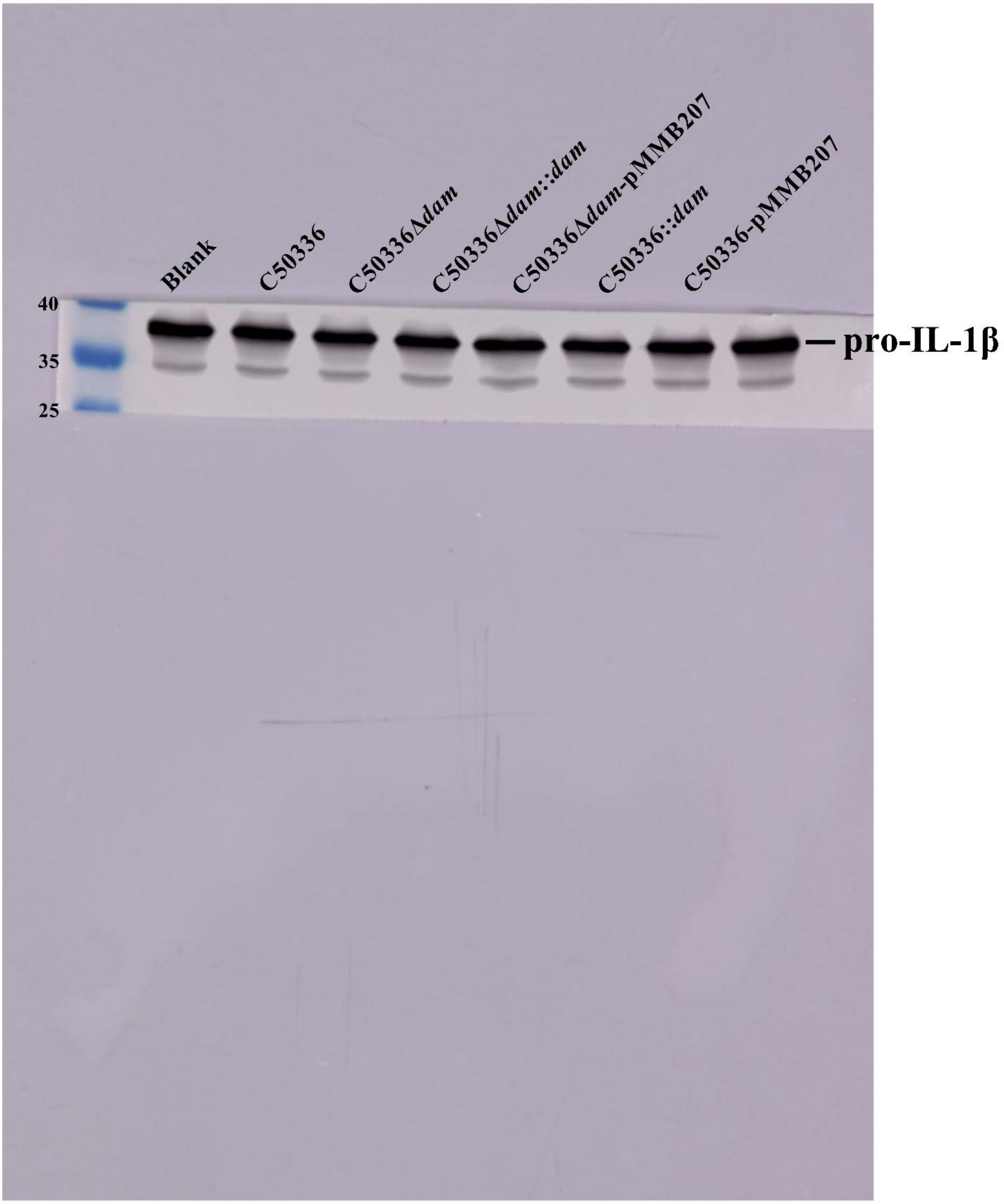
Blank
C50336
C50336Δ*dam*
C50336Δ*dam*::*dam*
C50336Δ*dam*-pMMB207
C50336::*dam*
C50336-pMMB207
40
35
25
pro-IL-1β

Figure S6

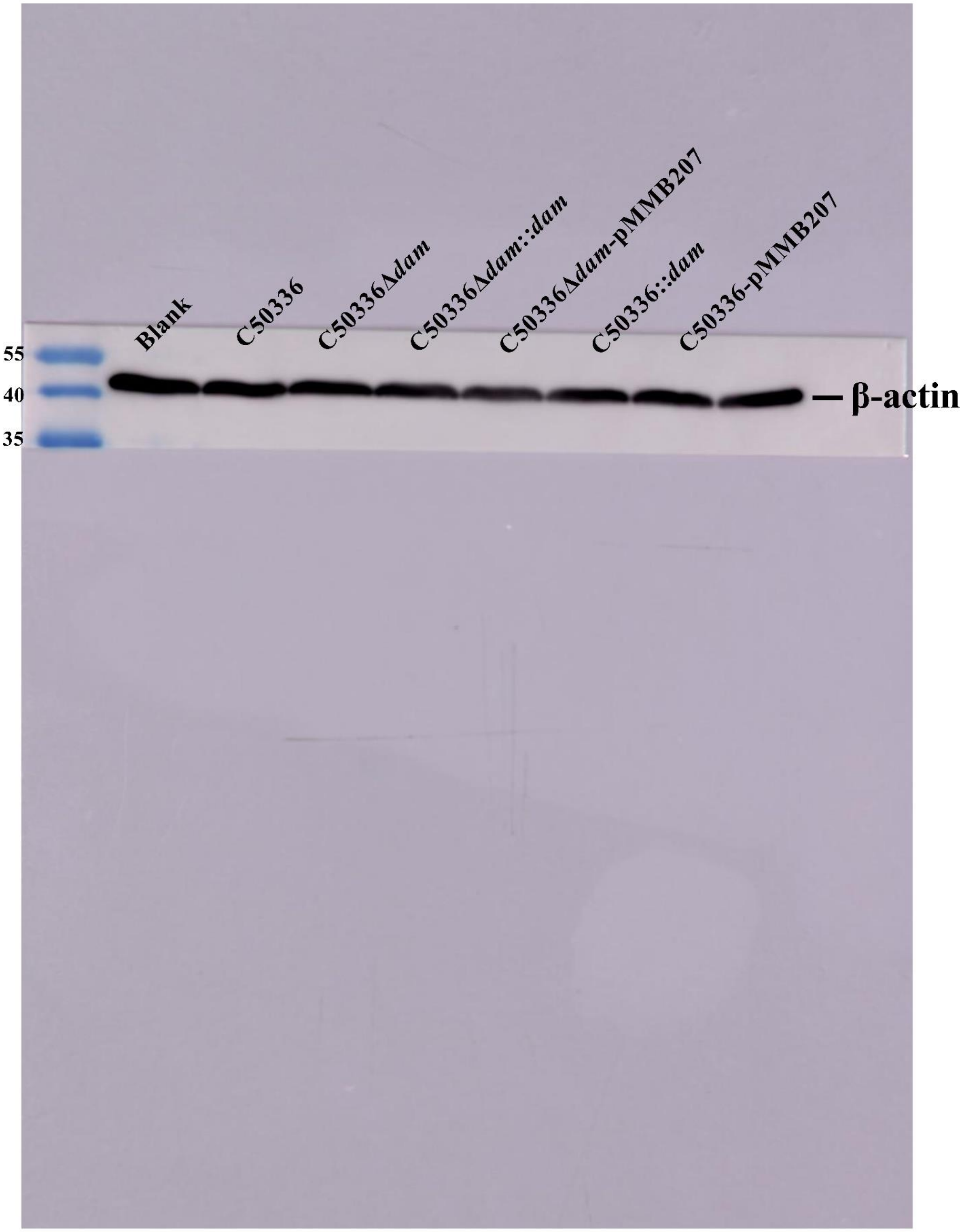
Blank
C50336
C50336Δ*dam*
C50336Δ*dam*::*dam*
C50336Δ*dam*-pMMB207
C50336::*dam*
C50336-pMMB207
55
40
35
β-actin

Figure S7

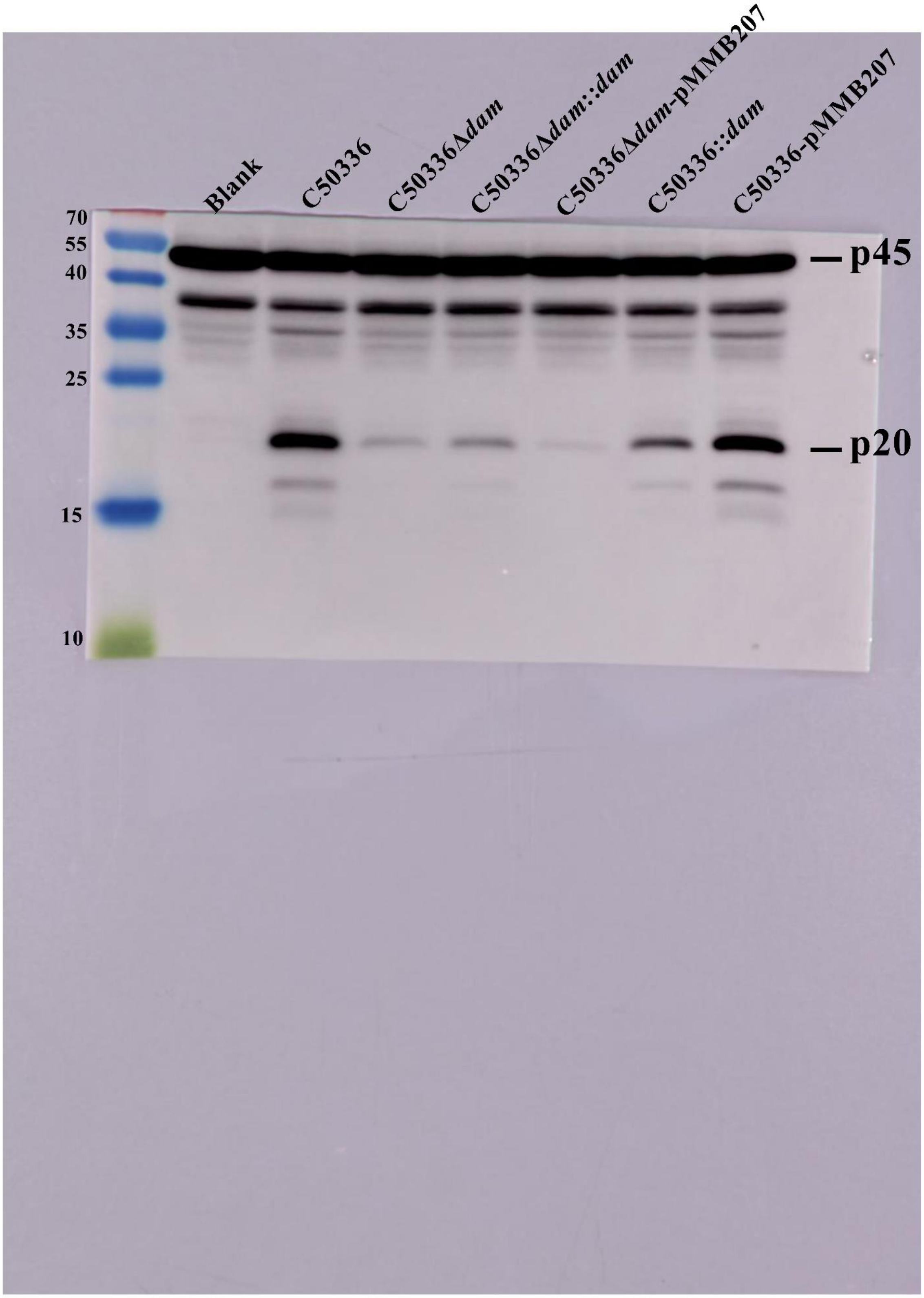
Blank
C50336
C50336Δdam
C50336Δdam::dam
C50336Δdam-pMMB207
C50336::dam
C50336-pMMB207
70
55
40
35
25
15
10
p45
p20

Figure S7

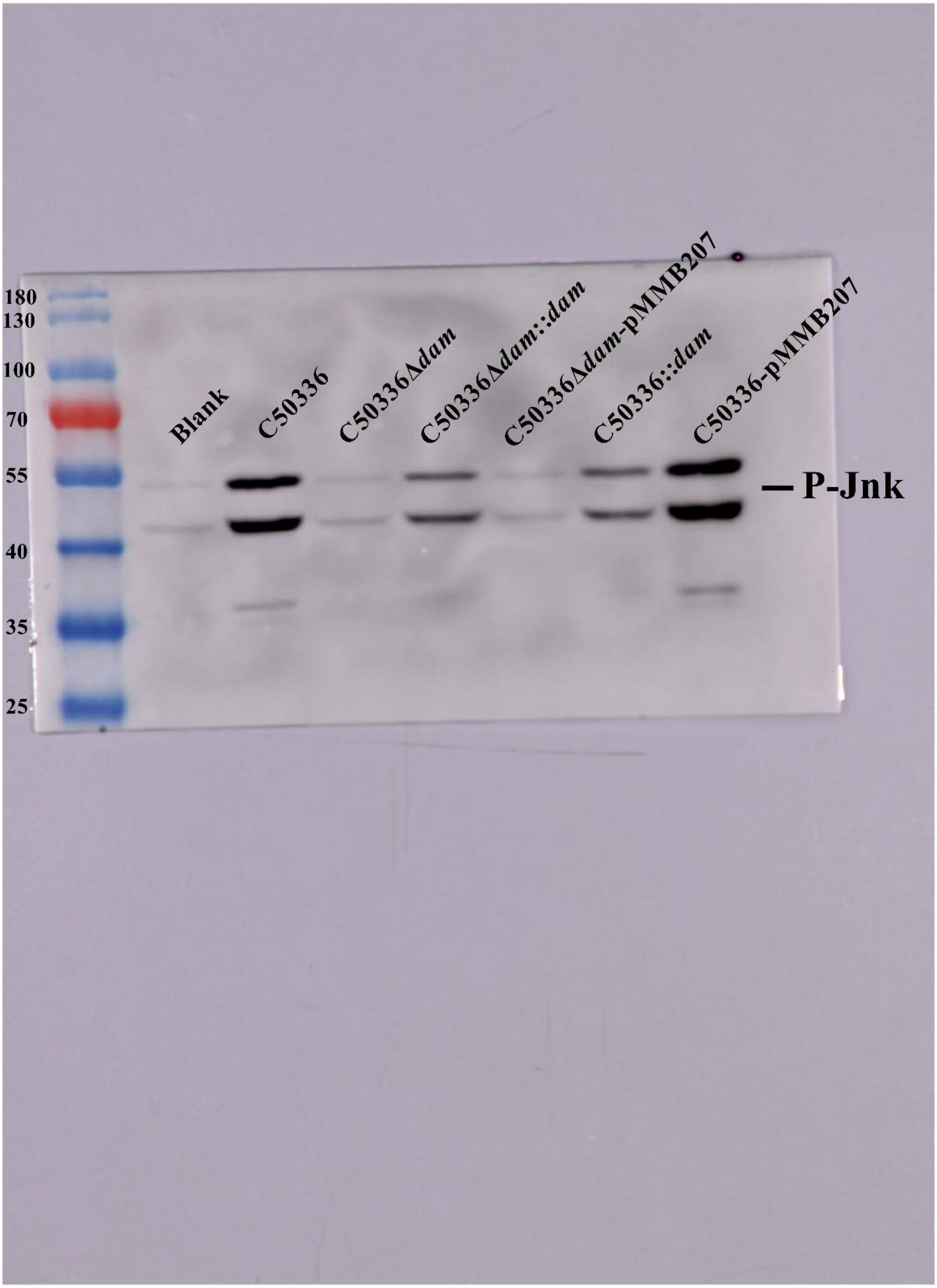
180
130
100
70
55
40
35
25
Blank
C50336
C50336Δ*dam*
C50336Δ*dam*::*dam*
C50336Δ*dam*-pMMB207
C50336::*dam*
C50336-pMMB207
P-Jnk

Figure S7

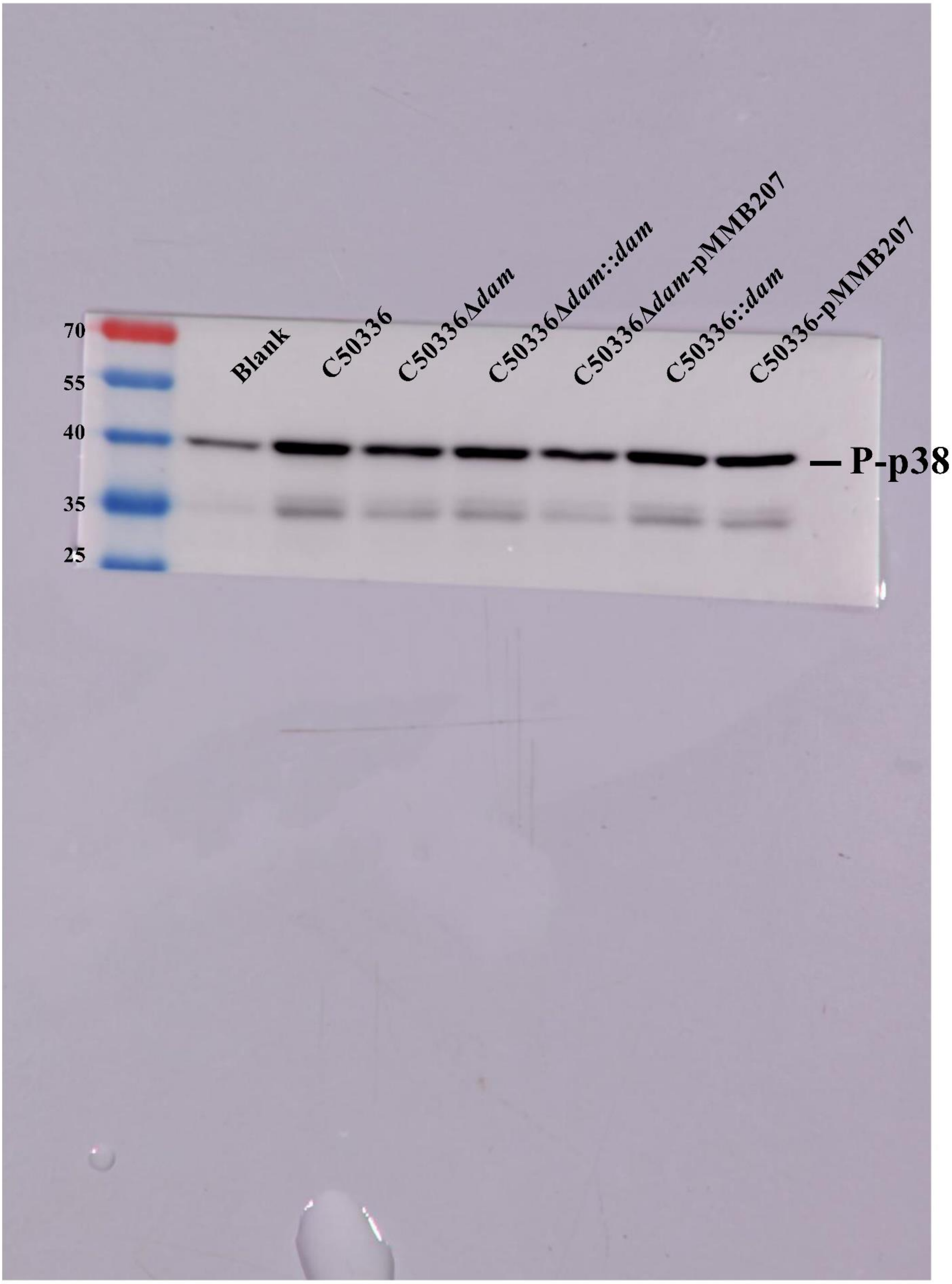
70
55
40
35
25
Blank
C50336
C50336Δ*dam*
C50336Δ*dam*::*dam*
C50336Δ*dam*-pMMB207
C50336::*dam*
C50336-pMMB207
P-p38

Figure S7

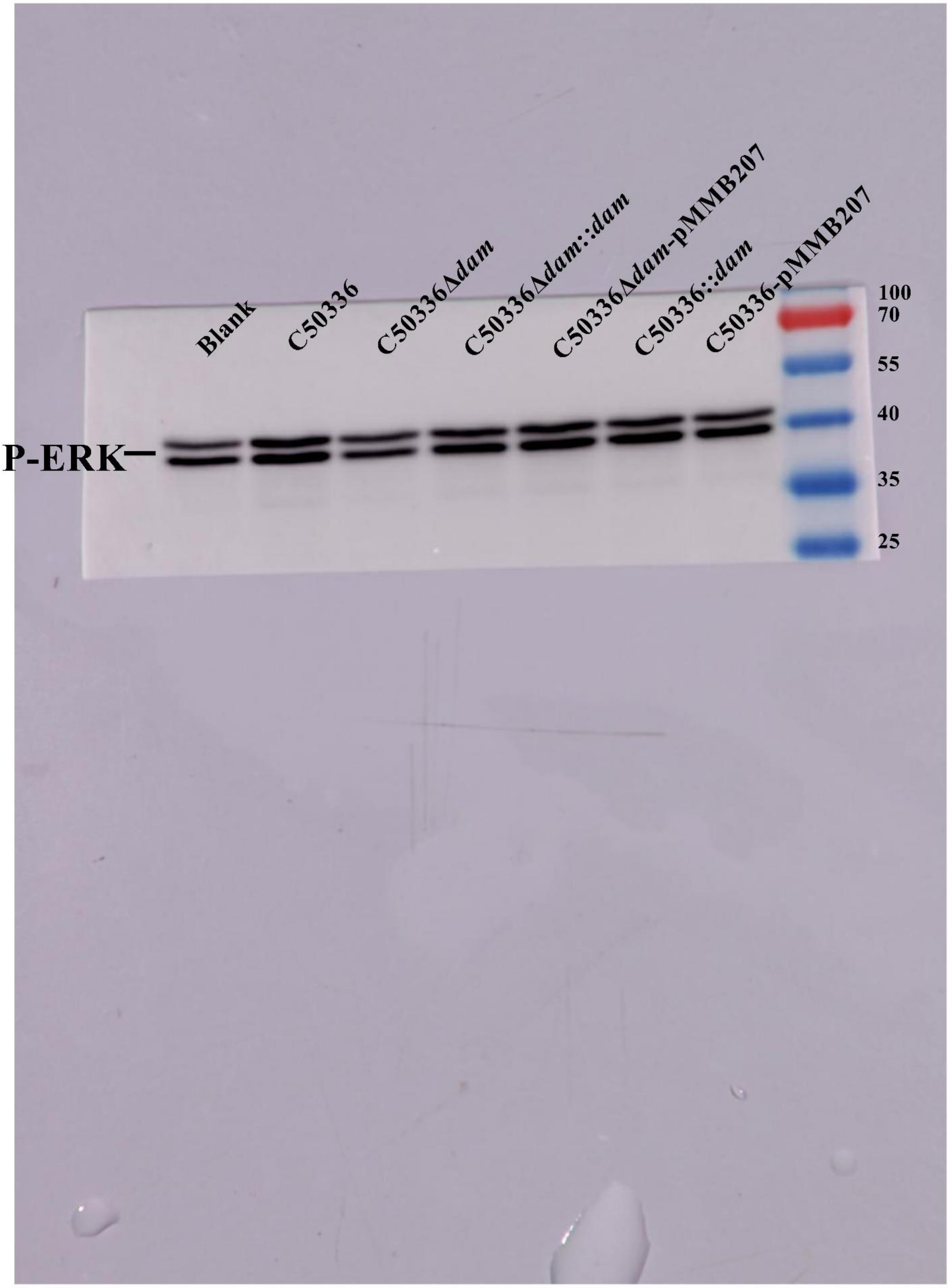
Blank
C50336
C50336Δdam
C50336Δdam::dam
C50336Δdam-pMMB207
C50336::dam
C50336-pMMB207
100
70
55
40
35
25
P-ERK

Figure S7

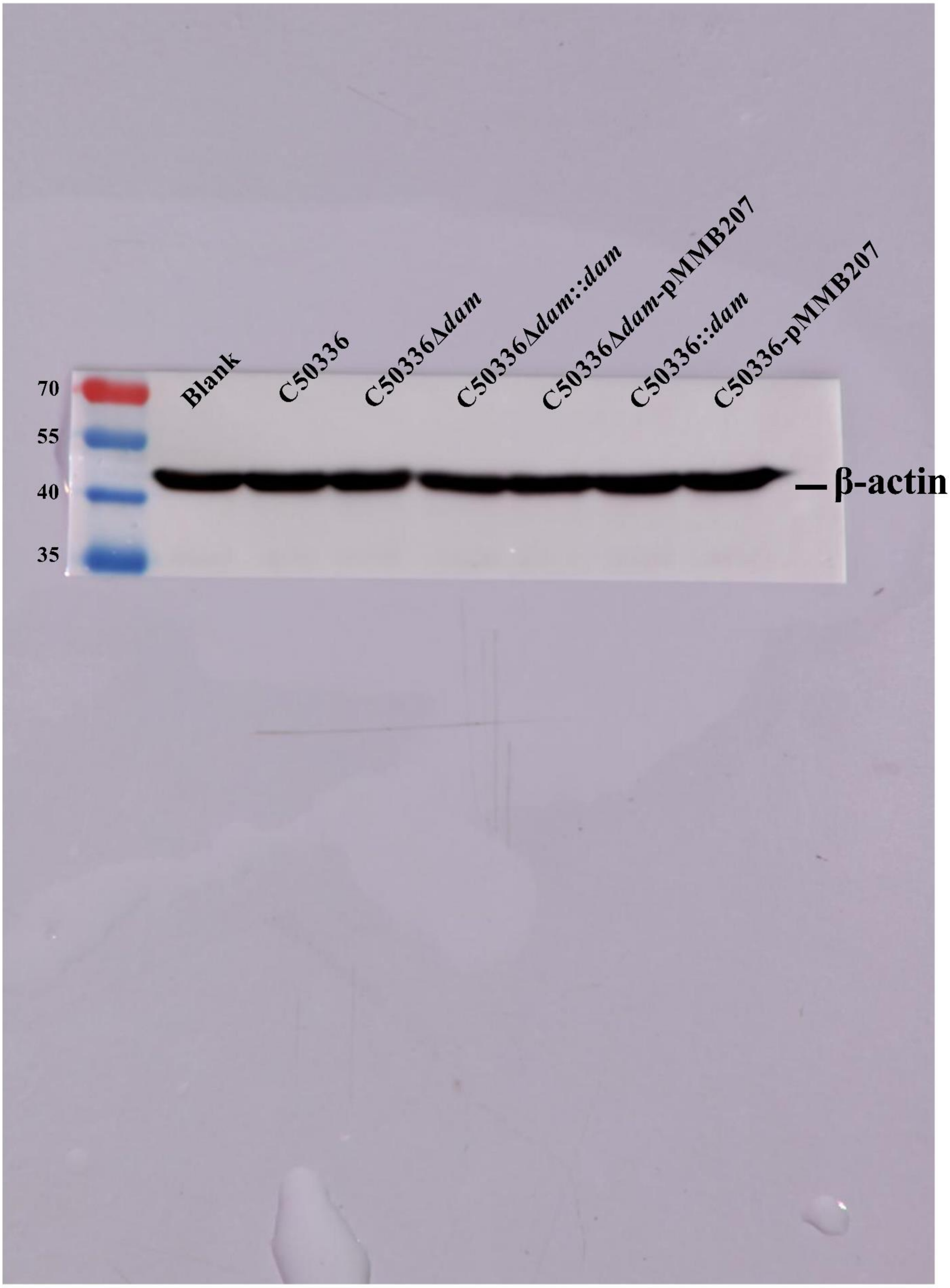
Blank
C50336
C50336Δdam
C50336Δdam::dam
C50336Δdam-pMMB207
C50336::dam
C50336-pMMB207
70
55
40
35
β-actin

Figure S8

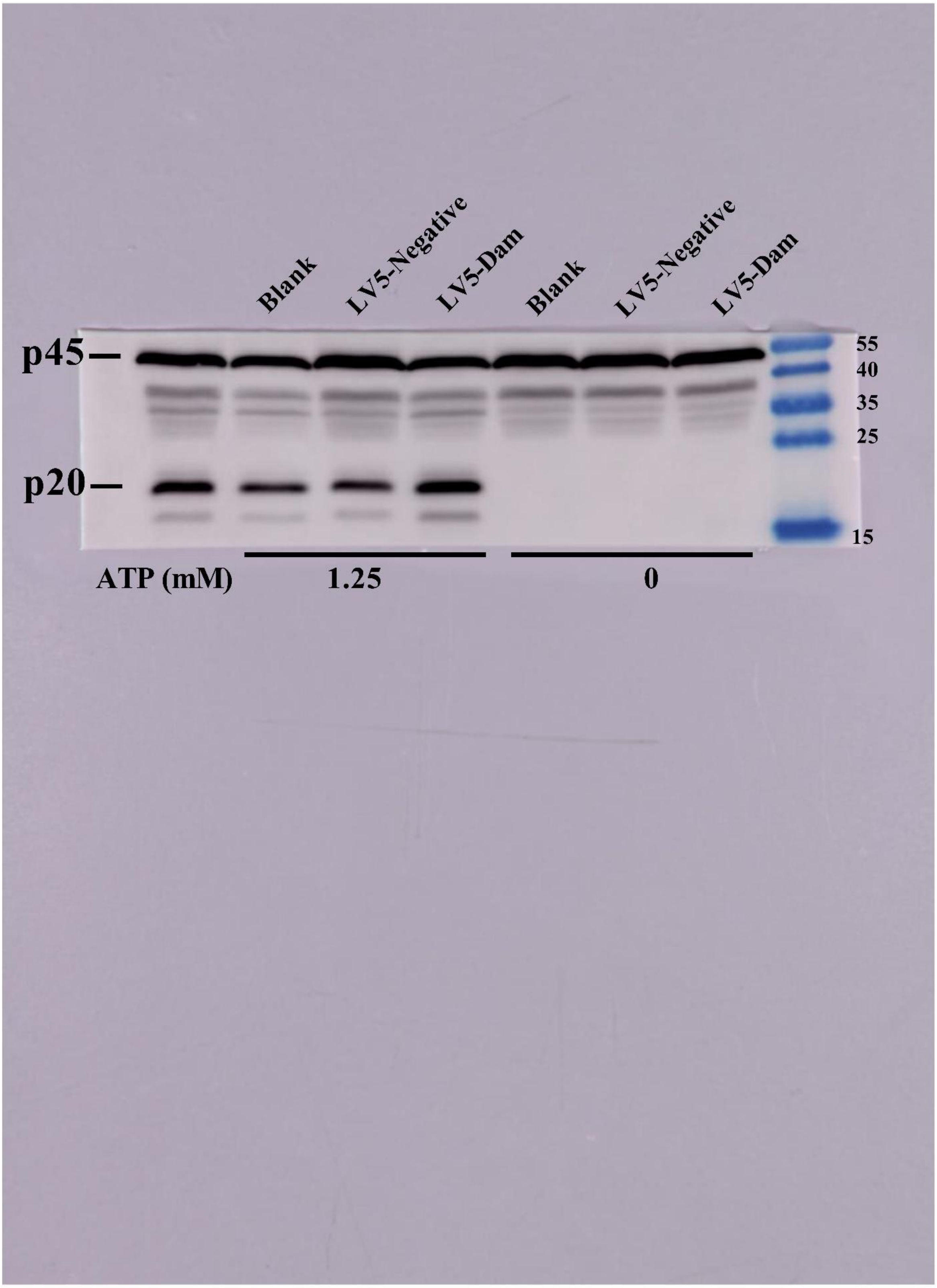

Blank
LV5-Negative
LV5-Dam
Blank
LV5-Negative
LV5-Dam
p45
p20
55
40
35
25
15
ATP (mM)
1.25
0

Figure S8

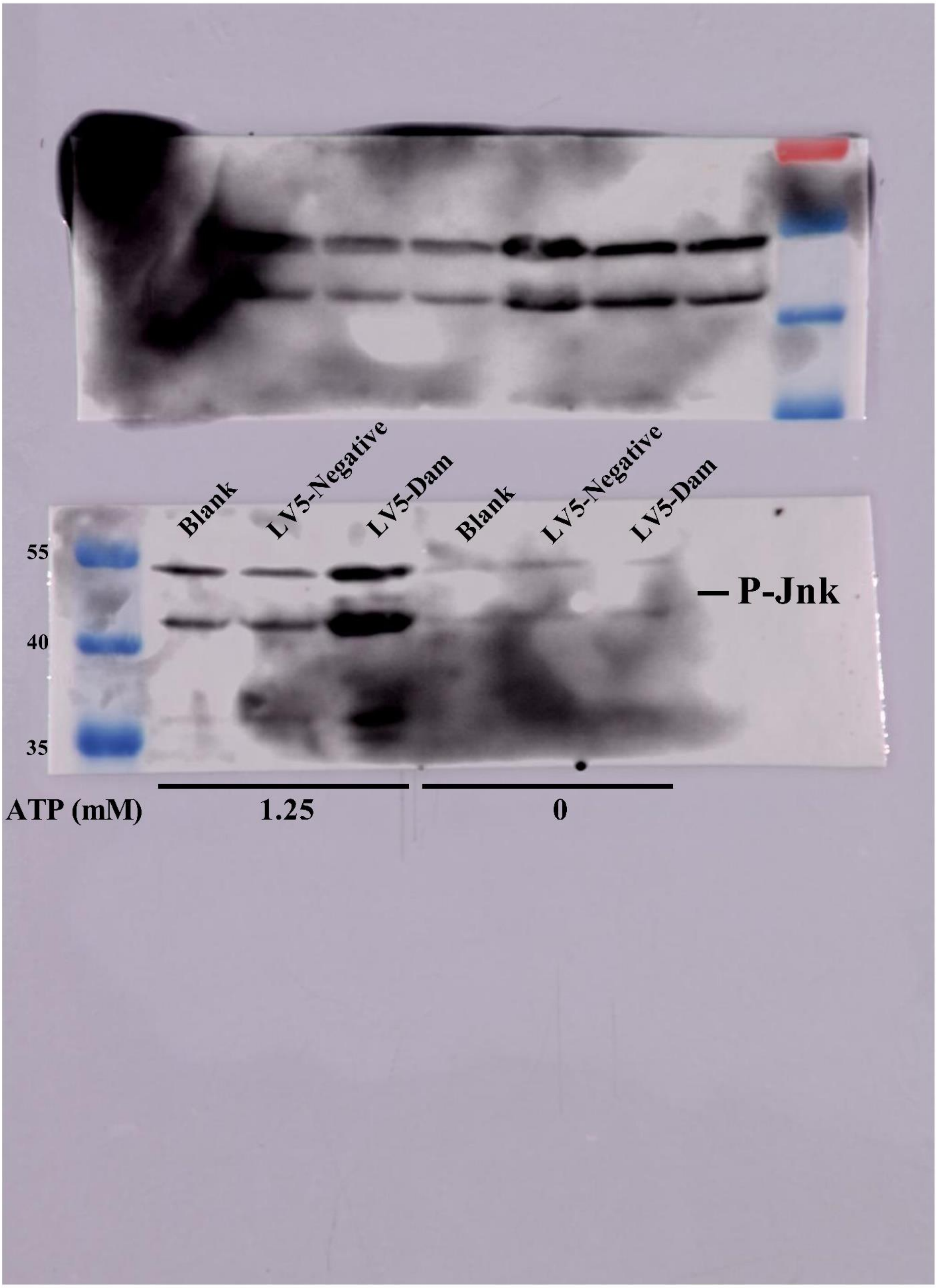

Blank
LV5-Negative
LV5-Dam
Blank
LV5-Negative
LV5-Dam
55
40
35
P-Jnk
ATP (mM)
1.25
0

Figure S8

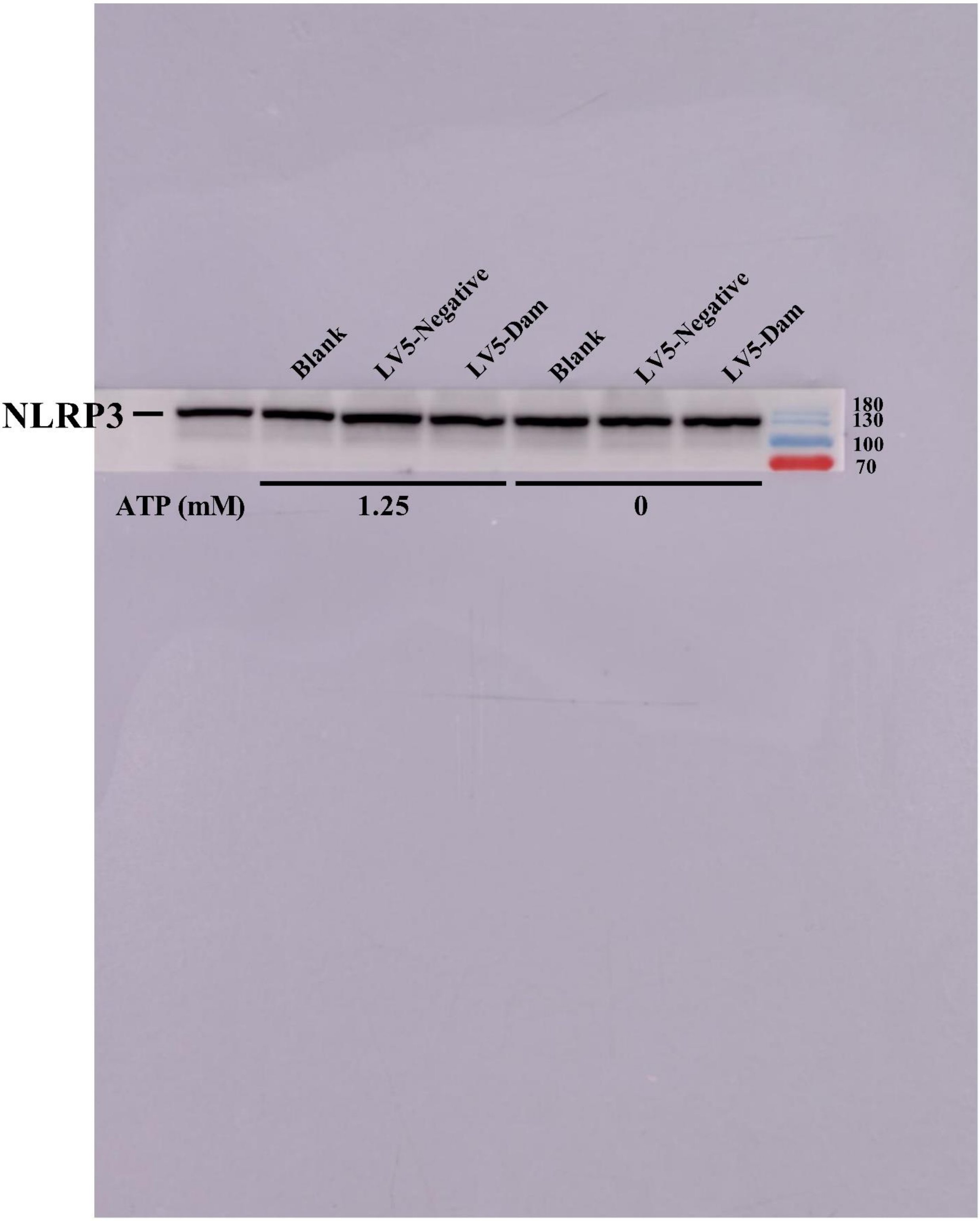
Blank
LV5-Negative
LV5-Dam
Blank
LV5-Negative
LV5-Dam
NLRP3
180
130
100
70
ATP (mM)
1.25
0

Figure S8

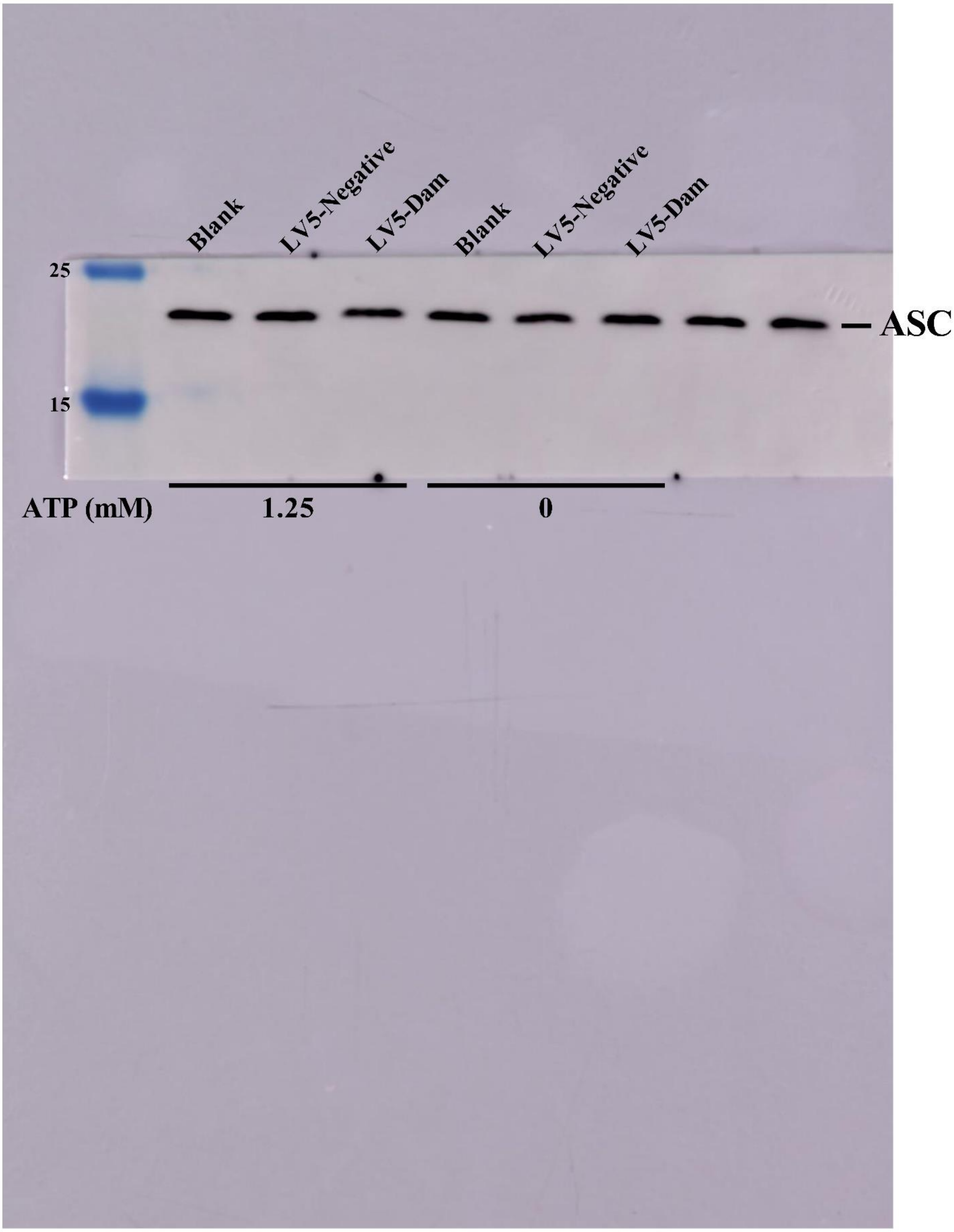
Blank
LV5-Negative
LV5-Dam
Blank
LV5-Negative
LV5-Dam
25
15
ASC
ATP (mM)
1.25
0

Figure S8

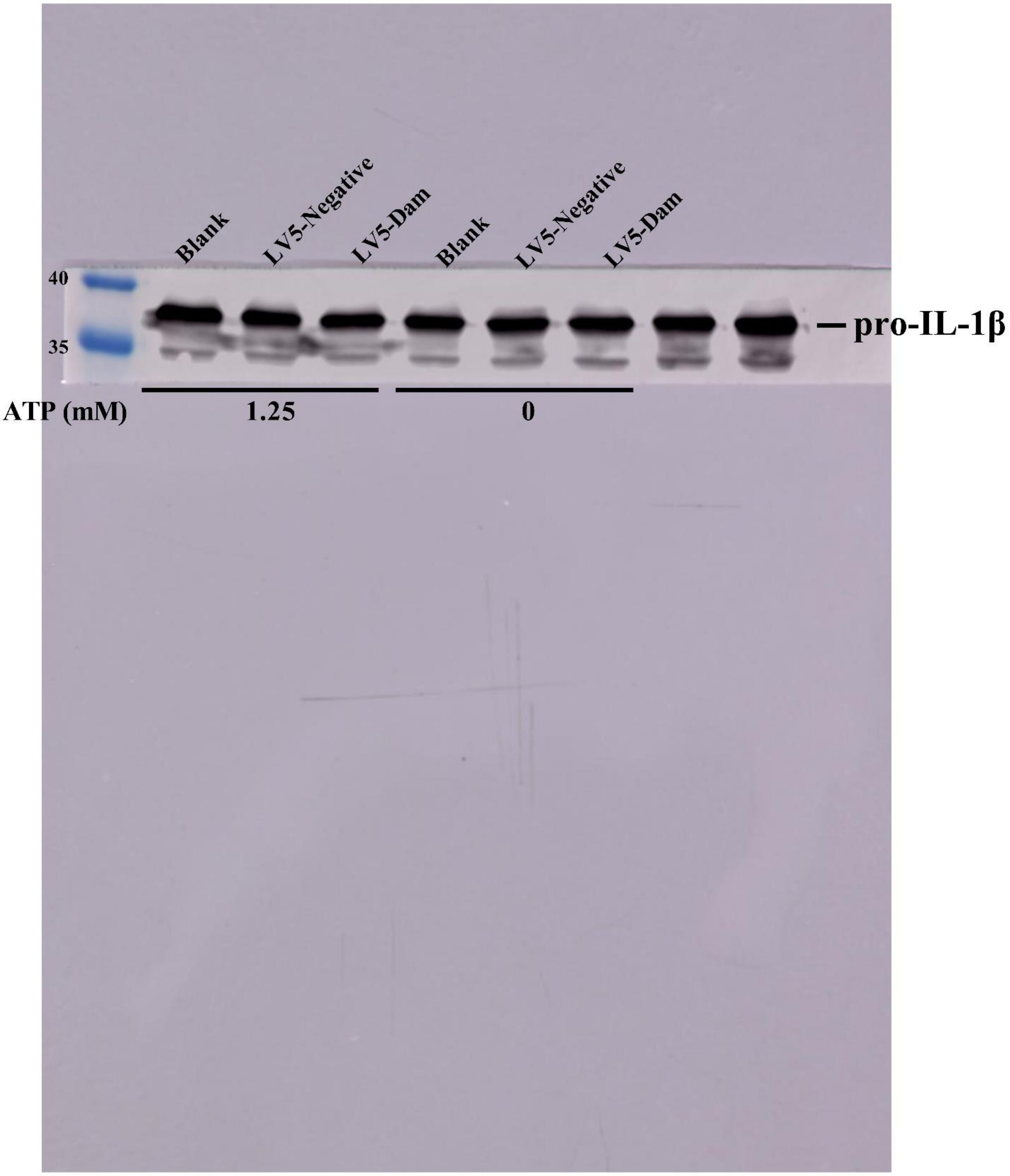

Blank
LV5-Negative
LV5-Dam
Blank
LV5-Negative
LV5-Dam
40
35
pro-IL-1β
ATP (mM)
1.25
0

Figure S8

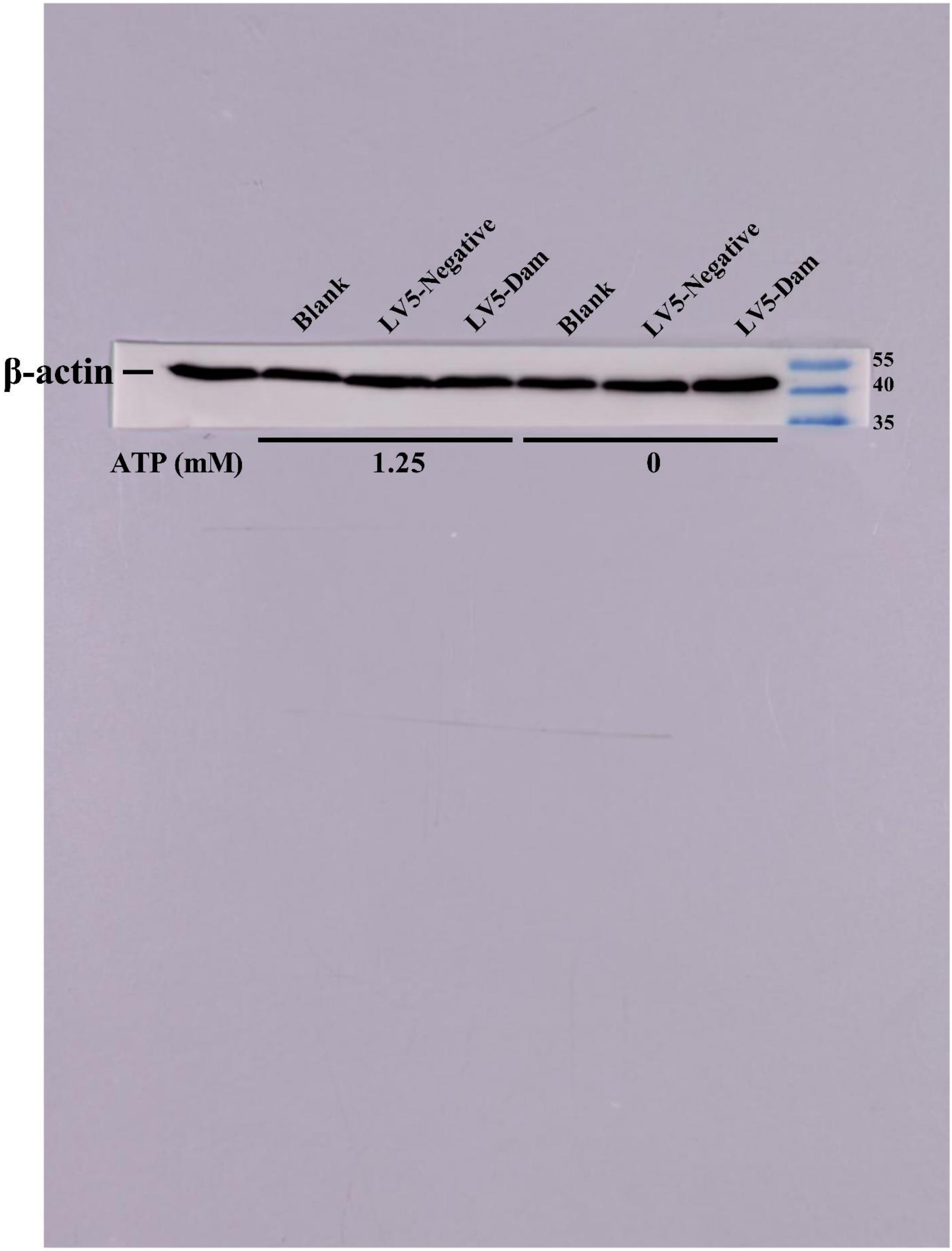
Blank
LV5-Negative
LV5-Dam
Blank
LV5-Negative
LV5-Dam
β-actin
55
40
35
ATP (mM)
1.25
0

Supplemental figure legends

Fig. S1 Growth curves of *dam* (**a**), *invC* (**b**), *hilD* (**c**), *prgH* (**d**), and *spaN* (**e**) gene deletion mutants, complementation, and overexpression strains. Bacteria were grown in liquid LB medium at 37 °C for 12 h with agitation, and the OD_{600} values of triplicate cultures in LB medium were determined in 1-h intervals.

Fig. S2 Two rounds of screening to identify the genes involved in regulating inflammasome activation *in vitro*. J774A.1 cells were pre-treated with LPS (1 μg/mL, 5 h) and then infected with candidate transposon mutants at an MOI of 20 for 4 h, uninfected cells was used as a negative control (Blank). The activation of caspase-1 (p20) was examined via western blot. β-actin was blotted as a loading control. Molecular mass markers in kDa are indicated on the left.

Fig. S3 Deletion mutants of *dam*, *invC*, *prgH*, and *spaN* failed to induce inflammasome activation. J774A.1 cells were pre-treated with LPS (1 μg/mL, 5 h) and then infected with WT strain C50336 and *dam*, *invC*, *hilD*, *prgH*, and *spaN* gene deletion mutants at an MOI of 20 for 4 h, uninfected cells was used as a negative control (Blank). The activation of caspase-1 (p20) was examined via western blot. β-actin was blotted as a loading control. Molecular mass markers in kDa are indicated on the left.

Fig. S4 Deletion of *prgH* did not influence the synthesis of inflammasome components. C57BL/6 BMDMs were pre-treated with LPS (1 μg/mL) for 5 h (untreated and uninfected BMDMs was used as a negative control, Blank LPS-), and then infected with C50336, C50336Δ*prgH*, C50336Δ*prgH*::*prgH*, C50336Δ*prgH*-pMMB207, C50336::*prgH*, or C50336-pMMB207 at an MOI of 20 for 4 h, uninfected BMDMs

was used as another negative control (Blank LPS+). Bacteria bearing pMMB207 plasmids were cultured with IPTG (0.5 mM). The expression of caspase-1, NLRP3, NLRC4, ASC, and pro-IL-1β were analyzed by immunoblotting. β-actin was blotted as a loading control. Molecular mass markers in kDa are indicated on the left.

Fig. S5 Overexpression of Dam inhibited inflammasome activation. C57BL/6 BMDMs were pre-treated with LPS (1 μg/mL) for 5 h (untreated and uninfected BMDMs was used as a negative control, Blank LPS-), and then infected with C50336, C50336Δ*dam*, C50336Δ*dam*::*dam*, C50336Δ*dam*-pMMB207, C50336::*dam*, or C50336-pMMB207 at an MOI of 20 for 4 h, uninfected BMDMs was used as another negative control (Blank LPS+). Bacteria bearing pMMB207 plasmids were cultured with IPTG (0.5 mM). The expression of caspase-1, NLRP3, NLRC4, ASC, and pro-IL-1β were analyzed by immunoblotting. β-actin was blotted as a loading control. Molecular mass markers in kDa are indicated on the left.

Fig. S6 The ability of the *dam* complementation strain and overexpression strain cultured without IPTG to activate the inflammasome was improved. C57BL/6 BMDMs were pre-treated with LPS (1 μg/mL) for 5 h (untreated and uninfected BMDMs was used as a negative control, Blank LPS-), and then infected with C50336, C50336Δ*dam*, C50336Δ*dam*::*dam*, C50336Δ*dam*-pMMB207, C50336::*dam*, or C50336-pMMB207 at an MOI of 20 for 4 h uninfected BMDMs was used as another negative control (Blank LPS+). Bacteria bearing pMMB207 plasmids were cultured without IPTG. The expression of caspase-1, NLRP3, NLRC4, ASC, and pro-IL-1β were analyzed by immunoblotting. β-actin was blotted as a loading control. Molecular mass markers in kDa are indicated on the left.

Fig. S7 The deletion of Dam blocked the Jnk pathway in infected J774A.1 cells. J774A.1 cells were pre-treated with LPS (1 μg/mL, 5 h) and then infected with C50336, C50336Δ*dam*, C50336Δ*dam*::*dam*, C50336Δ*dam*-pMMB207, C50336::*dam*, or C50336-pMMB207 at an MOI of 20 for 4 h, uninfected cells was used as a negative control (Blank). Bacteria bearing pMMB207 plasmids were cultured without IPTG. The activation of caspase-1, phosphorylated Jnk (P-Jnk), phosphorylated p38 (P-p38), and phosphorylated ERK1/2 (P-ERK1/2) were analyzed by immunoblotting. β-actin was blotted as a loading control. Molecular mass markers in kDa are indicated on the left.

Fig. S8 Dam independently promoted NLRP3 inflammasome activation. J774A.1 cells were transduced with LV5-Dam or LV5-negative lentivirus, untreated cells was used as a negative control (Blank). Cells were then pre-treated with LPS (1 μg/mL, 5 h) and stimulated with or without ATP (1.25 mM) for 1 h. The expression of caspase-1, NLRP3, ASC, pro-IL-1β, and P-Jnk were analyzed by immunoblotting. β-actin was blotted as a loading control. Molecular mass markers in kDa are indicated on the left.
